# Structure-guided semi-rational design of an imine reductase for enantio-complementary synthesis of pyrrolidinamine†

**DOI:** 10.1039/d2sc07014f

**Published:** 2023-03-23

**Authors:** Jun Zhang, Yaqing Ma, Fangfang Zhu, Jinping Bao, Qiaqing Wu, Shu-Shan Gao, Chengsen Cui

**Affiliations:** a Tianjin Institute of Industrial Biotechnology, Chinese Academy of Sciences Tianjin 300308 China gaoss@tib.cas.cn cuichs@tib.cas.cn; b School of Life Science, Hebei University Baoding 071002 China; c CAS Key Laboratory of Microbial Physiological and Metabolic Engineering, State Key Laboratory of Microbial Resources, Institute of Microbiology, Chinese Academy of Sciences Beijing 100101 China; d College of Biotechnology, Tianjin University of Science and Technology Tianjin 300457 China; e National Technology Innovation Center of Synthetic Biology Tianjin 300308 China

## Abstract

In this study, engineered imine reductases (IREDs) of IRED M5, originally from *Actinoalloteichus hymeniacidonis*, were obtained through structure-guided semi-rational design. By focusing on mutagenesis of the residues that directly interact with the ketone donor moiety, we identified two residues W234 and F260, playing essential roles in enhancing and reversing the stereoselectivity, respectively. Moreover, two completely enantio-complementary variants S241L/F260N (*R*-selectivity up to 99%) and I149D/W234I (*S*-selectivity up to 99%) were achieved. Both variants showed excellent stereoselectivity toward the tested substrates, offering valuable biocatalysts for synthesizing pyrrolidinamines. Its application was demonstrated in a short synthesis of the key intermediates of potential drug molecules leniolisib and JAK1 inhibitor 4, from cheap and commercially available pro-chiral *N*-Boc-piperidone 1 (2 and 3 steps, respectively).

## Abstract 2

In this study, engineered imine reductases (IREDs) of IRED M5, originally from *Actinoalloteichus hymeniacidonis*, were obtained through structure-guided semi-rational design.

## Graphical abstract

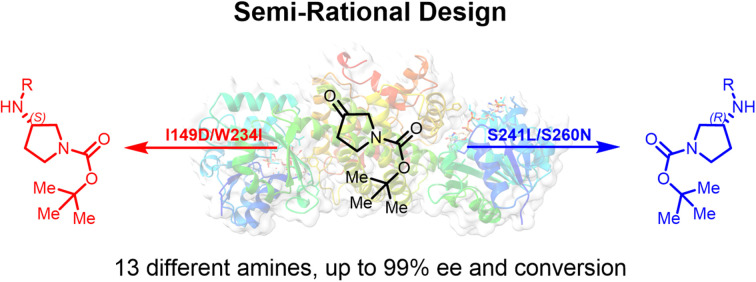

## Introduction

Chiral amines are used as privileged structural motifs in the pharmaceutical industry.^1^ Classical chemical processes typically rely on resolution of racemic amines or nucleophilic displacement of activated chiral alcohols.^2^ Catalytic processes of asymmetric reductive amination are also available. However, they suffer from side reactions or the use of expensive transition-metal complexes with chiral ligands that can be difficult to obtain and remove.^3,4^ A biocatalyst provides a new choice for green and efficient synthesis of chiral amines.^5,6^ NADPH dependent imine reductases (IREDs) are one of the most attractive biocatalysts for the synthesis of chiral amines.^7–10^ Since IREDs were identified by Nagasawa and co-workers in 2010, they have been reported to reduce structurally different imines and synthesize diverse chiral amines.^11^ A subfamily of IREDs is fascinating for creating secondary or tertiary amines by reductive amination of pro-chiral ketones or aldehydes and amines in equal stoichiometric amounts, which is the most straightforward strategy for the generation of alkylamines.^12^ Notably, IREDs have been reported to accept a wide range of ketones and amines^12–27^ and successfully applied to the commercial manufacture of LSD1 inhibitor GSK2879552 and JAK1 inhibitor abrocitinib.^28,29^

Enantiomers frequently behave very differently in biological systems due to their different shapes in 3-dimensional space in drug molecules; thus producing both enantiomers is an important task in the pharmaceutical industry.^30,31^ Since the discovery of these *R*- and *S*-selective IREDs, they have been capable of the asymmetric reduction of various prochiral imine substrates with a high degree of stereoselectivity.^11,32,33^ According to a large number of reported substrate screenings, IREDs are also selectively preferred in IRED catalytic reductive amination reactions.^12,18,25^ Therefore, to access both the optically pure enantiomers, it is often necessary to screen an extensive enzyme library to obtain complementary stereoselective IREDs.^13,16,24–27^ However, the target natural enzymes are often not suitable for industrial application because of their low catalytic efficiency or thermostability. In this case, large mutagenesis efforts for complementary stereoselective enzymes are needed to meet the requirements of industrial processes.^26,28,29^ Alternatively, rationally engineering an enzyme, which shows high catalytic efficiency for target substrates, to obtain enantio-preference enzymes is a highly efficient method.^34–38^ Rational design usually focuses on a minority of potential amino acids, which can significantly reduce the number of mutants to be investigated.^37,38^ Although several advances focusing on tuning enzymatic catalytic efficiency have been reported,^26,28,29^ rational design of IREDs for stereo-divergent reductive amination to synthesize both stereoisomers is relatively undeveloped.

**Fig. 1:**
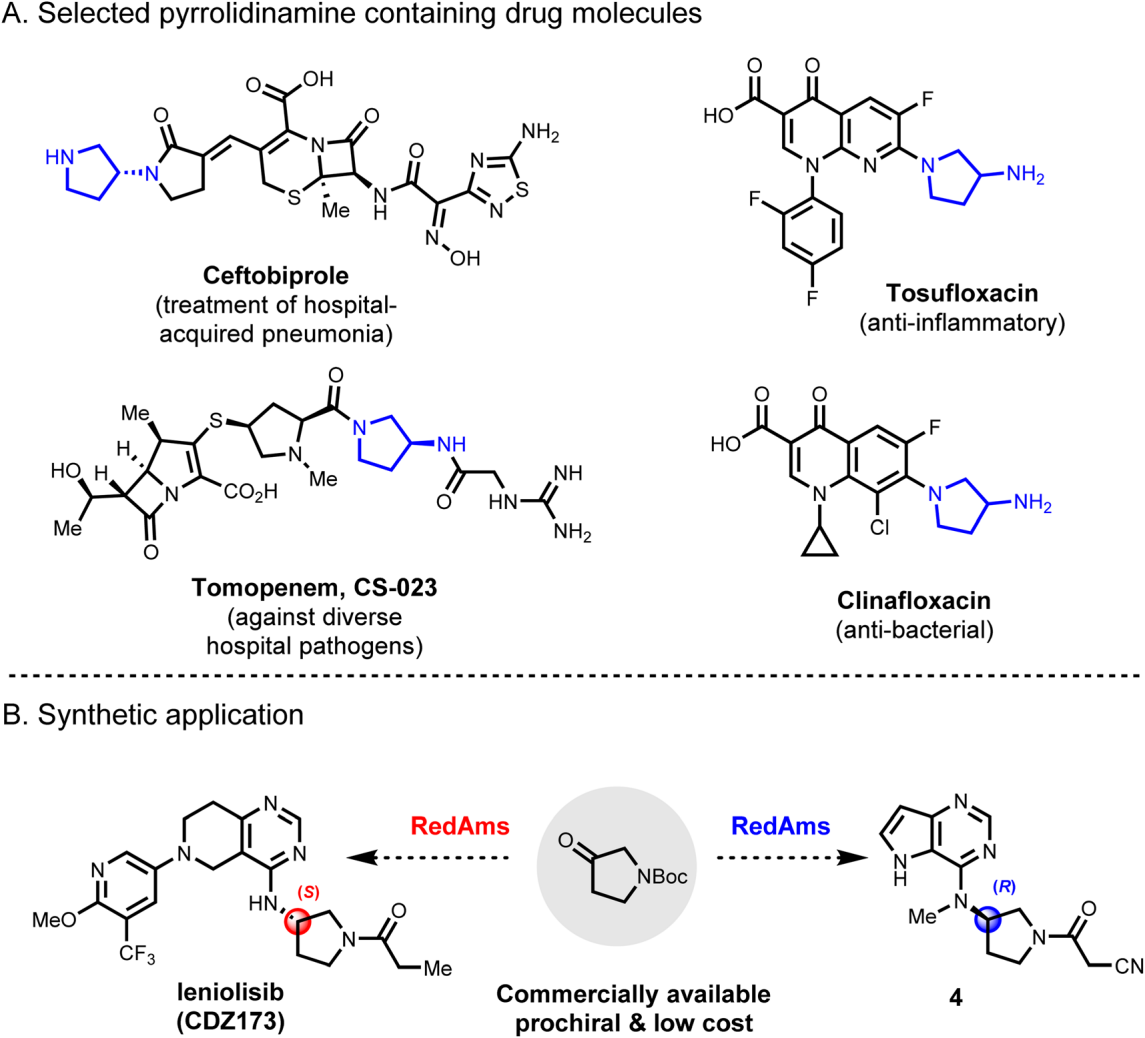
Pyrrolidinamine containing drug molecules and its synthetic application.

Pyrrolidinamines are important functional groups in many pharmaceuticals, including ceftobiprole,^30^ tomopenem (CS-023),^31,39^ leniolisib,^40^ clinafloxacin^41^ and tosufloxacin^42^ (Fig. 1A). Recently, asymmetric imine reduction of pyrrolidinamine building blocks of pharmaceuticals using IRED has been well studied.^43–45^ We also reported a wild type IRED IR-G36 from *A. hymeniacidonis*, which was engineered to obtain a mutant M5 producing a diverse spectrum of piperidine and azepane alkylamines through reductive amination, with excellent catalytic efficiency, *R*-selectivity, and thermostability.^26^ However, when using *N*-Boc-3-pyrrolidone 1 as a ketone donor, M5 displayed poor stereoselectivity with an ee value of 44% (*R*), despite high catalytic efficiency with substrate loading up to 19 g L^−1^. Thus, M5 has emerged as an attractive starting point for exploring the preparation of both *R*- and *S*-pyrrolidinamines. Given the importance of pyrrolidinamines, we performed the mechanism- and structure-guided semi-rational design of IRED-M5 to acquire variants with a high degree of enantio-complementary stereoselectivities for efficient synthesis of a series of pyrrolidinamines. Based on the efficient platform, chemoenzymatic synthesis of key intermediates of JAK1 inhibitor 4 (ref. 46) and leniolisib was performed with fewer steps compared to previous chemical methods using pro-chiral starting material 1 (Fig. 1B).

Herein, we report an IRED-catalyzed stereo-divergent reductive amination strategy for synthesizing key intermediates, pyrrolidiamines, enabled by the directed evolution of IREDs. This enzymatic reaction was key to delivering a new process starting from a pro-chiral compound with reduced cost and fewer steps for the synthesis of enantio-complementary pyrrolidiamines, key intermediates for both leniolisib and JAK1 inhibitor 4, respectively.

### Semi-rational design

According to the catalytic mechanism of IRED, the carbonyl substrate couples to the amine substrate to form the imine intermediate after deprotonation and elimination of a water molecule. Then, the imine is reduced by the cofactor NADPH to give the final chiral amine product. As such, the imine intermediate may bind in two distinct orientations relative to Pro-(*S*) or Pro-(*R*), differing by a rotation of ∼180 deg around the axis along the C=N bond. Thus, the amino acids that interact directly with the ketone moiety are the most influential ones for controlling stereoselectivity, which is supported by previous studies showing that different amine donors have less effect on the stereoselectivity of IRED catalyzed reactions in their substrate scope studies.^12,18,25,26^

**Fig. 2:**
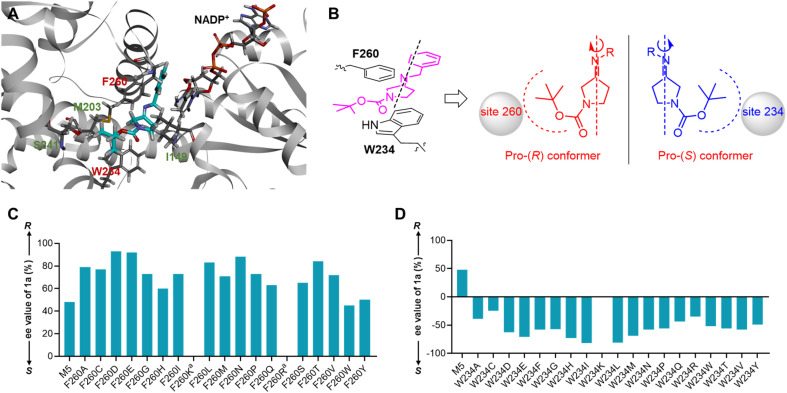
(A) Docking the imine intermediate of reductive amination of 1 and a into the M5 cavity shows five residues that directly interact with the *N*-Boc-piperidine moiety. (B) Two key residues W234 and F260 generate steric hindrance for the rotation of the *N*-Boc-pyrrolidine moiety causing neither *Si*- nor *Re*-face orientating to the co-factor NADPH. (C) and D) Enantioselectivity of M5 and its mutants at sites 234 and 260 in the reductive amination of 1 and a. ^a^ee was not determined because the mutants were almost inactivated.

In this study, *N*-Boc-pyrrolidone 1 and benzylamine a were initially chosen as the model substrates for M5-catalyzed reductive amination. In order to find the potential mutation sites, the imine intermediate was docked into the crystal structure of M5 using the Discovery Studio. The docking result indicated that the *N*-Boc-piperidine moiety faces the side opening of the tunnel (Fig. 2A), which is consistent with the relative position of ketone in the reported quaternary complex of *At*RedAm.^46^ Thus, we assumed that the residues controlling stereoselectivity were located near the side opening of the active pocket.

Analysis of the docking result indicated that five amino acid residues I149, M203, W234, S241, and F260, were identified to interact directly with the *N*-Boc-piperidine moiety (Fig. 2A). Noteworthily, two residues W234 and F260 are located on either side of the *N*-Boc-piperidine moiety respectively, and their large side chains generate steric hindrance to the bulky *N*-Boc group, which restrains the imine intermediate to rotate freely along the axis (Fig. 2B), suggesting that they are important in providing a steric constraint for controlling the face for hydride delivery. Thus, a larger cavity at the tryptophan 234 or phenylalanine 260 positions could better facilitate access to the bulky *N*-Boc group. In other words, mutating W234 to a smaller amino acid might create more space for the predominant Pro-(*S*) conformer of the imine intermediate in the active site. Otherwise, exchanging F260 with a smaller amino acid might cause the predominant Pro-(*R*) conformer (Fig. 2B).

Subsequently, site-saturation mutagenesis was performed at positions 234 and 260, respectively, to investigate the stereoselectivities corresponding with the volume of the residue, and the result is shown in Fig. 2C and D. As expected, most of the mutants at position 260 enhanced *R*-selectivity except F260W, implying that exchanging large F260 with a smaller residue offers a large enough pocket for the bulky *N*-Boc group to improve the binding ability of the Pro-(*R*) conformer of the imine intermediate. Three mutations led to >85% *R*-selectivity, and the highest *R*-selective mutants were F260D (93% ee), F260E (92% ee), and F260N (88% ee). However, when F260 was replaced by two positively charged amino acids with long chains, the mutants F260K and F260R almost lost their catalytic activity (Fig. 2C). Mutations at position 234 are also in line with our expectations. Most of the mutants showed reversed stereoselectivity, and two mutants W234I and W234L showed the best *S-*selectivity with ee values of 82% and 81%, respectively. These results indicated that the residue at position 234 was crucial for the enantioselectivity *via* tuning the size of the stereospecificity pocket and acting as a switch for complementary stereoselectivities.

**Fig. 3:**
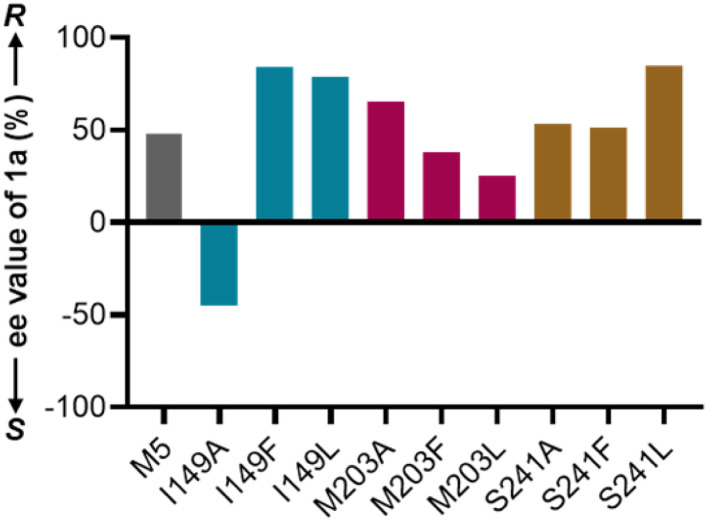
Enantioselectivity of M5 and its mutants in the reductive amination of 1 and a.

To further improve *R*- and *S*-stereoselectivity, the other three residues I149, M203, and S241 were investigated. Three amino acids of different sizes alanine (A), leucine (L), and phenylalanine (F) were chosen for site-specific mutagenesis over M5. As shown in Fig. 3, I149F, I149L, and S241L showed enhanced *R*-selectivity with ee values of 84%, 79%, and 85%, respectively. In contrast, the mutant I149A showed a reversed stereoselectivity with an ee value of 45% (*S*). However, the mutations of M203A, M203F, and M203L showed no *S*-stereoselectivity. All results indicate that residues that interact directly with the *N*-Boc-piperidine moiety have significant effects on stereoselectivity.

Both enantioselective variants were optimized utilizing combinational mutagenesis. First, we combined the *R*-stereoselective mutants F260D, F260E, and F260N with S241F and I149L, respectively. As a result, the combinatorial variants S241L/F260D, S241L/F260E and S241L/F260N showed excellent *R*-selectivity with an ee value of 99%, while the combinational variants of I149F and site F260 displayed dramatically decreased catalytic activity. Afterward, we investigated the combination of I149A and W234I to synthesize (*S*)-1a. Although the combinatorial variant I149A/W234I showed improved *S*-selectivity with an ee value of 97%, it did not meet the biocatalyst criteria for industrial application. To gain deeper insight into the contribution of site I149 on *S*-selectivity, we carried out saturation mutation on this hot position over W234I. To our delight, two variants I149D/W234I and I149H/W234I showed the best *S*-selectivity with ee values of 99% (Table 1). While all the double mutants showed comparable high enantioselectivities, their catalytic activities may vary widely. Thus, 5 mL-scale biotransformation started with *N*-Boc-3-pyrrolidinone 1 and benzylamine hydrochloride a (1.1 eq.) using purified enzymes (5 mg mL^−1^), and the results are assessed in Table 1. It was found that the catalytic activity of S241L/F260N was higher than that of S241L/F260D and S241L/F260E among the *R*-selective variants, with a conversion of 99%. The best *S*-selective variant was I149D/W234I with a conversion of 99%, which was about 50% higher than that of I149H/W234I.

**Table 1:** M5 mutant-catalyzed reductive amination for the synthesis of (*R*)- and (*S*)-1a. Reaction conditions: ketone 1 loading (100 or 110 mM), amines (1.1 equiv.), glucose (1.5 equiv.), GDH enzyme powder (1 mg mL^−1^), NADP^+^ (1 mM), purified enzyme (5 mg mL^−1^) in 5 mL of sodium phosphate buffer (100 mM, pH 7.0) at 30 °C, 220 rpm within 24 h

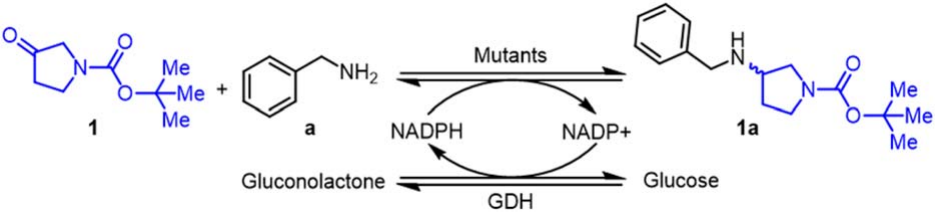				
Mutants	Substrate loading (mM)	Enzyme loading (mg mL^−1^)	Conv. (%)	ee
S241L/F260D	100	5	78	99%, *R*
S241L/F260E	100	5	85	99%, *R*
S241L/F260N	100	5	99	99%, *R*
I149D/W234I	100	5	99	99%, *S*
I149H/W234I	100	5	65	99%, *S*

### Shedding light on the mechanism of enantio-controlling

**Fig. 4:**
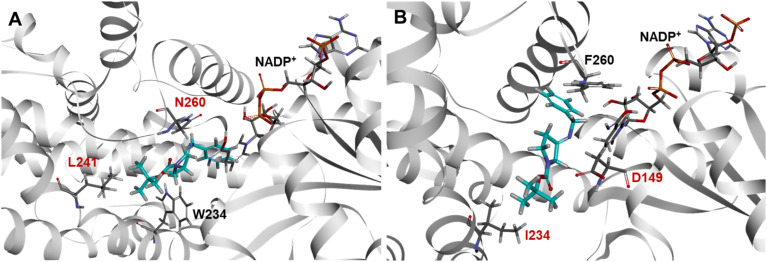
Depictions of the imine intermediate docked into the cavities of (A) S241L/F260N and (B) I149D/W234I.

To gain molecular insights into the stereo-divergent behavior, the imine intermediate was docked into the active sites of S241L/F260N and I149D/W234I, respectively (Fig. 4). Obviously, the smaller sidechain of asparagine at site-260 gives more space for the bulky *N*-Boc group and makes the imine intermediate more likely to stay with Pro-(*R*) conformations in the active site of mutant S241L/F260N to produce (*R*)-1a (83% yield, 99% ee). On the opposite side, when W234 is replaced with smaller isoleucine, it loses the steric restraints for the bulky *N*-Boc group, leading to the Pro-(*S*) conformer in the active site and a kinetic preference for the (*S*)-1a (84% yield, 99% ee).

### Exploration of substrate scope

**Fig. 5:**
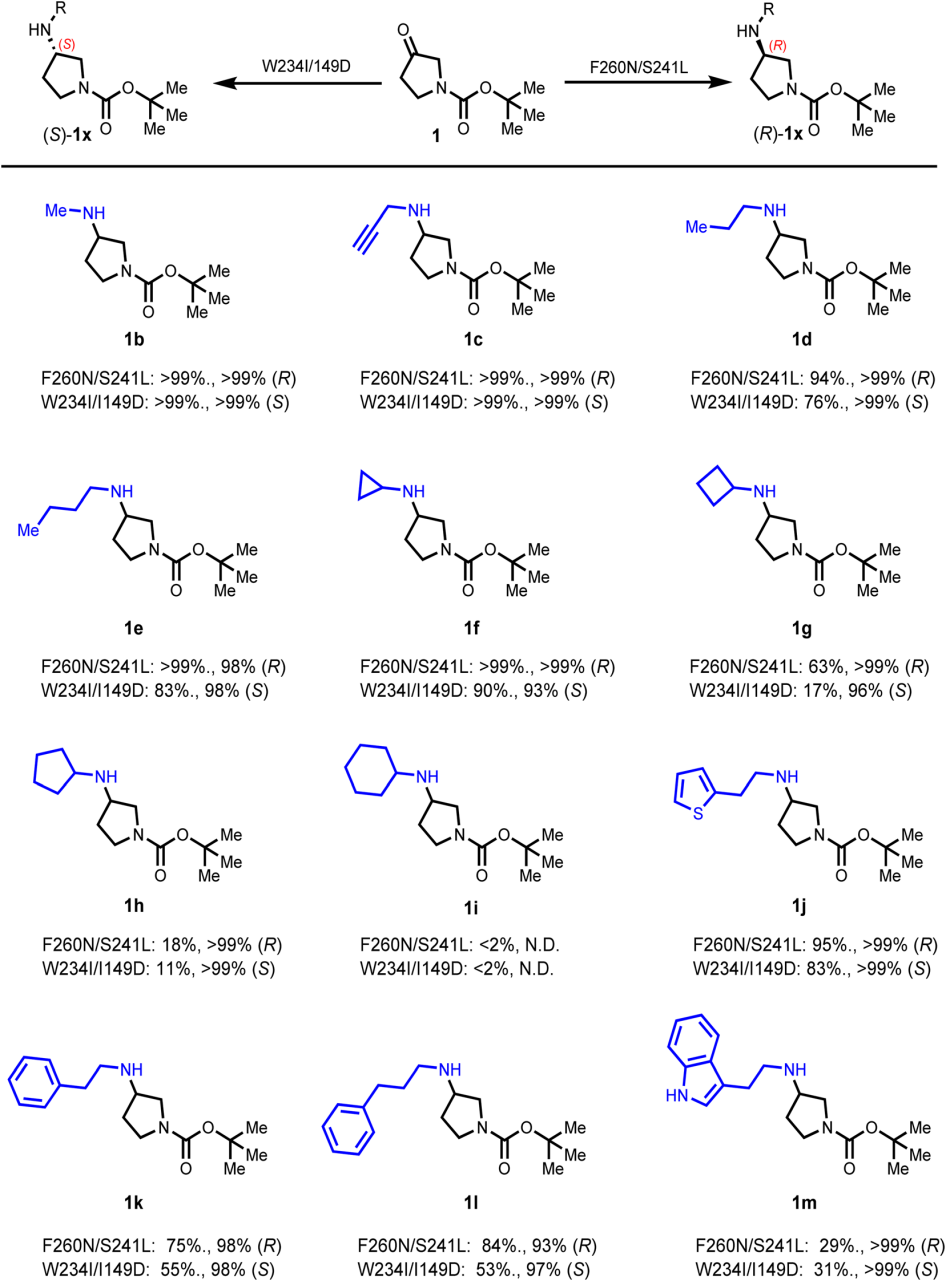
Preparative-scale reductive amination for the asymmetric synthesis of amines. Reaction conditions: ketone 1 loadings (100 mM for S241L/F260N and 110 mM for I149D/W234), amines (1.1 equiv.), glucose (1.5 equiv.), GDH enzyme powder (1.5 mg mL^−1^), NADP^+^ (1 mM), lyophilized cell extract of S241L/F260N or I149D/W234I (10 g L^−1^ wet cells weight) in 50 mL of sodium phosphate buffer (100 mM, pH 7.0) at 30 °C, 220 rpm within 24 h. N.D. not determined.

The successful design encouraged us to evaluate a panel of diverse amine donors with types and volumes using the two best enantio-complementary variants S241L/F260D and I149D/W234I (Fig. 5). Reductive aminations of *N*-Boc-piperidone 1 (100 mM or 110 mM) and 12 amines b–m (1.1 eq.) with various volumes were tested under the optimized conditions using cell-free extract (10 g L^−1^ wet cell weight). GDH was used for the NADPH recycling system and DMSO (20% v/v) was added as a co-solvent supporting the solubility of ketone 1 in the phosphate buffer (PBS) at pH 7.0. Although amine donors of different types and volumes were utilized, most reactions catalyzed by S241L/F260N and I149D/W234I showed excellent enantioselectivity with ee values (>99%, *R*) and (>99%, *S*), respectively, which again indicated that amine donors had relatively less influence on the stereoselectivity of IRED-catalyzed reductive aminations. In terms of conversion, five chain amines (a–e) are efficiently processed to create the corresponding amine products 1a–1d in good to excellent conversions (76–99%). An obvious preference for cyclopropanamine f among the four cyclic amines f–i was found in the reactions, and the conversions were up to 90% and 99%, respectively. Sterically hindered amines g–i, which were poor amine donors for our previous report,^26^ showed low to moderate conversions (11–63%) in this study, and even a trace amount of 1i could be detected in the reactions. Additionally, the reactions between 1 and the amines j and k gave moderate to high conversions (29–95%), highlighting that the binding pockets of both variants are large enough for the bulky substrates.

### Synthetic application in the synthesis of leniolisib and JAK1 inhibitor intermediates

We then began to apply the IRED mutant to prepare pyrrolidinamine intermediates 2 and 3 of the potential drug molecules leniolisib and JAK1 inhibitor 4. Leniolisib is an orally active, potent PI3Kδ selective inhibitor with suitable properties and efficacy for clinical development as an anti-inflammatory and anti-neoplastic therapeutic.^40,44,45^ In 2018, Kim and co-workers reported 4 as a potential rheumatoid arthritis drug candidate. Its inhibitory activity and selectivity for JAK1 over other JAKs were based on replacing the piperidine moiety of tofacitinib with a pyrrolidine moiety.^47^

**Scheme 1:**
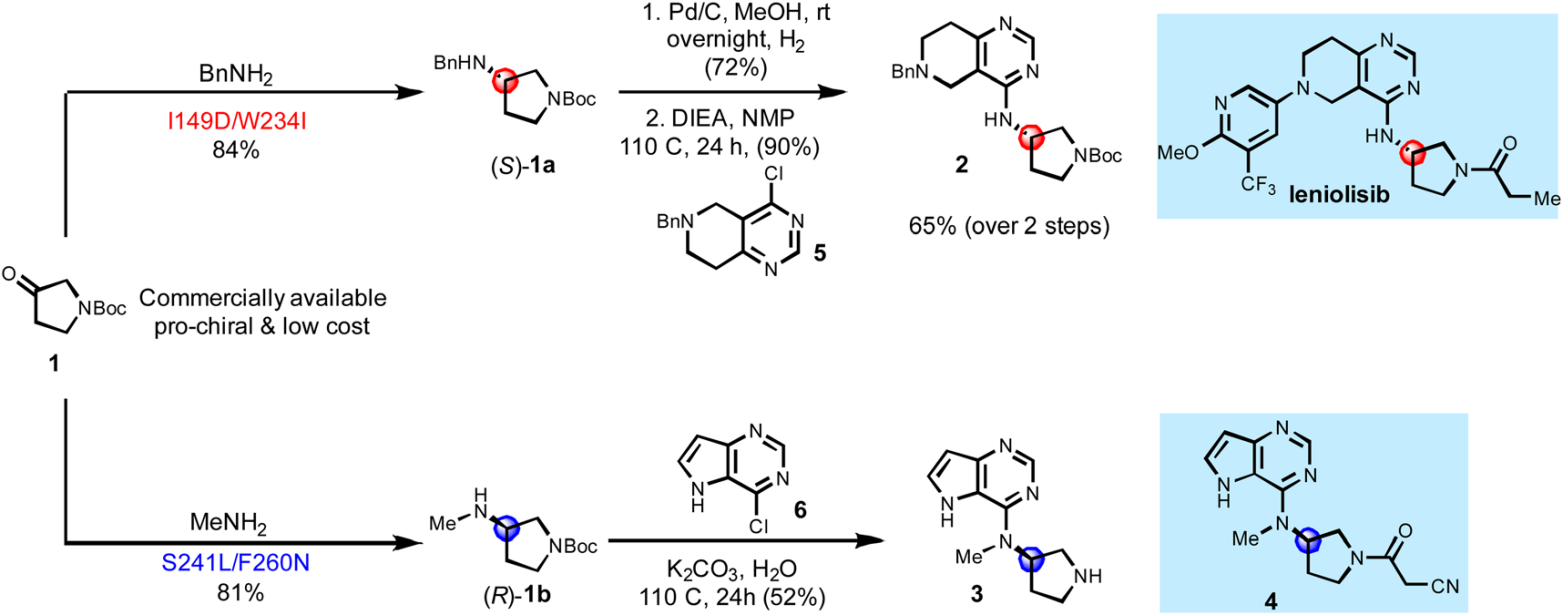
Stereo-divergent strategy for the synthesis of key intermediates 2 and 3 of leniolisib and JAK1 inhibitor 4.

The newly developed IRED catalyzed reductive amination enabled us to complete the synthesis of core skeleton 3 in two steps and 2 in three steps from pro-chiral compound 1, which are more efficient than the reported ones from pro-chiral compounds. Our synthesis started with cheaper and commercially available *N*-Boc-piperidone 1, which underwent asymmetric reductive amination catalyzed by the I149D/W234I mutant to give (*S*)-3-pyrrolidinamine 1a in 84% (99% ee) yield (Scheme 1). The benzyl protecting group was removed under the conditions of Pd/C with H_2_ in MeOH to obtain free amine in 72% yield. The S_N_Ar reaction with pyrimidine 5 was performed to produce the core skeleton 2 of leniolisib in 90% yield. Alternatively, treatment of *N*-Boc-piperidone 1 with mutant S241L/F260N successfully synthesized pyrrolidinamine 1b in 81% yield with *R*-configuration in 99% ee. The reaction with 6-chloro-7-deazapurine 6 under the conditions of K_2_CO_3_ in H_2_O reflux for 24 h gave the key intermediate 3 in 52% yield. Notably, in the S_N_Ar reaction step, the Boc protecting group was also removed under basic conditions.

## Conclusion

In conclusion, we constructed a biocatalytic platform for the enantio-complementary synthesis of pyrrolidinamines *via* engineered IRED-catalyzed reductive amination. Based on mechanism- and structure-guided semi-rational design, we demonstrate that amino acids interacting directly with the ketone moiety are hot spots for stereoselective modification. Two key sites 234 and 260 were recognized as the crucial “switches” which control the imine intermediate with Pro-(*S*) or Pro-(*R*) conformers in the active site. Screening of <80 mutants resulted in two combinatorial variants S241L/F260N and I149D/W234I with enhanced and reversed stereoselectivity with ee values up to >99% (*R*) and >99% (*S*), respectively. In addition, both variants showed high conversion and stereoselectivity for a series of pyrrolidinamines with various volumes, demonstrating the effectiveness of our strategy. Its synthetic potential was demonstrated by a 2 or 3-step synthesis of key intermediates of leniolisib and JAK1 inhibitor 4, respectively. We expect this new methodology to have broad application for the stereoselective engineering of IREDs to develop more biocatalytic platforms for the synthesis of chiral amines.

## Data availability

All the data supporting this article have been included in the main text and the ESI.†

## Author contributions

J. Z., Y. M., F. Z. and J. B. performed and analyzed the experiments. J. Z., S. G. and C. C. conceived the project and designed the experiments. J. Z., Q. W., S. G. and C. C. wrote the manuscript. All the authors discussed the results and commented on the manuscript.

## Conflicts of interest

There are no conflicts to declare.

## Supplementary Material

SC-014-D2SC07014F-s001
