# Supplementary material for: Structure-guided semi-rational design of an imine reductase for enantio-complementary synthesis of pyrrolidinamine
Source: Chem Sci. 2023 Mar 23;14(16):4265–72. doi: 10.1039/d2sc07014f (PMC10132124; doi:10.1039/d2sc07014f)
Supplement: SC-014-D2SC07014F-s001 [file SC-014-D2SC07014F-s001.pdf]

Electronic Supplementary Material (ESI) for Chemical Science.
This journal is © The Royal Society of Chemistry 2023

# Structure-guide rational design of an imine reductase for enantio-complementary synthesis of pyrrolidinamine

Jun Zhang,‡[ab] Yaqing Ma,‡[ac] Fangfang Zhu,‡[ad] Jinping Bao,[a] Qiaqing Wu,[ae] Shu-Shan Gao,*[ae] Chengsen Cui*[ae]

[a]Tianjin Institute of Industrial Biotechnology, Chinese Academy of Sciences, Tianjin 300308, China

[b]School of Life Science, Hebei University, Baoding 071002, China

[c]CAS Key Laboratory of Microbial Physiological and Metabolic Engineering, State Key Laboratory of Microbial Resources, Institute of Microbiology, Chinese Academy of Sciences, Beijing 100101, China

[d]College of Biotechnology, Tianjin University of Science and Technology, Tianjin 300457, China

[e]National Technology Innovation Center of Synthetic Biology, Tianjin 300308, China

‡ These authors contributed equally to this work.

## Table of Contents

1. General ........................................ 3

2. Materials ........................................ 3

3. Preparation of standard amines ........................................ 3

   3.1. Reductive amination procedure for the preparation of racemic amine standards ........ 3
   3.2. Reductive amination procedure for the preparation of chiral amine standards ........ 3
   3.3. NMR data of amine products ........................................ 4
   3.4. Synthetic application ........................................ 6

4. Site-saturation mutagenesis library construction and enantioselectivity analysis ........ 7

5. Triple code saturation mutagenesis (A/L/F) library construction and enantioselectivity analysis 8

6. Expression and purification of IR-G36-M5 ........................................ 8

7. Enzymic reductive aminations ........................................ 8

8. Biotransformation using cell-free extract ........................................ 9

9. HPLC and chiral HPLC analysis ........................................ 9

10. Molecular docking ........................................ 9

11. Sequence of IR-G36-M5 and the virants ........................................ 10

Chiral HPLC Chromatograms ........................................ 15

NMR spectra of amine products ........................................ 23

HR-ESI-MS spectra of amine products ........................................ 38

References ........................................ 41

# 1. General

$^1$H NMR spectra were recorded in $CDCl_3$, $(CD_3)_2SO$ (400 or 500 MHz). Residual solvent peaks are used as the internal reference; the signals at 7.26 ppm are set for $_1$H NMR spectra, taken in $CDCl_3$. Silica gel plates pre-coated on glass were used for thin-layer chromatography using UV light, or 7% ethanolic phosphomolybdic acid or potassium permanganate solution and heating as the visualizing methods. Silica gel was used for flash column chromatography with mixed $CH_2Cl_2$ and MeOH or ethyl acetate (EtOAc) and hexane as the eluting solvents. HPLC analysis was performed on Waters 2695 using C18 analytical column (Agilent Eclipse XDB-C18, 4.6×250 mm, 5 μm). LC-MS analysis was performed on Agilent 1100 with a mass spectrum detector (MSD) using an analytical column (Ultimate XB-C18, 2.1×100 mm, 3.0 μm). Chiral HPLC analysis was performed on Waters 2695 using chiral analytical columns (CHIRALPAK AD/AY-H, etc., 4.6×250 mm, 5 μm). NMR spectra were recorded on a Bruker Avance 500 MHz spectrometer.

# 2. Materials

Commercially available chemicals and reagents, including ketones **1**, amines **a-m**, and $NADP^+$, were purchased from Meryer (Shanghai, China), Macklin (Shanghai, China), J&K Scientific (Beijing, China), or Sigma-Aldrich (Poole, Dorset, UK) unless stated otherwise. All HPLC or LC-MS grade solvents, including acetonitrile, n-hexane, and ethanol, were purchased from Thermo Fisher Scientific (Waltham, Massachusetts, USA). Sodium phosphate buffer (0.1 M, pH 7.0) was prepared in-house.

# 3. Preparation of standard amines

## 3.1. Reductive amination procedure for the preparation of racemic amine standards

A dry methanol (10 mL) solution of the cyclic ketone (2.0 mmol), the corresponding amine (3.0 mmol), and acetic acid (300 μL) under an $N_2$ atm was stirred for 1.5 h at r.t. The reaction was placed in an ice-$H_2O$ bath and sodium cyanoborohydride (0.377 g, 6.0 mmol) was added, the reaction mixture gradually warmed to r.t. and stirred overnight. The reaction progress was monitored by TLC and following completion was quenched with sat. $NaHCO_3$ solution (10 mL) and stirred for an additional 30 minutes. The mixture was extracted with EtOAc (3 × 10 mL) and the combined organic phase dried over $Na_2SO_4$ and concentrated under reduced pressure. Column chromatography on silica affords the corresponding racemic amine.

## 3.2. Reductive amination procedure for the preparation of chiral amine standards

A dry $CH_2Cl_2$ (10 mL) solution of the chiral amine (2.0 mmol), the corresponding aldehyde or ketone (2.0 mmol), and acetic acid (300 μL) under an $N_2$ atm was stirred for 1.5 h at r.t. The reaction was placed in an ice-$H_2O$ bath and $NaBH(OAc)_3$ (0.848 g, 4.0 mmol) were added, the reaction mixture gradually warmed to r.t. and stirred overnight. The reaction progress was monitored by TLC and following completion was quenched with sat. $NaHCO_3$ solution (10 mL) and stirred for an additional 30 minutes. The mixture was extracted with EtOAc (3 × 10 mL) and the combined organic phase dried over $Na_2SO_4$ and concentrated under reduced pressure. Column chromatography on silica afford the corresponding chiral amine.

## 3.3. NMR data of amine products

3-benzylamino-1-boc-pyrrolidine, **1a**

Colorless oil. $^1$H NMR $\delta_H$ (500 MHz, $CD_3OD$) 7.40-7.48 (5H, overlap), 4.13 (2H, m), 3.75 (1H, m), 3.69 (1H, m), 3.54 (1H, m), 3.38 (m, 1H), 3.38 (2H, m), 2.31 (1H, m), 2.05 (1H, m), 1.46 (9H, s). $^{13}$C NMR $\delta_C$ (125 MHz, $CD_3OD$) 156.1, 136.3, 136.1, 130.3, 130.0, 129.6, 81.3, 58.0, 57.2, 52.1, 50.8, 50.3, 49.5, 49.3, 49.2, 49.0, 48.8, 48.7, 48.5, 45.3, 44.9, 30.7, 30.0, 28.7, 28.7.

3-methylamino-1-boc-pyrrolidine, **1b**

Colorless oil. $^1$H NMR $\delta_H$ (500 MHz, $CDCl_3$) 3.70 (1H, dd, $J$ = 11.6, 6.8 Hz), 3.38-3.55 (4H, overlap), 2.62 (3H, s), 2.25 (1H, m), 2.16 (1H, m), 1.45 (9H, s). $^{13}$C NMR $\delta_C$ (125 MHz, $CDCl_3$) 154.3, 80.2, 57.9, 57.4, 48.2, 48.1, 44.0, 43.8, 31.7, 28.8, 28.6, 28.1.

3-propargylamino-1-boc-pyrrolidine, **1c**

Brown oil. $^1$H NMR $\delta_H$ (600 MHz, $CD_3OD$) 3.55-3.60 (2H, overlap), 3.49 (2H, s), 3.45 (1H, m), 3.34 (1H, m), 3.18 (1H, m), 2.70 (1H, t, $J$ = 2.5 Hz), 2.13 (1H, m), 1.82 (1H, m), 1.46 (9H, s). $^{13}$C NMR $\delta_C$ (150 MHz, $CD_3OD$) 156.4, 81.0, 80.9, 74.2, 74.1, 64.4, 57.4, 56.6, 51.9, 51.4, 45.5, 45.1, 36.8, 31.6, 30.1, 28.7.

3-propylamino-1-boc-pyrrolidine, **1d**

Colorless oil. $^1$H NMR $\delta_H$ (500 MHz, $CDCl_3$) 3.36-3.72 (5H, overlap), 2.89 (2H, m), 2.15-2.27 (2H, m), 1.76 (2H, m), 1.45 (9H, s), 0.99 (3H, t, $J$ = 7.1 Hz). $^{13}$C NMR $\delta_C$ (125 MHz, $CDCl_3$) 154.09, 79.9, 56.4, 55.8, 48.3, 47.9, 44.1, 43.9, 28.5, 27.9, 19.9, 11.4.

3-amylamino-1-boc-pyrrolidine, **1e**

Colorless oil. $^1$H NMR $\delta_H$ (500 MHz, $CDCl_3$) 3.73 (1H, m), 3.56 (2H, m), 3.33-3.45 (2H, m), 2.83 (2H, m), 2.24 (1H, m), 2.10-2.17 (1H, m), 1.70 (2H, m), 1.44 (9H, s), 1.31 (4H, overlap), 0.88 (3H, t, $J$ = 6.8 Hz). $^{13}$C NMR $\delta_C$ (125 MHz, $CDCl_3$) 154.2, 80.1, 80.0, 56.5, 55.9, 48.2, 48.1, 47.0, 44.1, 43.9, 29.1, 29.0, 28.6, 28.3, 26.4, 26.3, 22.3, 14.0.

3-cyclopropylamino-1-boc-pyrrolidine, **1f**

Colorless oil. $^1$H NMR $\delta_H$ (500 MHz, $CDCl_3$) 3.59 (1H, m), 3.43 (2H, m), 3.32 (1H, m), 3.14 (1H, m), 2.54 (1H, m), 2.07-2.15 (2H, m), 1.78 (1H, m), 1.45 (9H, s), 0.40-0.49 (3H, overlap). $^{13}$C NMR $\delta_C$ (125

MHz, $CDCl_3$) 154.78, 79.32, 77.41, 77.36, 77.16, 76.91, 58.07, 57.39, 51.80, 51.38, 44.51, 44.13, 31.99, 31.27, 29.26, 29.11, 28.67, 28.63, 6.35.

3-cyclobutylamino-1-boc-pyrrolidine, **1g**

Colorless oil. $^1H$ NMR $\delta_H$ (500 MHz, $CDCl_3$) 3.66 (1H, m), 3.48-3.56 (3H, overlap), 3.33 (2H, m), 2.25 (5H, overlap), 2.07 (1H, m), 1.87 (1H, m), 1.79 (1H, m), 1.44 (9H, s). $^{13}C$ NMR $\delta_C$ (125 MHz, $CDCl_3$) 154.3, 80.0, 80.0, 54.8, 54.3, 51.6, 48.8, 48.6, 44.1, 43.8, 29.7, 28.9, 28.6, 28.2, 28.1, 28.0, 15.4.

3-cyclopentylamino-1-boc-pyrrolidine, **1h**

Colorless oil. $^1H$ NMR $\delta_H$ (500 MHz, $CDCl_3$) 3.76 (1H, m), 3.57 (2H, m), 3.31-3.43 (3H, overlap), 2.26 (1H, m), 2.02-2.17 (3H, overlap), 1.73-1.80 (4H, overlap), 1.58 (2H, m), 1.44 (9H, s). $^{13}C$ NMR $\delta_C$ (125 MHz, $CDCl_3$) 154.2, 80.0, 79.9, 58.3, 55.4, 54.9, 48.5, 48.3, 44.2, 43.9, 30.6, 30.5, 30.3, 30.2, 29.5, 28.6, 28.5, 24.0.

3-cyclohexylamino-1-boc-pyrrolidine, **1i**

Colorless oil. $^1H$ NMR $\delta_H$ (500 MHz, $CDCl_3$) 3.67-3.78 (2H, overlap), 3.56 (1H, m), 3.40 (1H, m), 3.30 (1H, m), 2.84 (1H, m), 2.09-2.25 (4H, overlap), 1.83 (2H, m), 1.66 (1H, m), 1.44 (9H, s), 1.42 (1H, m), 1.24 (3H, overlap). $^{13}C$ NMR $\delta_C$ (125 MHz, $CDCl_3$) 154.2, 80.1, 80.0, 56.1, 53.0, 52.4, 48.3, 48.1, 44.1, 43.8, 30.4, 30.2, 30.0, 29.6, 29.3, 28.6, 28.5, 25.2, 24.8.

3-(2-thiophene ethylamino)-1-boc-pyrrolidine, **1j**

Yellow oil. $^1H$ NMR $\delta_H$ (500 MHz, $CDCl_3$) 7.17 (1H, d, $J$ = 5.0 Hz), 6.94 (1H, dd, $J$ = 5.0, 3.4 Hz), 6.87 (1H, d, $J$ = 3.4 Hz), 3.65 (1H, m), 3.49 (2H, m), 3.24-3.34 (2H, overlap), 3.17 (2H, t, $J$ = 7.2 Hz), 3.06 (2H, m), 2.17 (1H, m), 1.95 (1H, m), 1.44 (9H, s). $^{13}C$ NMR $\delta_C$ (125 MHz, $CDCl_3$) 154.5, 140.3, 127.3, 125.9, 124.4, 79.9, 57.2, 56.5, 50.1, 49.7, 48.7, 44.3, 44.0, 30.5, 29.8, 28.8, 28.7, 28.6.

3-phenethylamino-1-boc-pyrrolidine, **1k**

Colorless oil. $^1H$ NMR $\delta_H$ (500 MHz, $CDCl_3$) 7.30 (2H, overlap), 7.20-7.25 (3H, overlap), 3.68 (1H, m), 3.55 (2H, m), 3.33-3.40 (2H, m), 2.98-3.06 (4H, overlap), 2.20 (1H, m), 2.03 (1H, m), 1.43 (9H, s). $^{13}C$ NMR $\delta_C$ (125 MHz, $CDCl_3$) 154.3, 137.6, 128.9, 128.8, 127.1, 80.0, 77.4, 77.2, 76.9, 57.0, 56.3, 49.3, 49.0, 48.5, 44.2, 43.9, 33.9, 33.8, 29.9, 29.1, 28.6.

3-phenylpropylamino-1-boc-pyrrolidine, **1l**

Colorless oil. $^1H$ NMR $\delta_H$ (500 MHz, $CDCl_3$) 7.26 (2H, overlap), 7.18 (1H, t, $J$ = 7.3 Hz), 7.13 (2H, d, overlap), 3.66 (1H, m), 3.27-3.51 (4H, overlap), 2.82 (2H, $J$ = 7.3 Hz), 2.63 (2H, t, $J$ = 7.3 Hz), 2.16 (1H, m), 2.08 (1H, m), 2.01 (2H, m), 1.43 (9H, s). $^{13}C$ NMR $\delta_C$ (125 MHz, $CDCl_3$) 154.2, 140.2, 128.7, 128.4, 128.4, 126.5, 80.1, 77.4, 77.2, 76.9, 56.7, 56.1, 48.3, 48.2, 46.4, 44.0, 43.8, 32.9, 29.2, 28.5, 28.2.

3-[2-(indol-3-yl)ethylamino]-1-boc-pyrrolidine, **1m**

Yellow oil. $^1H$ NMR $\delta_H$ (500 MHz, $CDCl_3$) 7.55 (1H, m), 7.35 (1H, d, $J$ = 8.0 Hz), 7.16 (1H, m), 7.03-7.08 (2H, overlap), 3.67 (1H, m), 3.46-3.51 (2H, m), 3.28-3.39 (2H, overlap), 3.15 (4H, overlap), 2.14

(1H, m), 1.89-2.07 (1H, m), 1.43 (9H, s). $^{13}C$ NMR $\delta_C$ (125 MHz, $CDCl_3$) 154.4, 136.6, 126.9, 123.1, 122.3, 119.7, 118.4, 111.6, 110.9, 110.7, 80.1, 56.9, 56.1, 49.0, 48.7, 47.1, 44.2, 43.9, 29.3, 28.8, 28.6, 23.5, 23.3.

## 3.4. Synthetic application

(*S*)-(−)-1-Boc-3-aminopyrrolidine, **1a-1**

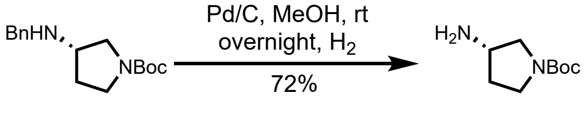


(*S*)-**1a** **1a-1**

To a stirred solution of (*S*)-**1a** (100 mg, 0.36 mmol) in methanol (5.0 ml) was added Pd/C (18 mg) at room temperature in a sealed tube. The flask was then purged with hydrogen gas, and the resulting mixture was stirred for 10 h at the same temperature. The reaction mixture was filtered through a pad of Celite with aid of methanol. The solvent was removed under reduced pressure, and the crude residue (48 mg, 72%) was used for the next step without further purification.

(*S*)-3-(6-Benzyl-5,6,7,8-tetrahydro-pyrido[4,3-d]pyrimidin-4-ylamino)-pyrrolidine-1-carboxylic acid tert-butyl ester, **2**

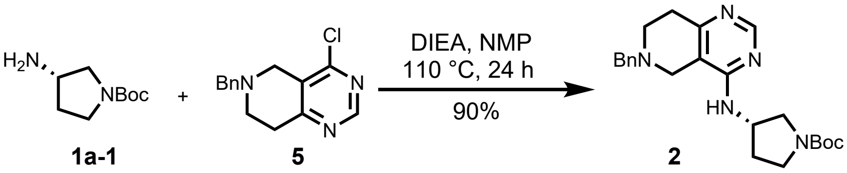


**1a-1** **5** **2**

To a stirred solution of amine **1a-1** (560 mg, 3.0 mmol) and pyrimidine **5** (779.2 mg, 3.0 mmol) in *N*-Methyl-2-pyrrolidone (5 mL) was added diisopropylethylamine (0.5 mL, 3.6 mmol) at room temperature. The reaction mixture was heated at 110 °C and stirred at the same temperature for 24 h. The reaction mixture was cooled to room temperature, diluted with tert-butyl methyl ether (10 mL), and the resulting suspension was stirred for 10 min. The mixture was diluted with water (5 mL), and the organic layer was separated. The organic layer was washed with brine, dried over $Na_2SO_4$, and concentrated under reduced pressure. The crude residue was purified by column chromatography ($SiO_2$, 2% MeOH in $CH_2Cl_2$) to afford **2** (410 mg, 90%) as a pale yellow foam. $^1H$ NMR $\delta_H$ (400 MHz, $CDCl_3$) 8.46 (s, 1H), 7.38-7.26 (m, 5H), 4.71 (brs, 1H), 4.53 (d, $J$ = 6.0 Hz, 1H), 3.77-3.74 (m, 1H), 3.74 (s, 2H), 3.55-3.43 (m, 2H), 3.31 (s, 2H), 3.31-3.10 (m, 1H), 2.83-2.77 (m, 4H), 2.28-2.20 (m, 1H), 1.89 (brs, 1H), 1.47 (s, 9H). $^{13}C$ NMR $\delta_C$ (100 MHz, $CDCl_3$) 159.9, 158.1, 155.8, 154.6, 137.8, 128.8, 128.5, 127.4, 110.7, 79.5, 62.5, 51.9, 51.3, 50.7*, 50.2*, 49.7, 49.2, 44.2, 43.9*, 31.9, 31.2, 28.5 (Signals for the rotational isomer denoted with asterix). The NMR data are in accordance with the reported literature.[1]

(*R*)-*N*-methyl-*N*-(pyrrolidin-3-yl)-7H-pyrrolo[2,3-d]pyrimidin-4-amine, **3**

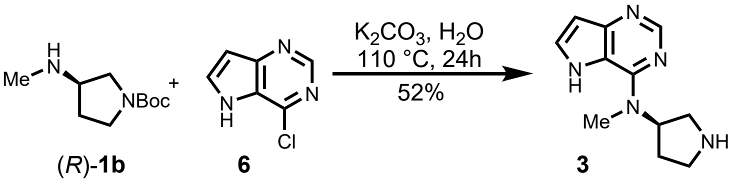


A solution of methylamine (*R*)-**1b** (390 mg, 1.95 mmol) in deionized water (10 mL) was placed in a 50 mL round bottom flask. Consequently, 6-chloro-7- deazapurine **6** (300 mg, 1.95 mmol) and potassium carbonate (539 mg, 3.90 mmol) were added, and the mixture was refluxed at 110 °C for 18 hours. After the reaction completed, it was cooled to room temperature and the aqueous mixture was extracted with $CH_2Cl_2$ (20x3 mL). The combined organic layers were dried over anhydrous $Na_2SO_4$, filtered, and concentrated under reduced pressure. The residue was purified with flash column chromatography ($SiO_2$, 2% MeOH in $CH_2Cl_2$) to afford product **3** (220 mg, 52%) as a yellow solid. $^1H$ NMR $\delta_H$ (400 MHz, $CDCl_3$) 10.1 (s, 1H), 8.38 (s, 1H), 7.29 (d, $J$ = 2.8 Hz, 1H), 6.50 (s, 1H), 4.07-3.99 (m, 2H), 3.95-3.90 (m, 1H), 3.70 (dd, $J$ = 10.2, 4.2 Hz, 1H), 3.40 (quint., $J$ = 5.0 Hz, 1H), 2.44 (s, 3H), 2.25-2.17 (m, 1H), 1.95-1.87 (m, 1H), 1.12 (brs, 1H). $^{13}C$ NMR $\delta_C$ (100 MHz, $CDCl_3$) 151.1, 149.4, 148.4, 127.6, 114.8, 102.9, 59.0, 53.3, 46.2, 34.9, 31.6. The NMR data are in accordance with the reported literature.[2]

## 4. Site-saturation mutagenesis library construction and enantioselectivity analysis

Site-saturation mutagenesis libraries were constructed using Fast Mutagenesis System (TransGen Biotech). The codon of the mutation site was replaced by the NNK degeneracy codon (Supplementary Table 1). *E. coli* BL21 (DE3) was transformed with pET28a-IR-G36-M5 generated by saturation mutagenesis. Colonies were picked up in deep-well plates containing 300 µL LB medium with 50 µg/mL kanamycin and cultured overnight at 37 °C with 800 rpm. The 19 diverse variants with the mutation site respectively replaced by the remaining AA residues were obtained through DNA sequencing. For proteins expression, pre-cultures of the 19 variants were grown in LB-medium (5 mL) containing 50 µg/mL kanamycin overnight at 37 °C with shaking at 220 rpm. An aliquot of 500 µL was transferred to a new culture flask containing 50 mL LB medium with 50 µg/mL kanamycin and cultured at 37 °C with shaking at 220 rpm to an OD600 of 0.6~0.8. Gene expression was induced by the addition of IPTG (0.1 mM) for 24 h at 20 °C, 220 rpm. The cells were harvested and washed twice with 100 mM pH 7.0 potassium phosphate buffer and centrifuged for 30 min with 4000 rpm. Then, the pellets were resuspended in 5 mL of the same buffer containing 6 U DNase I and 1 mg/mL lysozyme for breaking the cell at 30 °C, 200 rpm for 2 h. Then, 200 µL stock solution containing $NADP^+$ (1 mM), ketone 2 (30 mM, 1eq), amine a (33 mM, 1.1eq), *D*-glucose (50 mM), 1 mg/mL glucose dehydrogenase (GDH) and 20% v/v DMSO was added. After being shaken for 24 h at 30 °C with 200 rpm, reactions were quenched by the addition of 1 mL

saturated sodium carbonate and extracted with ethyl acetate (3×10 mL). The combined organic extracts were finally dried using anhydrous sodium sulfate, and the solvent was removed under reduced pressure. The extracts were dissolved in ethanol and subjected to HPLC analysis for measurement of the ee values.

## 5. Triple code saturation mutagenesis (A/L/F) library construction and enantioselectivity analysis

Site-saturation mutagenesis libraries were constructed using Fast Mutagenesis System (TransGen Biotech). The codon of the mutation site was replaced by the NNK degeneracy codon (Supplementary Table 1). Subsequent procedure of mutagenesis (A/L/F) library construction and enantioselectivity analysis is the same as the above method.

## 6. Expression and purification of IR-G36-M5

The plasmids containing the genes for target enzymes were used to transform *E. coli* BL21(DE3) competent cells for gene expression. Pre-cultures were grown in LB-medium (10 mL) containing 50 μg/mL kanamycin overnight at 37 °C with shaking at 220 rpm. 1L volume cultures were inoculated with the pre-culture (10 mL) and incubated at 37°C, with shaking at 220 rpm to an $OD_{600}$ of 0.6-0.8. Gene expression was induced by the addition of 0.1 mM isopropyl-*β*-*D*-thiogalactopyranoside (IPTG) and shaking was continued for 16 h at 18 °C, 180 rpm. The cells were then harvested by centrifugation at 5000 rpm for 30 min and resuspended in binding buffer (50 mM Tris-HCl buffer pH 8.0, 300 mM NaCl, containing 20 mM imidazole). Cells were disrupted by ultrasonication for 30 min, 5 s on, 9 s off cycles, and the suspension was centrifuged at 1,2000 rpm for 25 min to yield a clear lysate. The N-terminal His-tagged proteins were purified using the Ni-NTA column. In each case, the lysate was loaded onto a pre-equilibrated Ni-NTA column, followed by washing with 40~60 mL a washing buffer (50 mM Tris-HCl buffer pH 8.0, 300 mM NaCl, containing 40 mM imidazole). The bound protein was eluted with elution buffer containing 250 mM imidazole. Proteins were concentrated, and used for biotransformation reactions.

## 7. Enzymic reductive aminations

Purified proteins of the variants of IR-G36-M5 were used to measuring the substrate loading. A typical 5 mL reaction mixture contained 1 mM $NADP^+$, 5 mg/mL purified enzymes, 80-150 mM ketone **1**, amine **a** (1.1 eq), *D*-glucose (1.5 eq), 1 mg/mL GDH, 20% DMSO in sodium phosphate buffer (100 mM, pH 7.0). All reactions were incubated at 30 °C with shaking at 220 rpm for 24 h, after which they were quenched by the addition of 5 mL acetonitrile containing 1M acetic acid.

Then, the mixtures were centrifuged at 12000 rpm for 10 minutes, and the supernatant was subjected to HPLC analysis.

## 8. Biotransformation using cell-free extract

Cell-free extract of M5-S241L/F260 and M5-I149D/W234I were used to measuring the conversion and enantioselectivity for ketone **1** and amine **b-m**. A typical 50 mL reaction mixture contained 1 mM $NADP^+$, cell-free extract (10 g/L wet cells weight), 100 mM (for M5-S241L/F260 catalyzed reactions) or 110 mM (M5-I149D/W234I catalyzed reactions) ketone **1**, amine **b-m** (1.1 eq, stock amine solution was adjusted to pH 7.0 with 1 M HCl), *D*-glucose (1.5 eq), 1 mg/mL GDH, 20% DMSO in sodium phosphate buffer (100 mM, pH 7.0). After being shaken for 24 h at 30 °C with 200 rpm, the reactions were quenched with acetic acid to give a solution with pH 3.0, and celite was added. The mixtures were filtered and rinsed with water. The filtrate was extracted with dichloromethane (50 mL × 3) to remove the neutral materials. The aqueous phase was alkalified to pH 10 with saturated sodium carbonate, and then extracted with dichloromethane (50 mL × 3). The solvent of the combined organic extract was removed under reduced pressure to afford the corresponding amine products.

## 9. HPLC and chiral HPLC analysis

All biotransformation products were analyzed by HPLC and the products were confirmed by UV spectrum comparison with standards. Substrate loading and conversions were calculated according to the standard curves. Standard curves were plotted for varying concentrations of standards using the UV detection wavelength of 210 nm (**1a** and **1j-1m**) or 204 nm (**1b-1i**). The samples above were analyzed by Waters 2695 Separation Module and Wates 2996 Photodiode Array Detector with gradient method (1.0 mL/min, 20 min, $H_2O$/MeCN, 90/10→0/100, v/v) using C-18 analytic column (Phenomenex Gemini, 4.6×250 mm, 5 μm).

The ee values of racemic amine standards, chiral amine standards and biotransformation products for **1a-1m** were analyzed by chiral HPLC with different chiral columns and different solvent ratios of *n*-hexane and ethanol containing 0.02% diethylamine.

## 10. Molecular docking

The previously reported crystal structure of M5 (7WNW) which is a complex with $NADP^+$, is used as the receptor target in this study. The structures of M5-S241L/F260, and M5-I149D/W234I are generated by using the standard mutation protocol of Discovery Studio 2019 Client. Sites 149, 203, 259, and 260 were set as flexible residues using Discovery Studio 2019 Client. Flexible dockings

of imine intermediate of **1** and **a** into M5, M5- S241L/F260, and M5-I149D/W234I were performed using a flexible docking protocol. Docking runs were carried out using the standard parameters of the program. The conformations with the lowest energy were chosen for the analysis of substrate-enzyme interactions and distance between carbonyl group of the substrate and C4-H of NADPH.

## 11. Sequence of IR-G36-M5 and the virants

*DNA sequence of IR-G36-M5*

ATGGGCAGCAGCCATCATCATCATCATCACAGCAGCGGCCTGGTGCCGCGCGGCAGCCATATGCCGG
AATCTACCACCCCGAGTACCGCCACCCCGGTGACCATCATCGGTCTTGGTGCAATGGGCACCGCCCT
GGCAAACGCATTCCTCGATGCAGGTCATAGTACCACCGTTTGGAATCGTACCGCAGCACGCGCCACC
GCATTAGCCGCACGCGGCGCACATCATGCAGAAACCGTGACCGAAGCCATTGCAGCCTCTCCGTTAG
TGATTGCCTGTGTGCTGGATTATGATGCCTTTCATGAAACCTTAGCCCCGGCTACAGACGCGCTGGCA
GGTCGCGCCCTGGTTAATCTGACCACAGGTACCCCGAAACAGGCACGCGAAACCGCCTCTTGGGCAG
CCGATCATCGTATTGATTATCTGGATGGCAAAATTATGGCCATTCCGCCGGGTATTGCAACCCCGGAT
AGTTTTATTCTGTATAGCGGTCCGTTAGGTACCTTTGAAGCACATCGCTCAACCTTAGAAGTGCTGGG
CGCAGCAAATCATGTGGGTACCGATGCAGGTTTGGCGAGCTTACATGATATTGCACTGCTGACCGGT
ATGTATGGCATGATTGCAGGCATTTTACAGGCCTTTGCCTTAATTGATAGTGAAGGTATTCCGGCAGG
CGATCTGGCCCCGATGTTAACCAATTGGTTAACCGGCGCAGCACATAGCGTGGCCCATTATGCCCAG
CAGATTGATACCGGCGATTATGAAACCGGTGTTGTGTTTAATTTAGCACATCAGAGCCATGGCTTTGC
AAAATTAGTTCAGGCCGGTGAAGATCAGGGTGTGGATGTGGGCTTACTGCGTCCGCTGTTTGAACTG
ATGCGTCATCAGGTTGCCGCAGGCTATGGTAATGGTGATGTTGCCTCAGTTATTGAACTGATTCGTCG
CGAAGAACGTCGTCAGCCGGCCAAAAGTCCGGGCGCAGATAAAATTACCCGTGCACGTCGTCCGTA
A

*Amino acid sequence IR-G36-M5*

MGSSHHHHHHSSGLVPRGSHMPESTTPSTATPVTIIGLGAMGTALANAFLDAGHSTTVWNRTAARATAL
AARGAHHAETVTEAIAASPLVIACVLDYDAFHETLAPATDALAGRALVNLTTGTPKQARETASWAADHR
IDYLDGKIMAIPPGIATPDSFILYSGPLGTFEAHRSTLEVLGAANHVGTDAGLASLHDIALLTGMYGMIAGI
LQAFALIDSEGIPAGDLAPMLTNWLTGAAHSVAHYAQQIDTGDYETGVVFNLAHQSHGFAKLVQAGED
QGVDVGLLRPLFELMRHQVAAGYGNGDVASVIELIRREERRQPAKSPGADKITRARRP*

*DNA sequence of M5-S260N-S241L*

ATGGGCAGCAGCCATCATCATCATCATCACAGCAGCGGCCTGGTGCCGCGCGGCAGCCATATGCCGG
AATCTACCACCCCGAGTACCGCCACCCCGGTGACCATCATCGGTCTTGGTGCAATGGGCACCGCCCT
GGCAAACGCATTCCTCGATGCAGGTCATAGTACCACCGTTTGGAATCGTACCGCAGCACGCGCCACC

GCATTAGCCGCACGCGGCGCACATCATGCAGAAACCGTGACCGAAGCCATTGCAGCCTCTCCGTTAG

TGATTGCCTGTGTGCTGGATTATGATGCCTTTCATGAAACCTTAGCCCCGGCTACAGACGCGCTGGCA

GGTCGCGCCCTGGTTAATCTGACCACAGGTACCCCGAAACAGGCACGCGAAACCGCCTCTTGGGCAG

CCGATCATCGTATTGATTATCTGGATGGCAAAATTATGGCCATTCCGCCGGGTATTGCAACCCCGGAT

AGTTTTATTCTGTATAGCGGTCCGTTAGGTACCTTTGAAGCACATCGCTCAACCTTAGAAGTGCTGGG

CGCAGCAAATCATGTGGGTACCGATGCAGGTTTGGCGAGCTTACATGATATTGCACTGCTGACCGGT

ATGTATGGCATGATTGCAGGCATTTTACAGGCCTTTGCCTTAATTGATAGTGAAGGTATTCCGGCAGG

CGATCTGGCCCCGATGTTAACCAATTGGTTAACCGGCGCAGCACATTTAGTGGCCCATTATGCCCAG

CAGATTGATACCGGCGATTATGAAACCGGTGTTGTGAATAATTTAGCACATCAGAGCCATGGCTTTG

CAAAATTAGTTCAGGCCGGTGAAGATCAGGGTGTGGATGTGGGCTTACTGCGTCCGCTGTTTGAACT

GATGCGTCATCAGGTTGCCGCAGGCTATGGTAATGGTGATGTTGCCTCAGTTATTGAACTGATTCGTC

GCGAAGAACGTCGTCAGCCGGCCAAAAGTCCGGGCGCAGATAAAATTACCCGTGCACGTCGTCCGT

AA

*Amino acid sequence M5-S241L/F260*

MGSSHHHHHHSSGLVPRGSHMPESTTPSTATPVTIIGLGAMGTALANAFLDAGHSTTVWNRTAARATAL

AARGAHHAETVTEAIAASPLVIACVLDYDAFHETLAPATDALAGRALVNLTTGTPKQARETASWAADHR

IDYLDGKIMAIPPGIATPDSFILYSGPLGTFEAHRSTLEVLGAANHVGTDAGLASLHDIALLTGMYGMIAGI

LQAFALIDSEGIPAGDLAPMLTNWLTGAAHLVAHYAQQIDTGDYETGVVNNLAHQSHGFAKLVQAGED

QGVDVGLLRPLFELMRHQVAAGYGNGDVASVIELIRREERRQPAKSPGADKITRARRP*

*DNA sequence of M5-I149D/W234I*

ATGGGCAGCAGCCATCATCATCATCATCACAGCAGCGGCCTGGTGCCGCGCGGCAGCCATATGCCGG

AATCTACCACCCCGAGTACCGCCACCCCGGTGACCATCATCGGTCTTGGTGCAATGGGCACCGCCCT

GGCAAACGCATTCCTCGATGCAGGTCATAGTACCACCGTTTGGAATCGTACCGCAGCACGCGCCACC

GCATTAGCCGCACGCGGCGCACATCATGCAGAAACCGTGACCGAAGCCATTGCAGCCTCTCCGTTAG

TGATTGCCTGTGTGCTGGATTATGATGCCTTTCATGAAACCTTAGCCCCGGCTACAGACGCGCTGGCA

GGTCGCGCCCTGGTTAATCTGACCACAGGTACCCCGAAACAGGCACGCGAAACCGCCTCTTGGGCAG

CCGATCATCGTATTGATTATCTGGATGGCAAAATTATGGCCGATCCGCCGGGTATTGCAACCCCGGA

TAGTTTTATTCTGTATAGCGGTCCGTTAGGTACCTTTGAAGCACATCGCTCAACCTTAGAAGTGCTGG

GCGCAGCAAATCATGTGGGTACCGATGCAGGTTTGGCGAGCTTACATGATATTGCACTGCTGACCGG

TATGTATGGCATGATTGCAGGCATTTTACAGGCCTTTGCCTTAATTGATAGTGAAGGTATTCCGGCAG

GCGATCTGGCCCCGATGTTAACCAATATATTAACCGGCGCAGCACATAGCGTGGCCCATTATGCCCA

GCAGATTGATACCGGCGATTATGAAACCGGTGTTGTGTTTAATTTAGCACATCAGAGCCATGGCTTTG

CAAAATTAGTTCAGGCCGGTGAAGATCAGGGTGTGGATGTGGGCTTACTGCGTCCGCTGTTTGAACT

GATGCGTCATCAGGTTGCCGCAGGCTATGGTAATGGTGATGTTGCCTCAGTTATTGAACTGATTCGTC

GCGAAGAACGTCGTCAGCCGGCCAAAAGTCCGGGCGCAGATAAAATTACCCGTGCACGTCGTCCGT

AA

*Amino acid sequence of M5-I149D/W234I*

<u>MGSSHHHHHHSSGLVPRGSH</u>MPESTTPSTATPVTIIGLGAMGTALANAFLDAGHSTTVWNRTAARATAL

AARGAHHAETVTEAIAASPLVIACVLDYDAFHETLAPATDALAGRALVNLTTGTPKQARETASWAADHR

IDYLDGKIMADPPGIATPDSFILYSGPLGTFEAHRSTLEVLGAANHVGTDAGLASLHDIALLTGMYGMIAG

ILQAFALIDSEGIPAGDLAPMLTNILTGAAHSVAHYAQQIDTGDYETGVVFNLAHQSHGFAKLVQAGEDQ

GVDVGLLRPLFELMRHQVAAGYGNGDVASVIELIRREERRQPAKSPGADKITRARRP*

**Table 1.** List of primers in this study.

| Primer | mutant | Sequences (5′→3′) |
|---|---|---|
| F | F260NNK | GTTGTGNNKAATTTAGCACATCAGAG |
| R | F260NNK | CTAAATTMNNCACAACACCGGTTTCA |
| F | W234NNK | ACCAATNNKTTAACCGGCGCAGCACATAGC |
| R | W234NNK | GCCGGTTAAMNNATTGGTTAACATCGGGGC |
| F | S241A | AGCACATGCCGTGGCCCATTATGCCCA |
| R | S241A | GGGCCACGGCATGTGCTGCGCCGGTTAA |
| F | S241F | GCGCAGCACATTTTGTGGCCCATTATGCCCAGCAGATT |
| R | S241F | ATAATGGGCCACAAAATGTGCTGCGCCGGTTAACCAATT |
| F | S241L | AGCACATTTAGTGGCCCATTATGCCCAG |
| R | S241L | GGGCCACTAAATGTGCTGCGCCGGTTAA |
| F | M203A | ACCGGTGCATATGGCATGATTGCAGGCATT |
| R | M203A | GCCATATGCACCGGTCAGCAGTGCAATAT |
| F | M203F | ACCGGTTTTTATGGCATGATTGCAGGCATT |
| R | M203F | GCCATAAAAACCGGTCAGCAGTGCAATATC |
| F | M203L | ACCGGTTTATATGGCATGATTGCAGGCATT |
| R | M203L | GCCATATAAACCGGTCAGCAGTGCAATATC |
| F | I149A | TATGGCCGCACCGCCGGGTATTGCAACCC |
| R | I149A | CCCGGCGGTGCGGCCATAATTTTGCCATC |
| F | I149F | TATGGCCTTTCCGCCGGGTATTGCAACCC |
| R | I149F | CCCGGCGGAAAGGCCATAATTTTGCCATCC |
| F | I149L | TATGGCCTTACCGCCGGGTATTGCAACCC |
| R | I149L | CCCGGCGGTAAGGCCATAATTTTGCCATCC |
| F | I149NNK | TATGGCCNNKCCGCCGGGTATTGCAACCC |
| R | I149NNK | CCGGCGGMNNGGCCATAATTTTGCCATCC |

**Table 2.** Enantioselectivity of the mutants generated by site-saturation mutagenesis of residue F260 over M5.

| Mutants | ee value | Mutants | ee value | Mutants | ee value |
|---|---|---|---|---|---|
| F260H | 60%, *R* | F260M | 71%, *R* | F260N | 88%, *R* |
| F260R | N.D. | F260P | 73%, *R* | F260Q | 63%, *R* |
| F260K | N.D. | F260W | 45%, *R* | F260Y | 50%, *R* |
| F260A | 79%, *R* | F260G | 73%, *R* | F260D | 93%, *R* |
| F260L | 83%, *R* | F260C | 77%, *R* | F260E | 92%, *R* |
| F260I | 73%, *R* | F260S | 65%, *R* | | |
| F260V | 72%, *R* | F260T | 84%, *R* | | |

**Table 3.** Enantioselectivity of the mutants generated by site-saturation mutagenesis of residue W234 over M5.

| Mutants | ee value | Mutants | ee value | Mutants | ee value |
|---|---|---|---|---|---|
| W234H | 73%, *S* | W234M | 69%, *S* | W234N | 58%, *S* |
| W234R | 35%, *S* | W234P | 56%, *S* | W234Q | 44%, *S* |
| W234K | N.D. | W234F | 58%, *S* | W234Y | 49%, *S* |
| W234A | 39%, *S* | W234G | 57%, *S* | W234D | 63%, *S* |
| W234L | 81%, *S* | W234C | 25%, *S* | W234E | 71%, *S* |
| W234I | 82%, *S* | W234S | 52%, *S* | | |
| W234V | 58%, *S* | W234T | 56%, *S* | | |

**Table 4.** Enantioselectivity of the mutants generated by Triple code saturation mutagenesis (A/L/F) of residues S241, I149, and M203 over M5.

| Mutants | ee value | Mutants | ee value | Mutants | ee value |
|---|---|---|---|---|---|
| S241A | 53%, *R* | I149A | 45%, *S* | M203A | 65%, *R* |
| S241L | 85%, *R* | I149L | 79%, *R* | M203L | 25%, *R* |
| S241F | 51%, *R* | I149F | 84%, *R* | M203F | 38%, *R* |

**Table 5.** Enantioselectivity of the mutants generated by site-saturation mutagenesis of residue I149 over M5-W234I.

| Mutants | ee value | Mutants | ee value | Mutants | ee value |
|---|---|---|---|---|---|
| I149H | 99%, *S* | I149M | 76%, *S* | I149N | 93%, *S* |
| I149R | 95%, *S* | I149P | 95%, *S* | I149Q | 92%, *S* |
| I149K | 97%, *S* | I149W | 76%, *S* | I149Y | 24%, *S* |
| I149A | 97%, *S* | I149G | 96%, *S* | I149D | 99%, *S* |
| I149L | 49%, *S* | I149C | 94%, *S* | I149E | 94%, *S* |
| I149F | 48%, *R* | I149S | 96%, *S* | | |
| I149V | 86%, *S* | I149T | 90%, *S* | | |

## Chiral HPLC Chromatograms

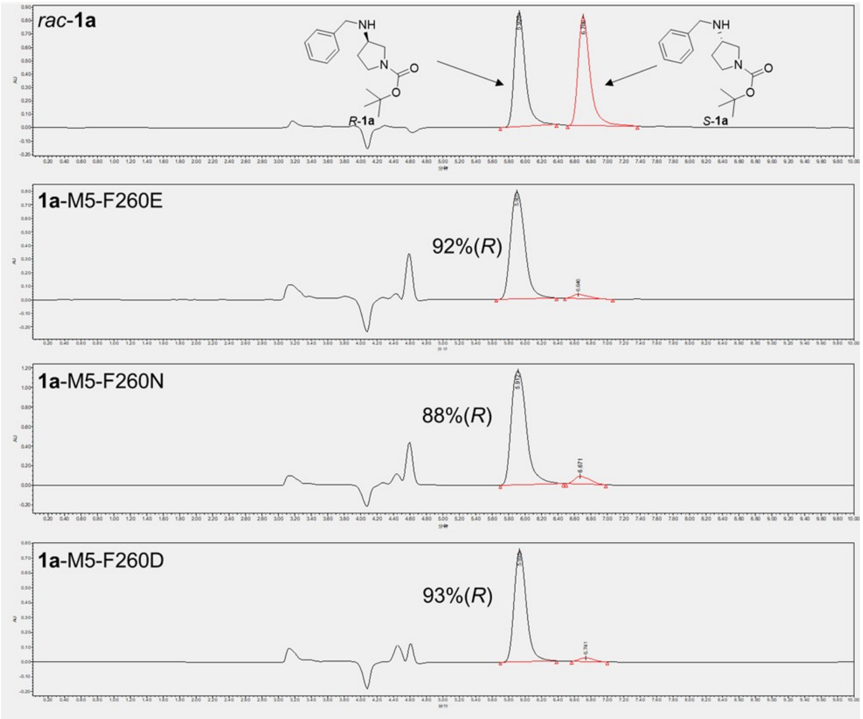


**Figure 1.** Chiral HPLC analysis of racemic standard of **1a**, and M5-F260E, M5-F260N, and M5-F260D catalytic product **1a**. HPLC conditions: CHIRALPAK AD-H column with a mobile phase of *n*-hexane/ethanol (90:10, v/v, 0.2‰ diethylamine), flow rate 1.0 mL/min, 30 °C, UV detection at 210 nm.

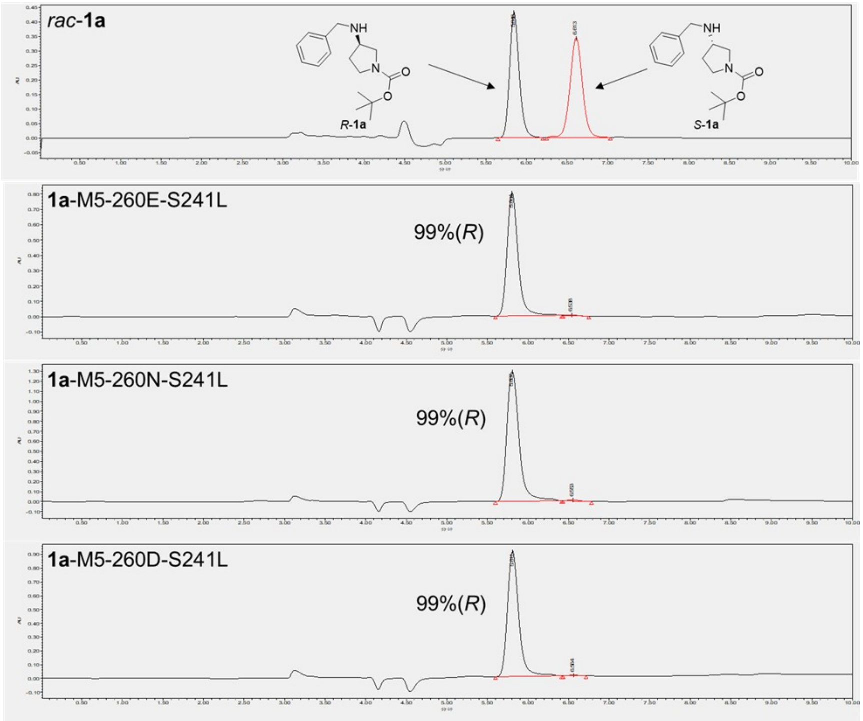


**Figure 2.** Chiral HPLC analysis of racemic standard of **1a**, and M5-F260E-S241L, M5-S241L/F260N, and M5-F260D-S241L catalytic product **1a**. HPLC conditions: CHIRALPAK AD-H

column with a mobile phase of *n*-hexane/ethanol (90:10, v/v, 0.2‰ diethylamine), flow rate 1.0 mL/min, 30 °C, UV detection at 210 nm.

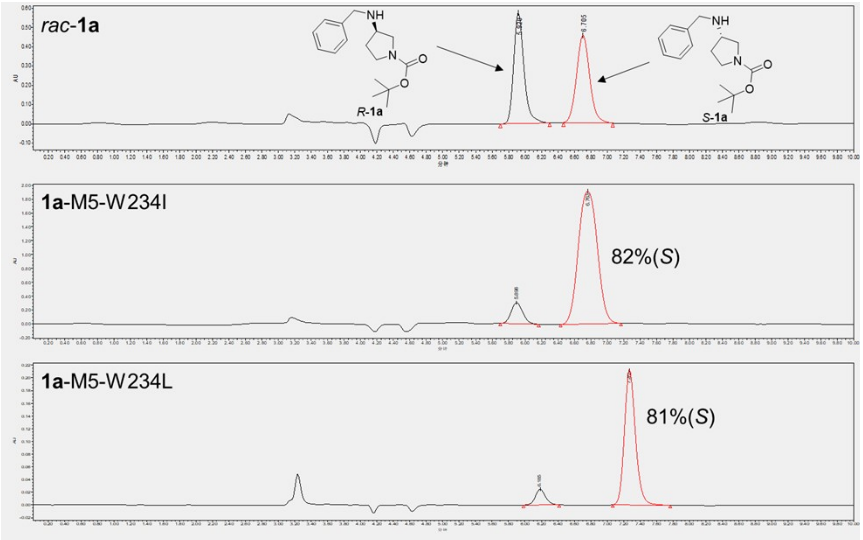


**Figure 3.** Chiral HPLC analysis of racemic standard of **1a**, and M5-W234I and M5-W234L catalytic product **1a**. HPLC conditions: CHIRALPAK AD-H column with a mobile phase of *n*-hexane/ethanol (90:10, v/v, 0.2‰ diethylamine), flow rate 1.0 mL/min, 30 °C, UV detection at 210 nm.

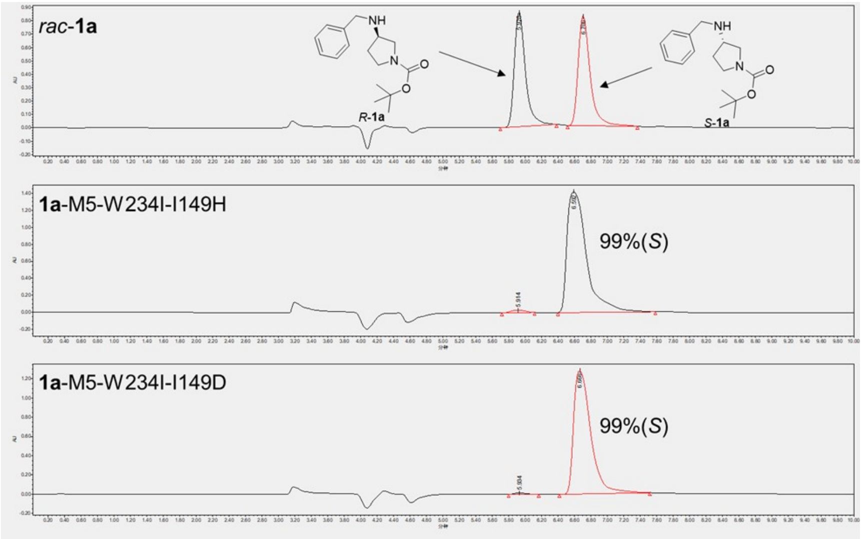


**Figure 4.** Chiral HPLC analysis of racemic standard of **1a**, and M5-W234I-I149H and M5-W234L-I149D catalytic product **1a**. HPLC conditions: CHIRALPAK AD-H column with a mobile phase of *n*-hexane/ethanol (90:10, v/v, 0.2‰ diethylamine), flow rate 1.0 mL/min, 30 °C, UV detection at 210 nm.

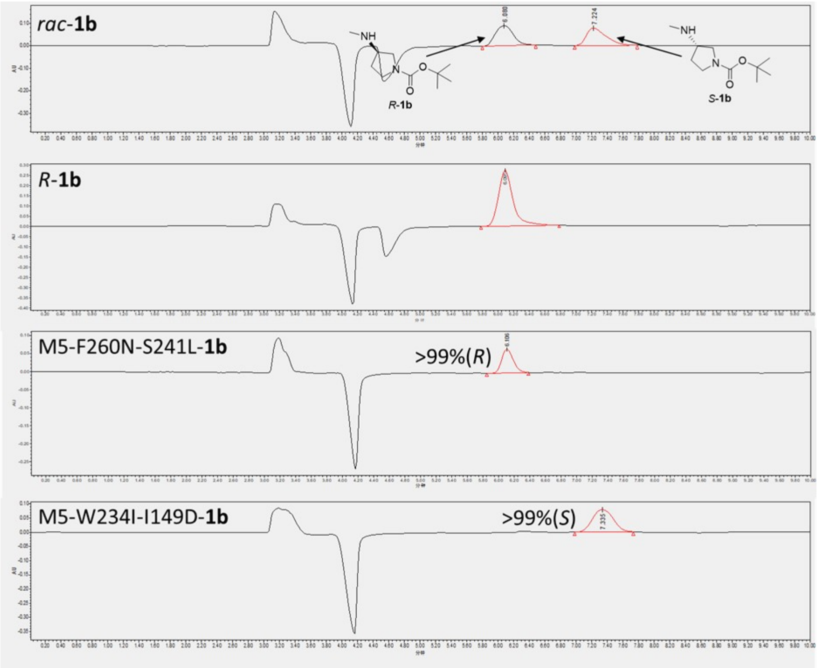


**Figure 5.** Chiral HPLC analysis of racemic standard of **1b**, optically pure standard of (*R*)-**1b**, M5-S241L/F260N catalytic product **1b** and M5-I149D/W234I catalytic product **1b**. HPLC conditions: CHIRALPAK AD-H column with a mobile phase of *n*-hexane/ethanol (90:10, v/v, 0.2‰ diethylamine), flow rate 1.0 mL/min, 30 °C, UV detection at 210 nm.

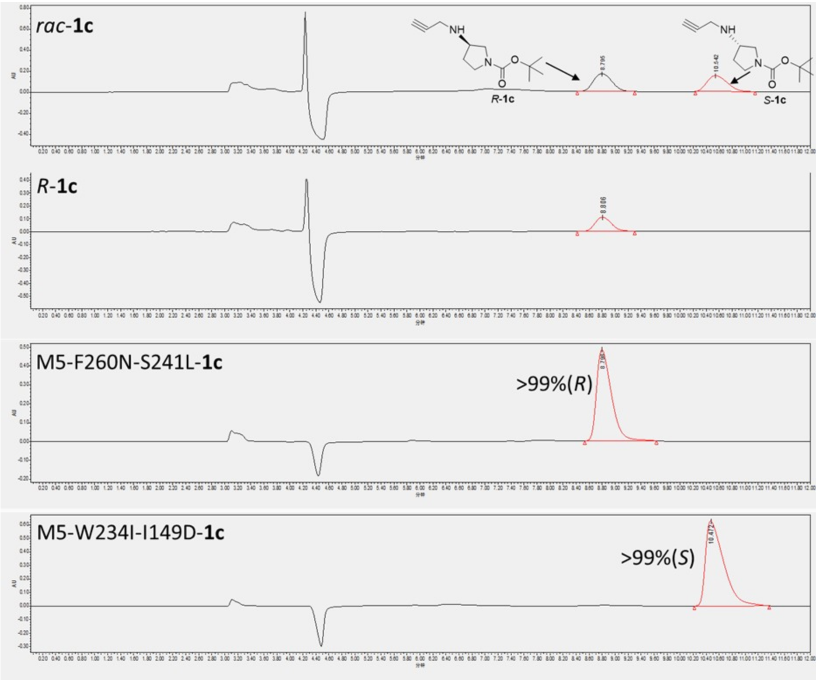


**Figure 6.** Chiral HPLC analysis of racemic standard of **1c**, optically pure standard of (*R*)-**1c**, M5-S241L/F260N catalytic product **1c** and M5-I149D/W234I catalytic product **1c**. HPLC conditions: CHIRALPAK IG column with a mobile phase of *n*-hexane/ethanol (85:15, v/v, 0.2‰ diethylamine), flow rate 1.0 mL/min, 30 °C, UV detection at 210 nm.

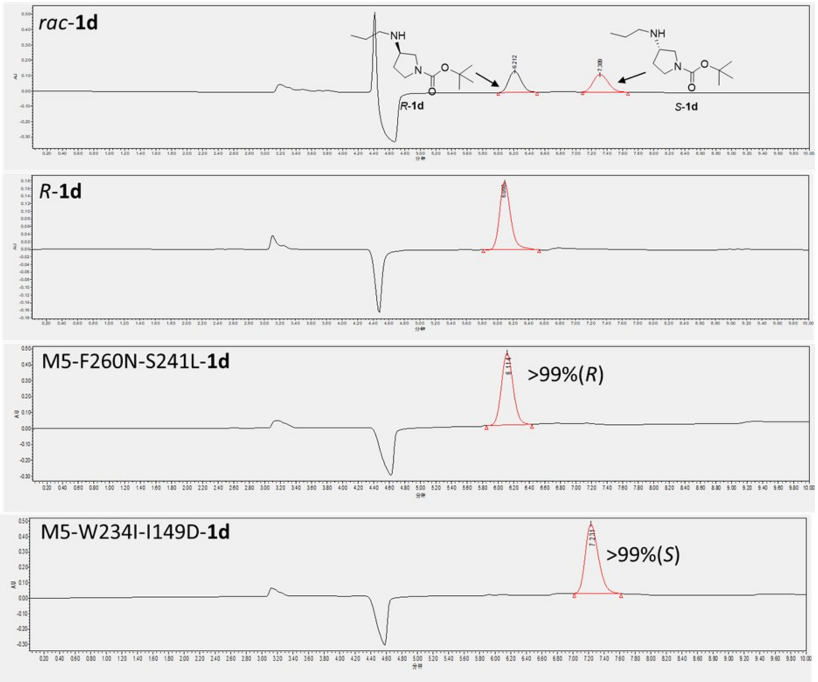


**Figure 7.** Chiral HPLC analysis of racemic standard of **1d**, optically pure standard of (*R*)-**1d**, M5-S241L/F260N catalytic product **1d** and M5-I149D/W234I catalytic product **1d**. HPLC conditions: CHIRALPAK IG column with a mobile phase of *n*-hexane/ethanol (85:15, v/v, 0.2‰ diethylamine), flow rate 1.0 mL/min, 30 °C, UV detection at 210 nm.

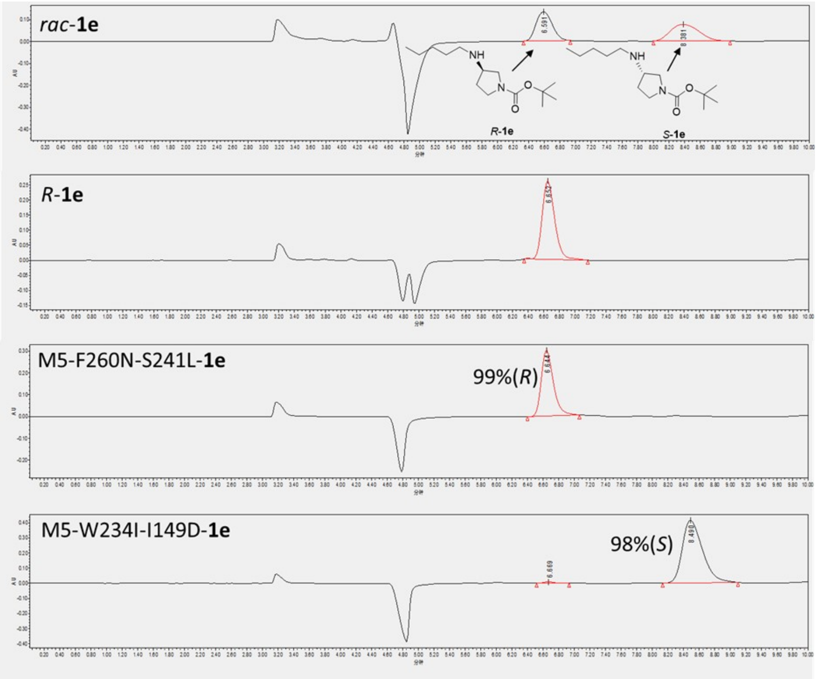


**Figure 8.** Chiral HPLC analysis of racemic standard of **1e**, optically pure standard of (*R*)-**1e**, M5-S241L/F260N catalytic product **1e** and M5-I149D/W234I catalytic product **1e**. HPLC conditions: CHIRALPAK IG column with a mobile phase of *n*-hexane/ethanol (90:10, v/v, 0.2‰ diethylamine), flow rate 1.0 mL/min, 30 °C, UV detection at 210 nm.

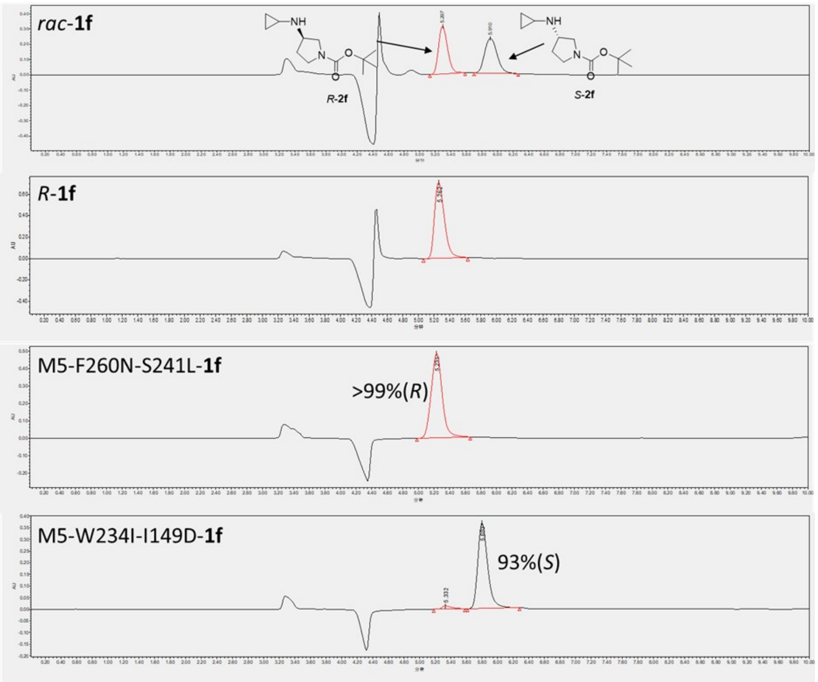


**Figure 9.** Chiral HPLC analysis of racemic standard of **1f**, optically pure standard of (*R*)-**1f**, M5-S241L/F260N catalytic product **1f** and M5-I149D/W234I catalytic product **1f**. HPLC conditions: CHIRALPAK AY-H column with a mobile phase of *n*-hexane/ethanol (90:10, v/v, 0.2‰ diethylamine), flow rate 1.0 mL/min, 30 °C, UV detection at 210 nm.

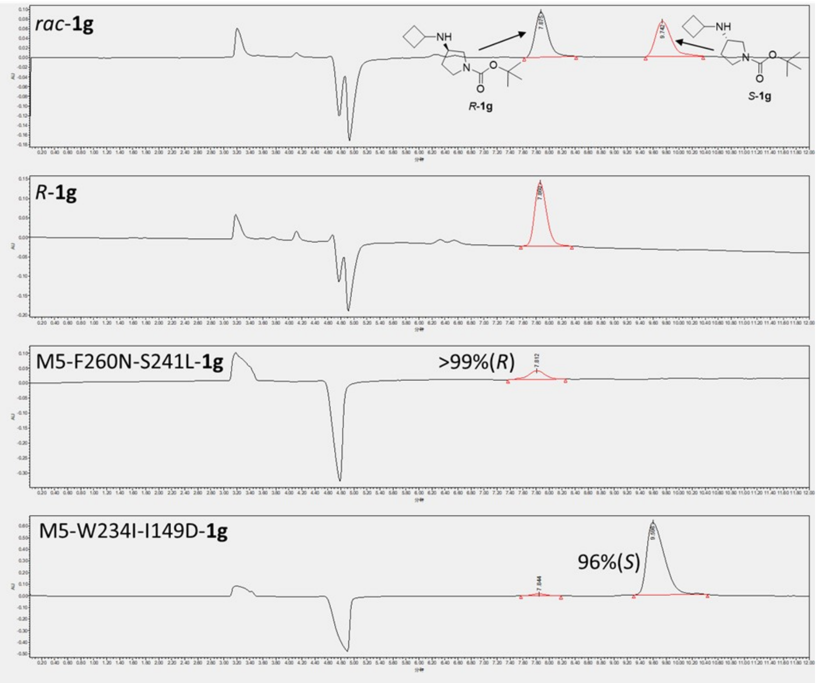


**Figure 10.** Chiral HPLC analysis of racemic standard of **1g**, optically pure standard of (*R*)-**1g**, M5-S241L/F260N catalytic product **1g** and M5-I149D/W234I catalytic product **1g**. HPLC conditions: CHIRALPAK IG column with a mobile phase of *n*-hexane/ethanol (90:10, v/v, 0.2‰ diethylamine), flow rate 1.0 mL/min, 30 °C, UV detection at 210 nm.

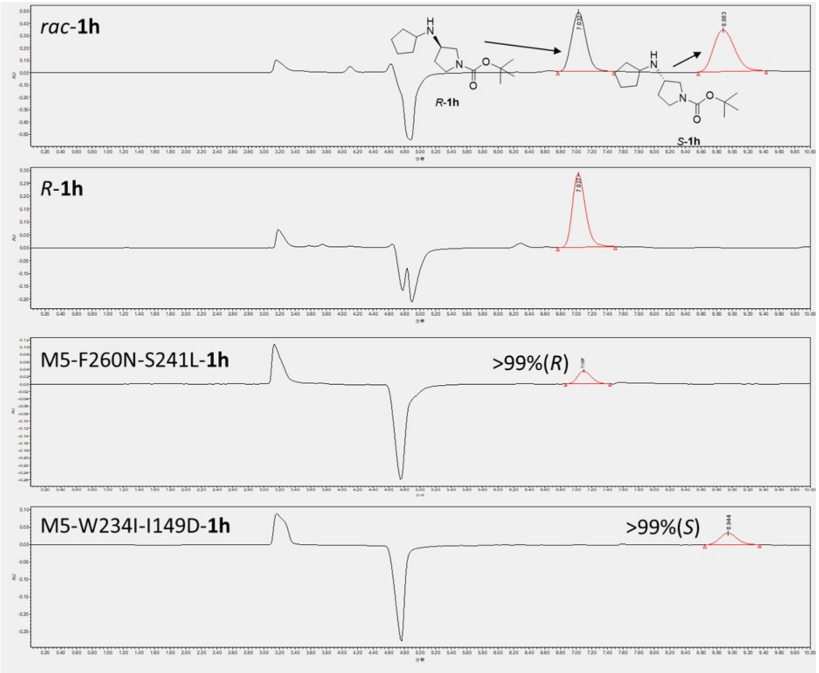


**Figure 11.** Chiral HPLC analysis of racemic standard of **1h**, optically pure standard of (*R*)-**1h**, M5-S241L/F260N catalytic product **1h** and M5-I149D/W234I catalytic product **1h**. HPLC conditions: CHIRALPAK IG column with a mobile phase of *n*-hexane/ethanol (90:10, v/v, 0.2‰ diethylamine), flow rate 1.0 mL/min, 30 °C, UV detection at 210 nm.

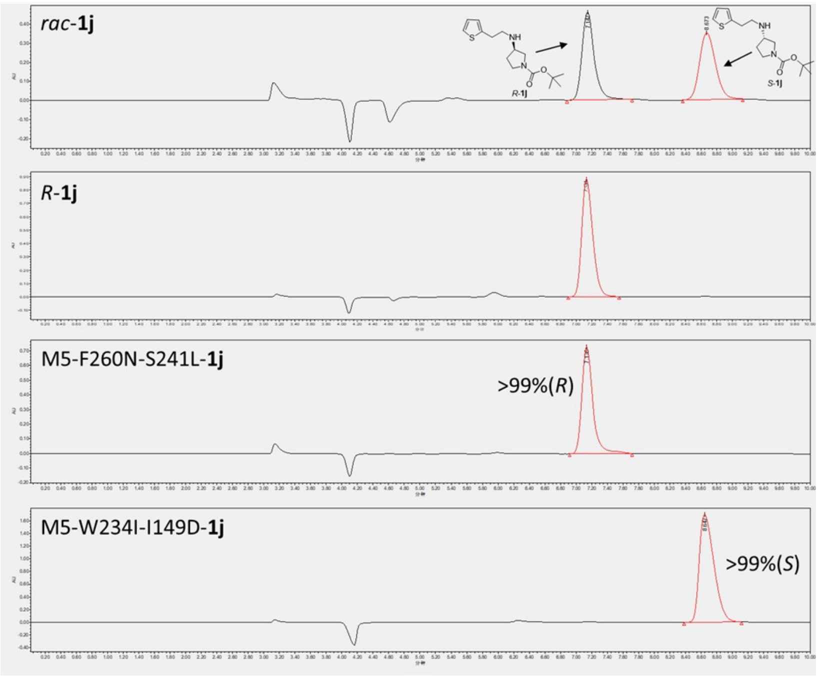


**Figure 12.** Chiral HPLC analysis of racemic standard of **1j**, optically pure standard of (*R*)-**1j**, M5-S241L/F260N catalytic product **1j** and M5-I149D/W234I catalytic product **1j**. HPLC conditions: CHIRALPAK AD-H column with a mobile phase of *n*-hexane/ethanol (90:10, v/v, 0.2‰ diethylamine), flow rate 1.0 mL/min, 30 °C, UV detection at 210 nm.

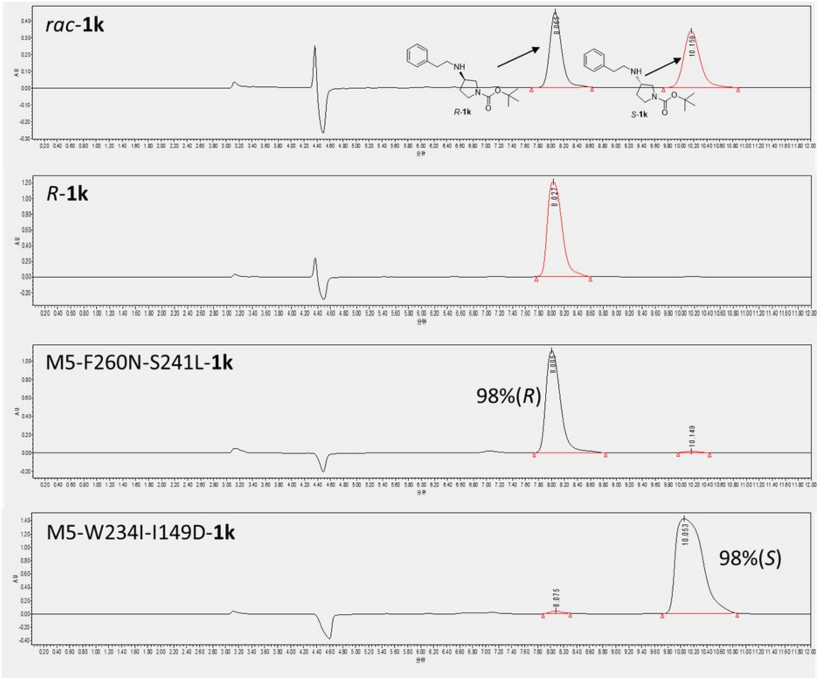


**Figure 13.** Chiral HPLC analysis of racemic standard of **1k**, optically pure standard of (*R*)-**1k**, M5-S241L/F260N catalytic product **1k** and M5-I149D/W234I catalytic product **1k**. HPLC conditions: CHIRALPAK IG column with a mobile phase of *n*-hexane/ethanol (85:15, v/v, 0.2‰ diethylamine), flow rate 1.0 mL/min, 30 °C, UV detection at 210 nm.

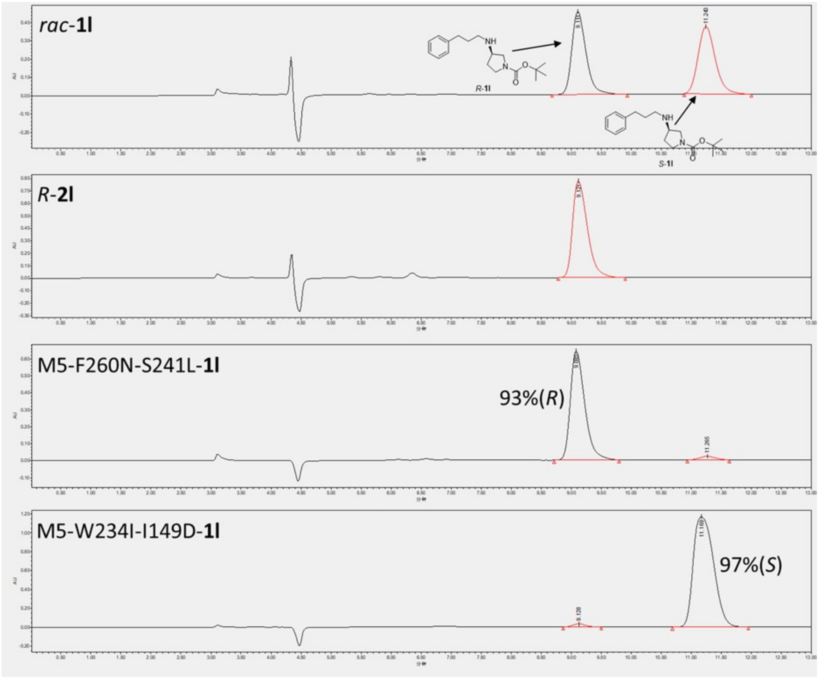


**Figure 14.** Chiral HPLC analysis of racemic standard of **1l**, optically pure standard of (*R*)-**1l**, M5-S241L/F260N catalytic product **1l** and M5-I149D/W234I catalytic product **1l**. HPLC conditions: CHIRALPAK IG column with a mobile phase of *n*-hexane/ethanol (85:15, v/v, 0.2‰ diethylamine), flow rate 1.0 mL/min, 30 °C, UV detection at 210 nm.

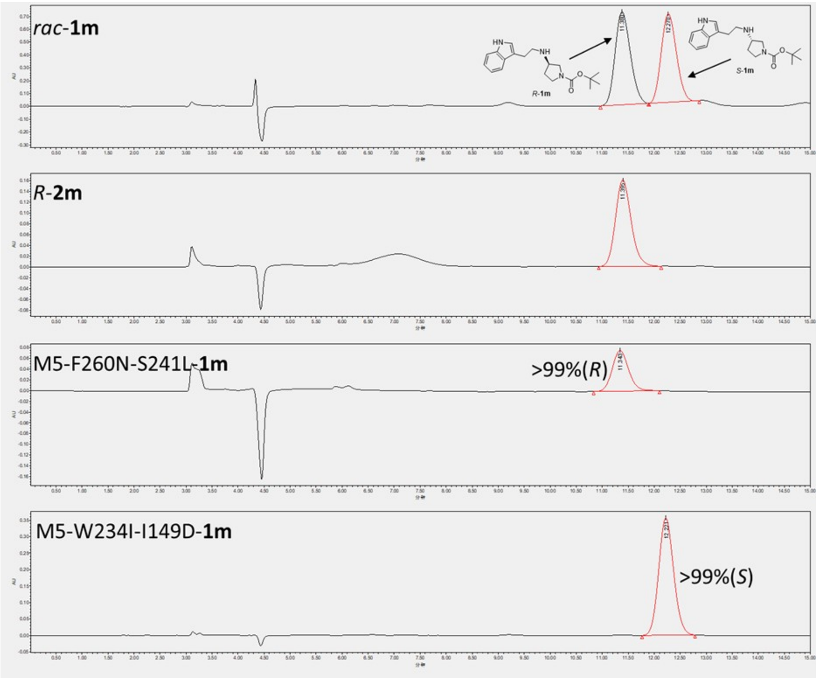


**Figure 15.** Chiral HPLC analysis of racemic standard of **1m**, optically pure standard of (*R*)-**1m**, M5-S241L/F260 catalytic product **1m** and M5-I149D/W234I catalytic product **1m** HPLC conditions: CHIRALPAK IG column with a mobile phase of *n*-hexane/ethanol (85:15, v/v, 0.2‰ diethylamine), flow rate 1.0 mL/min, 30 °C, UV detection at 210 nm.

## NMR spectra of amine products

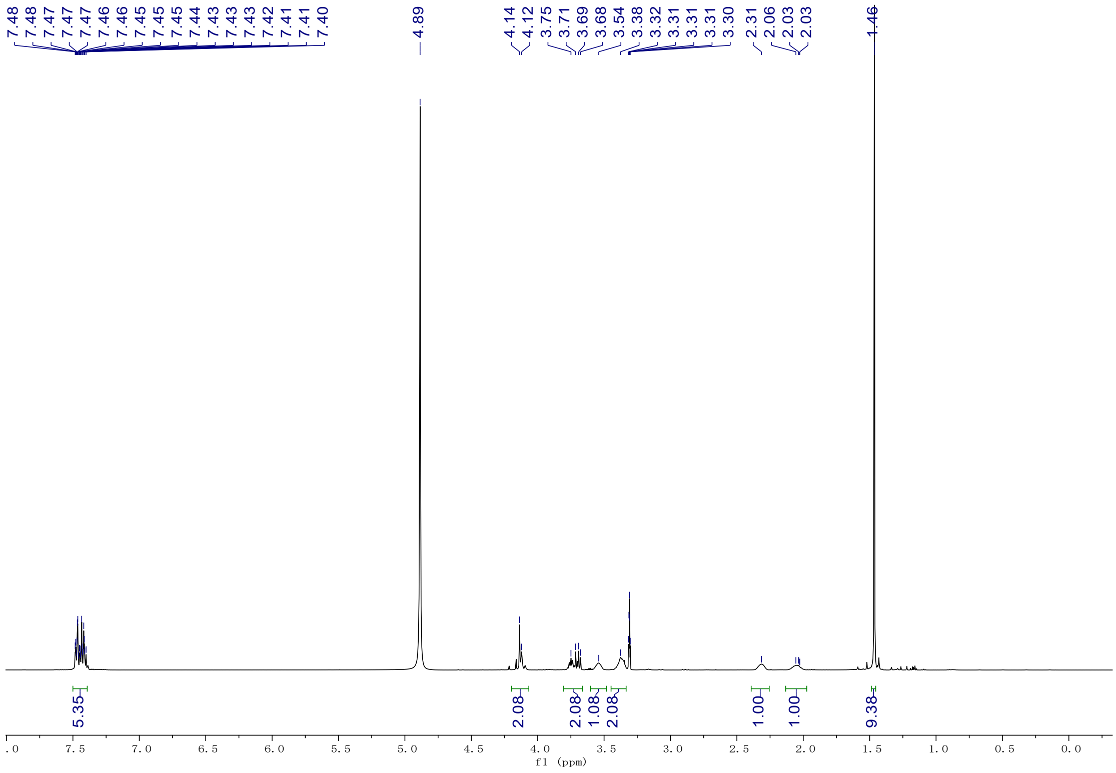


**Figure 16.** The $^{1}H$ NMR spectrum of **1a** in chloroform-*d* (500 MHz)

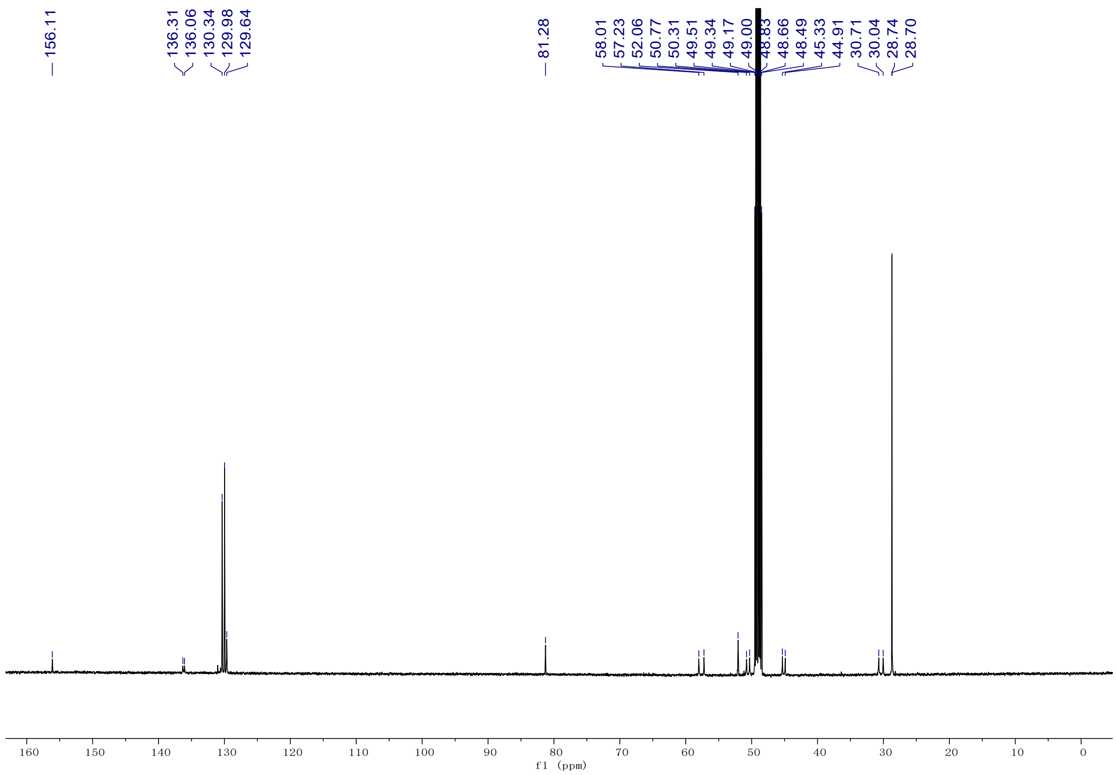


**Figure 17.** The $^{13}C$ NMR spectrum of **1a** in chloroform-*d* (125 MHz)

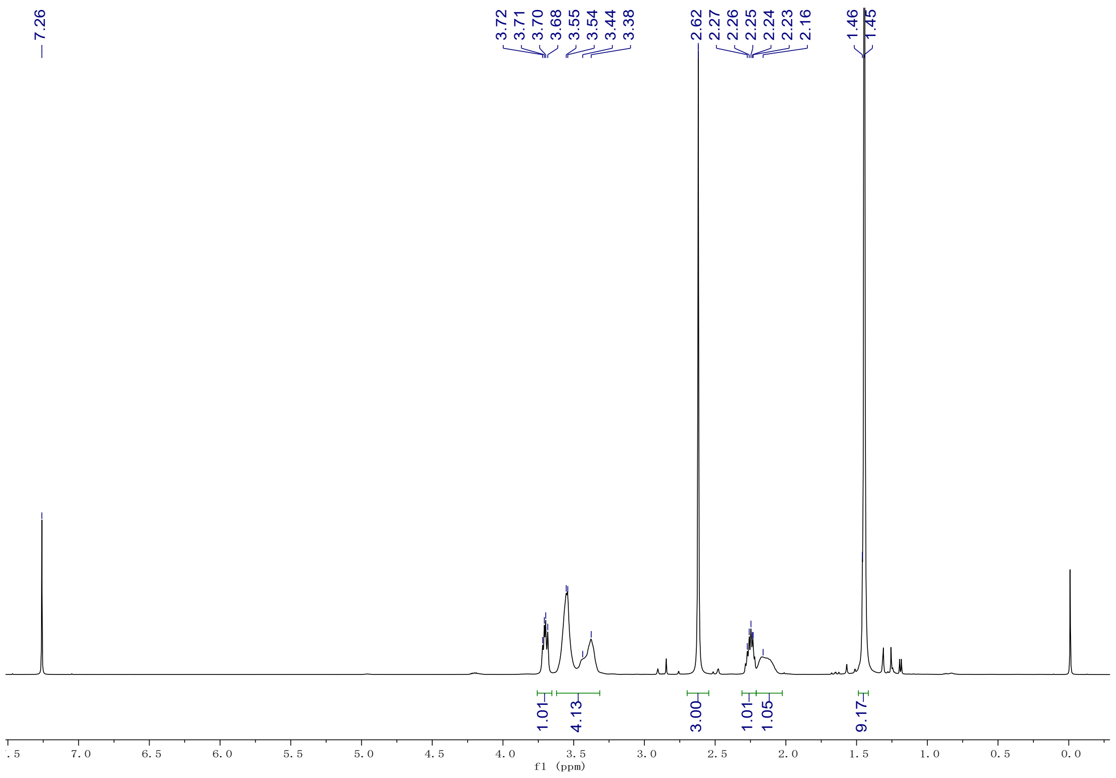


**Figure 18.** The $^{1}$H NMR spectrum of **1b** in chloroform-*d* (500 MHz)

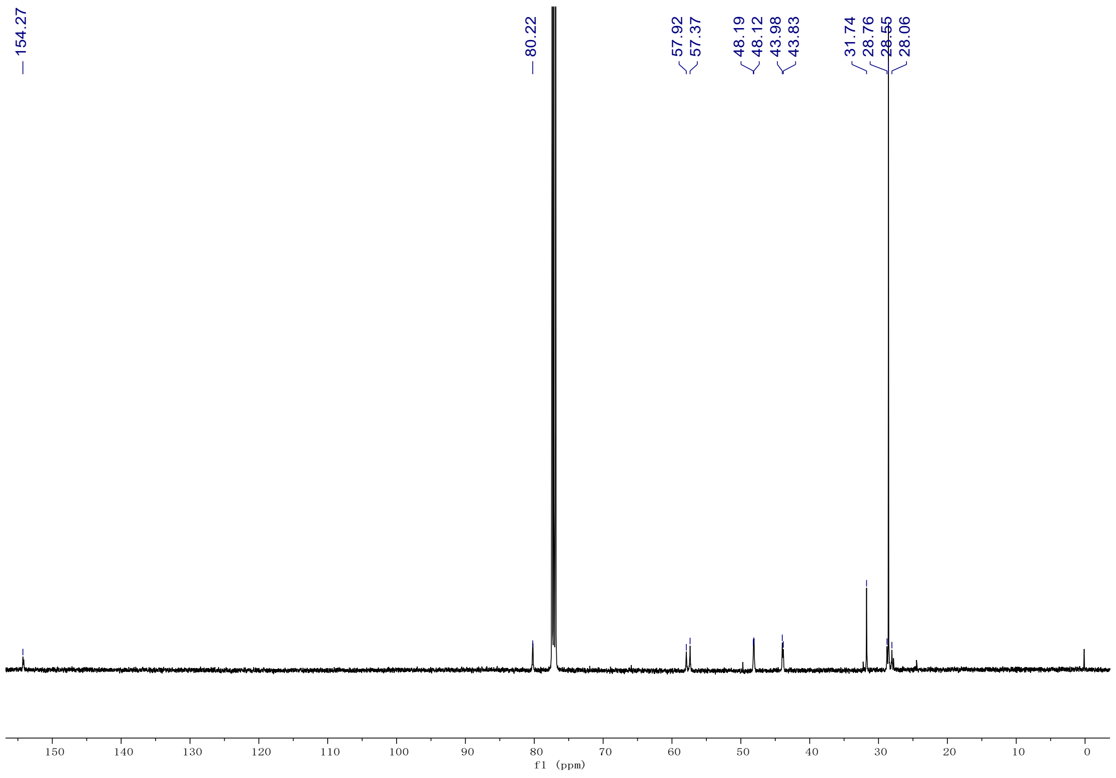


**Figure 19.** The $^{13}$C NMR spectrum of **1b** in chloroform-*d* (125 MHz)

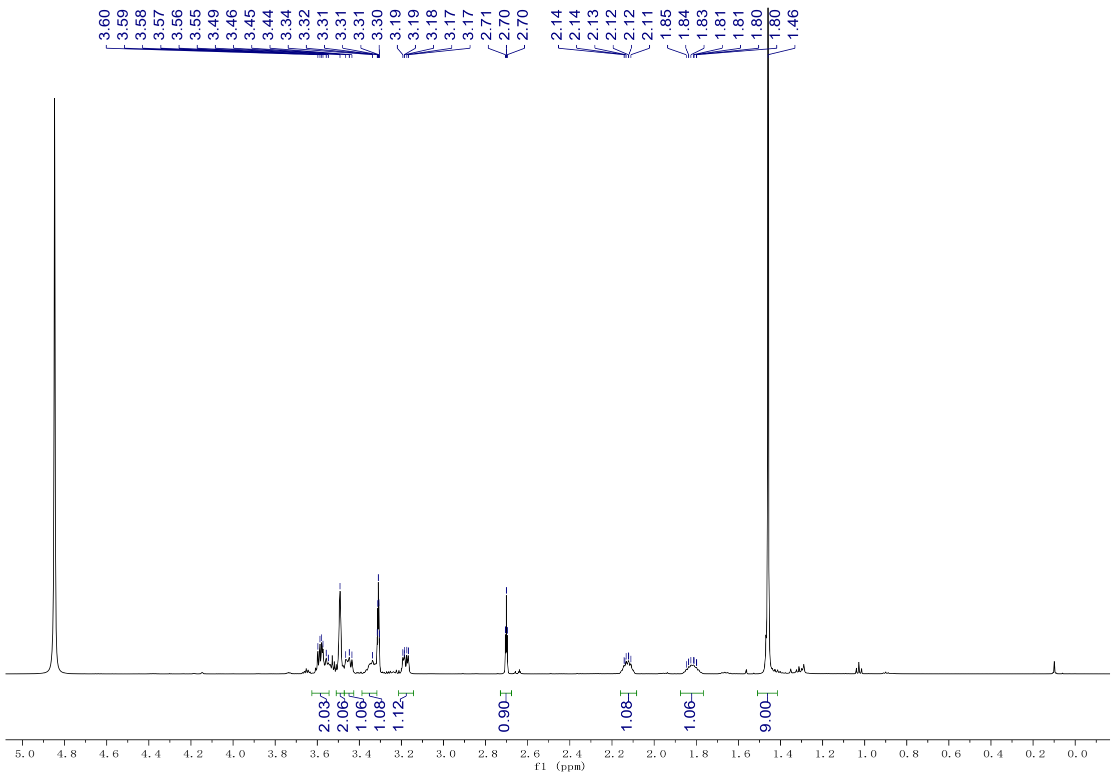


**Figure 20.** The $^{1}H$ NMR spectrum of **1c** in methanol-$d_4$ (600 MHz)

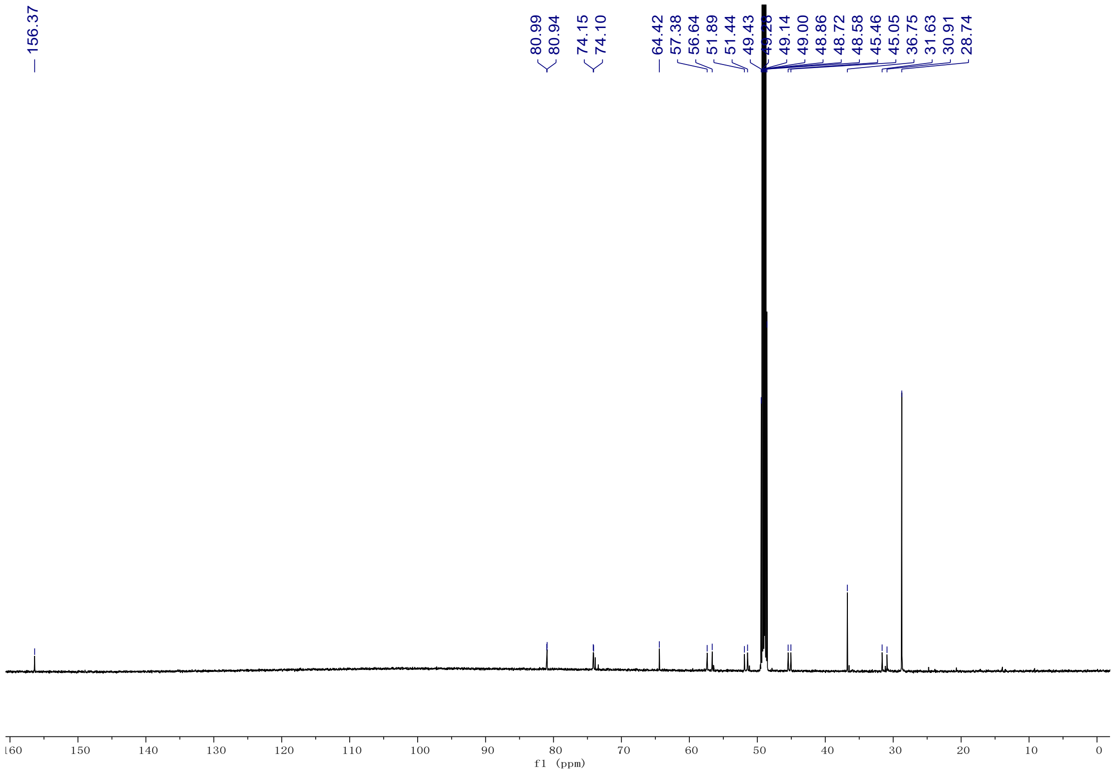


**Figure 21.** The $^{13}C$ NMR spectrum of **1c** in methanol-$d_4$ (150 MHz)

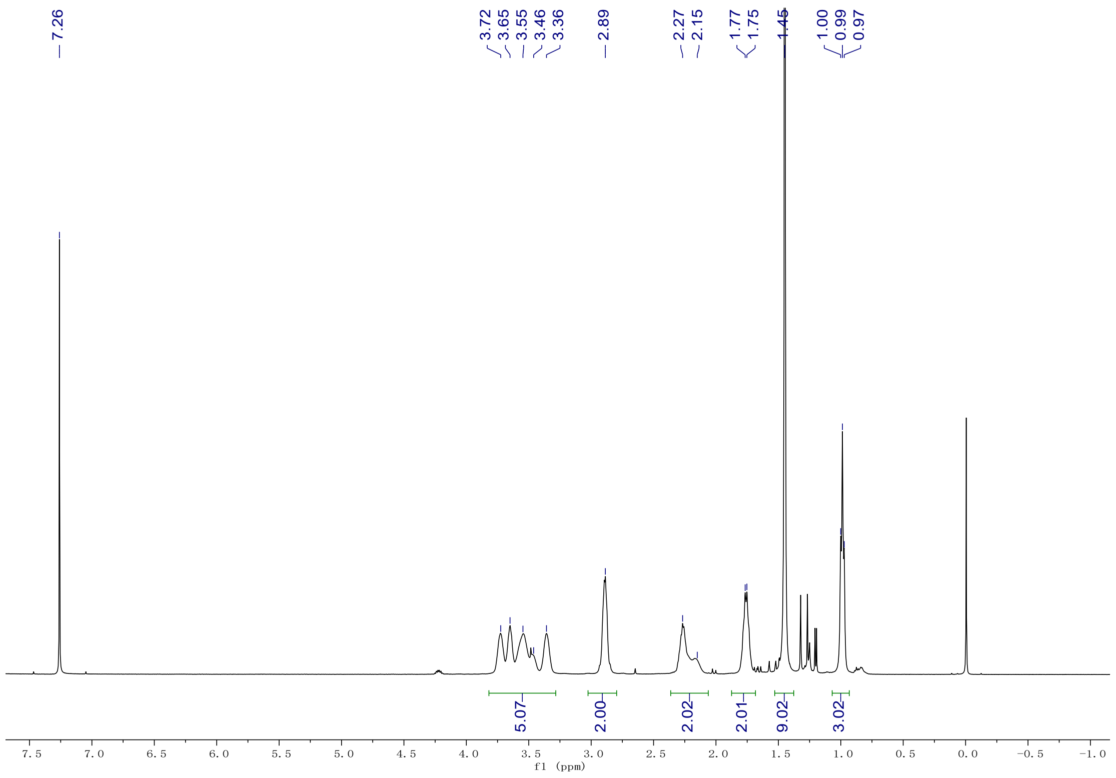


**Figure 22.** The $^{1}H$ NMR spectrum of **1d** in chloroform-*d* (500 MHz)

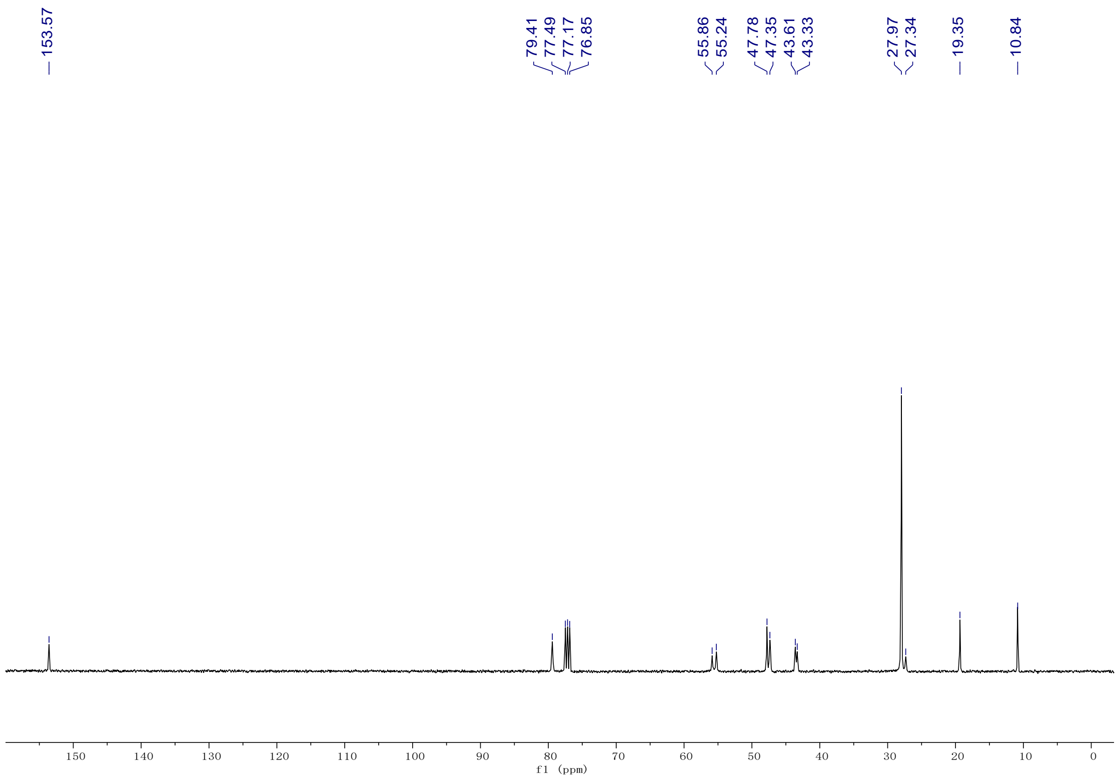


**Figure 23.** The $^{13}C$ NMR spectrum of **1d** in chloroform-*d* (125 MHz)

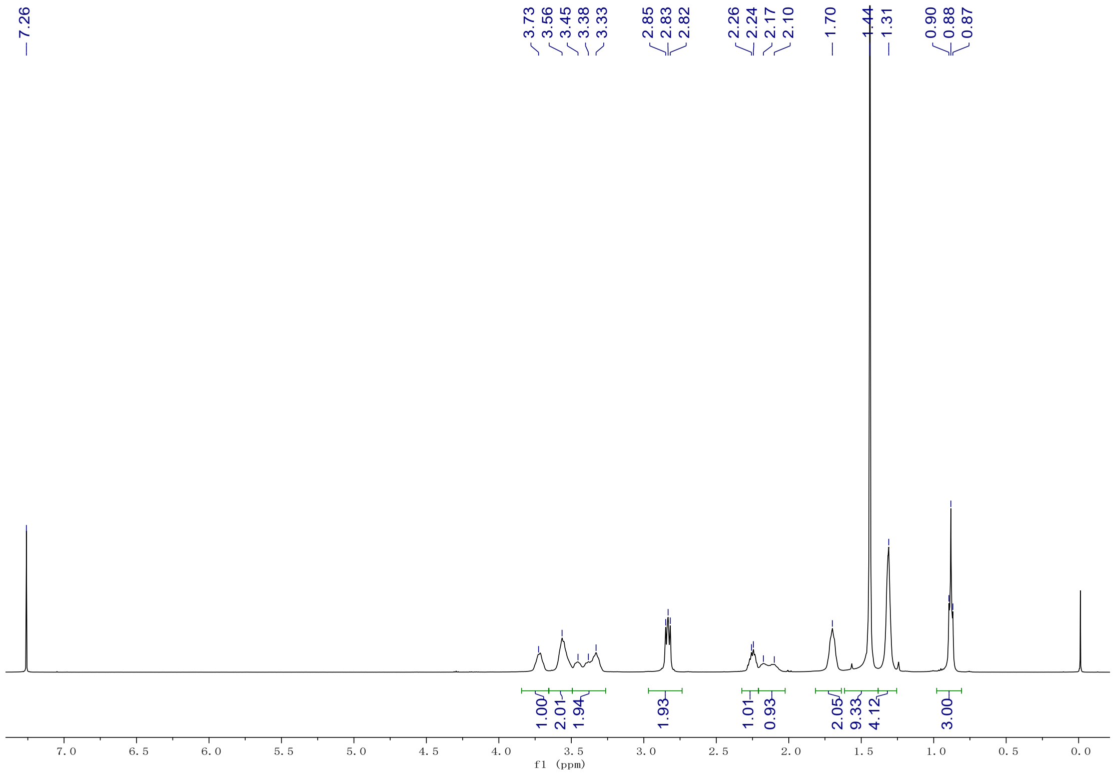


**Figure 24.** The $^{1}H$ NMR spectrum of **1e** in chloroform-*d* (500 MHz)

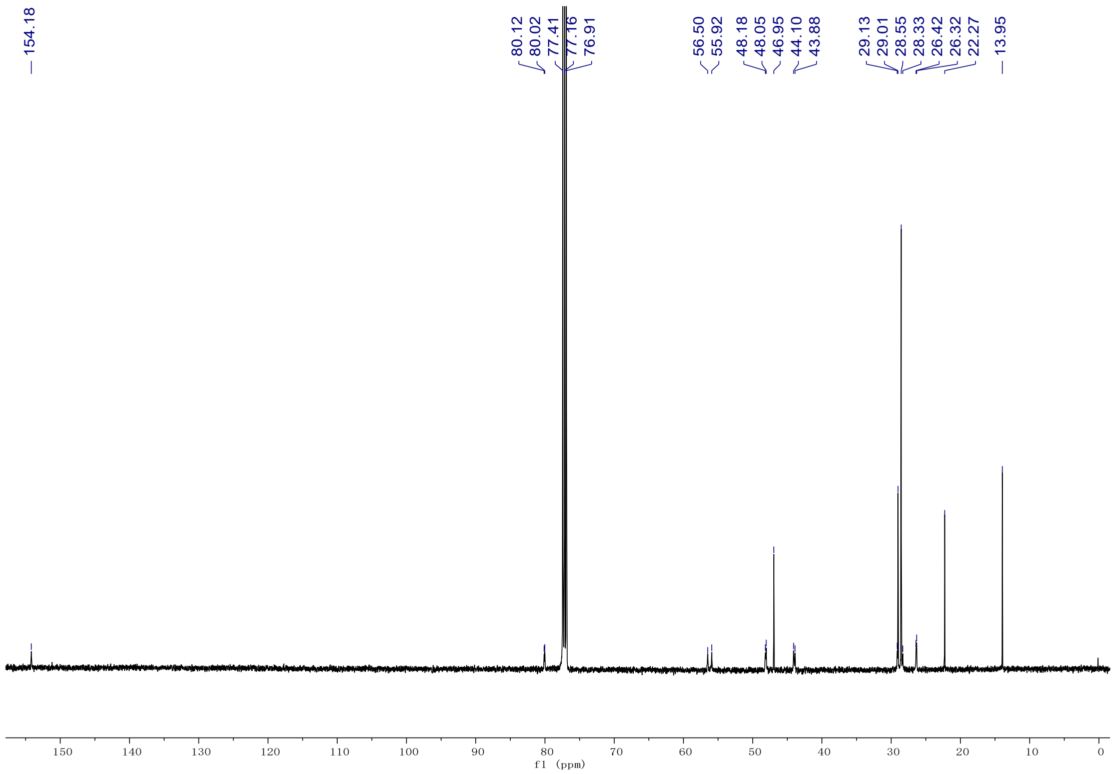


**Figure 25.** The $^{13}C$ NMR spectrum of **1e** in chloroform-*d* (125 MHz)

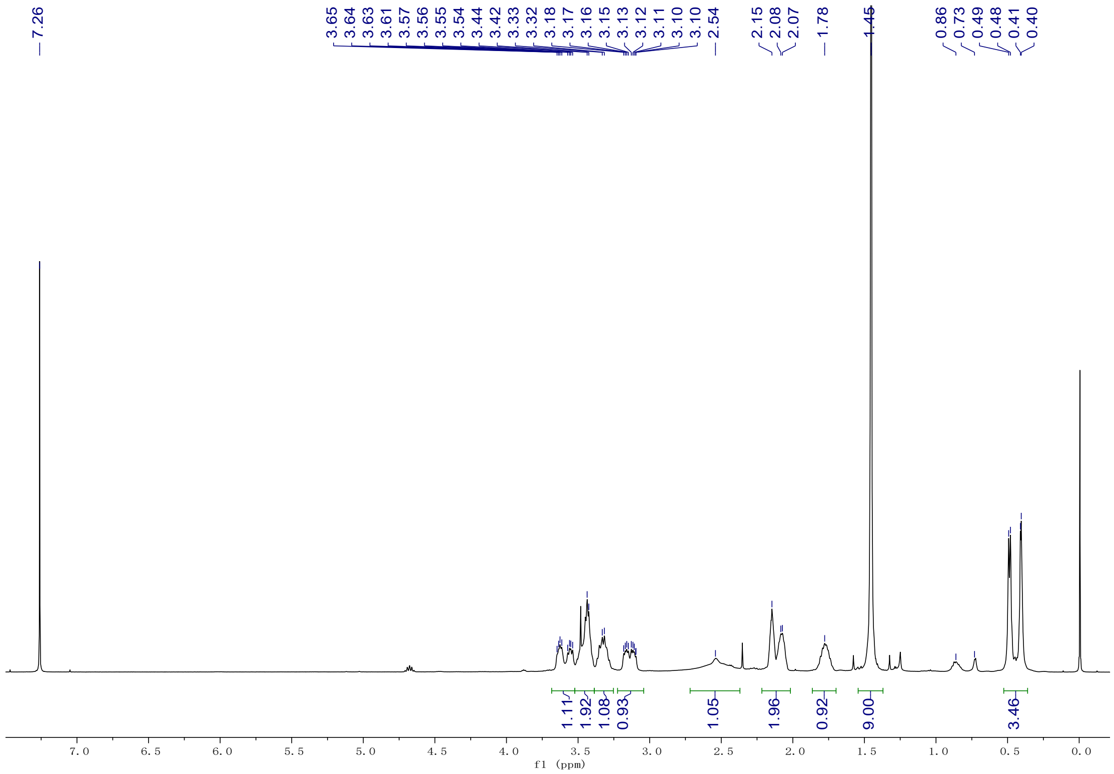


**Figure 26.** The $^{1}H$ NMR spectrum of **1f** in chloroform-*d* (500 MHz)

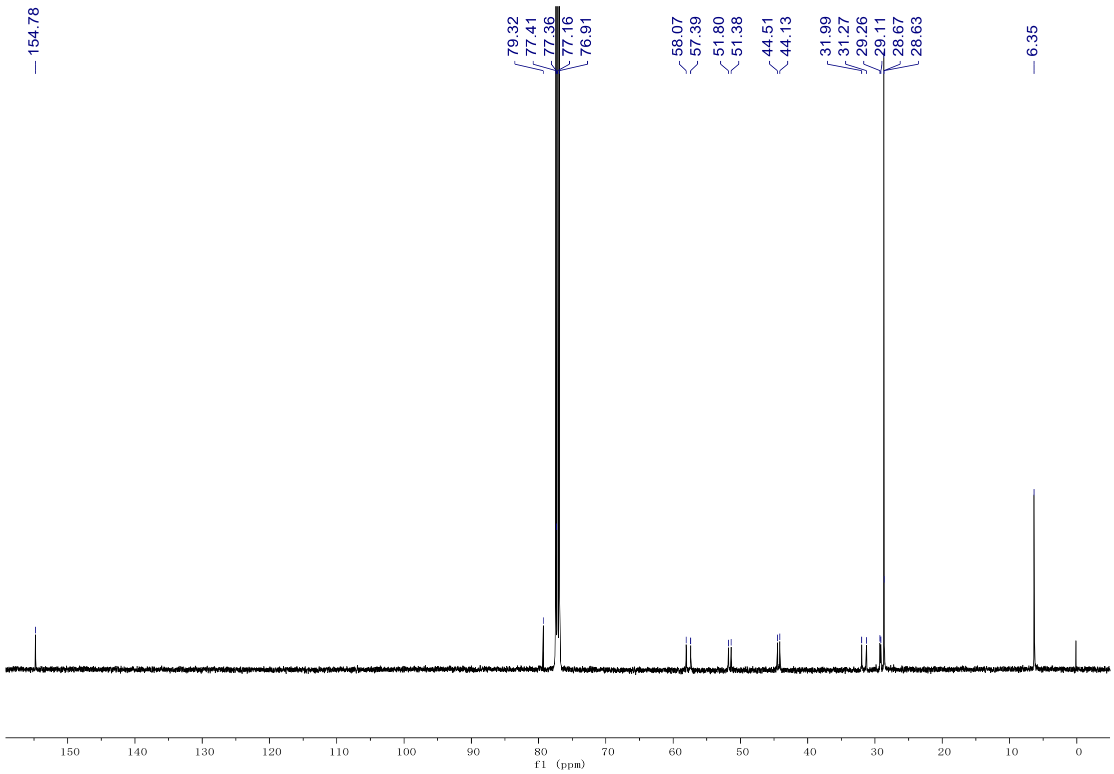


**Figure 27.** The $^{13}C$ NMR spectrum of **2f** in chloroform-*d* (125 MHz)

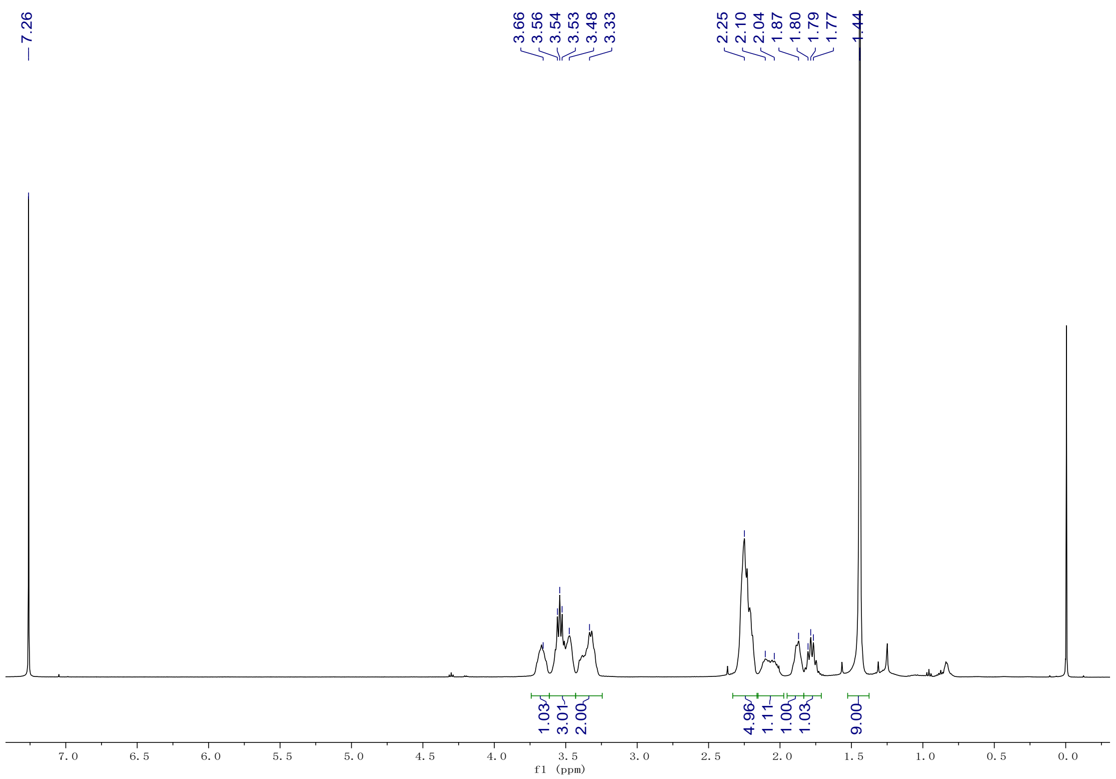

**Figure 28.** The $^{1}$H NMR spectrum of **1g** in chloroform-*d* (500 MHz)

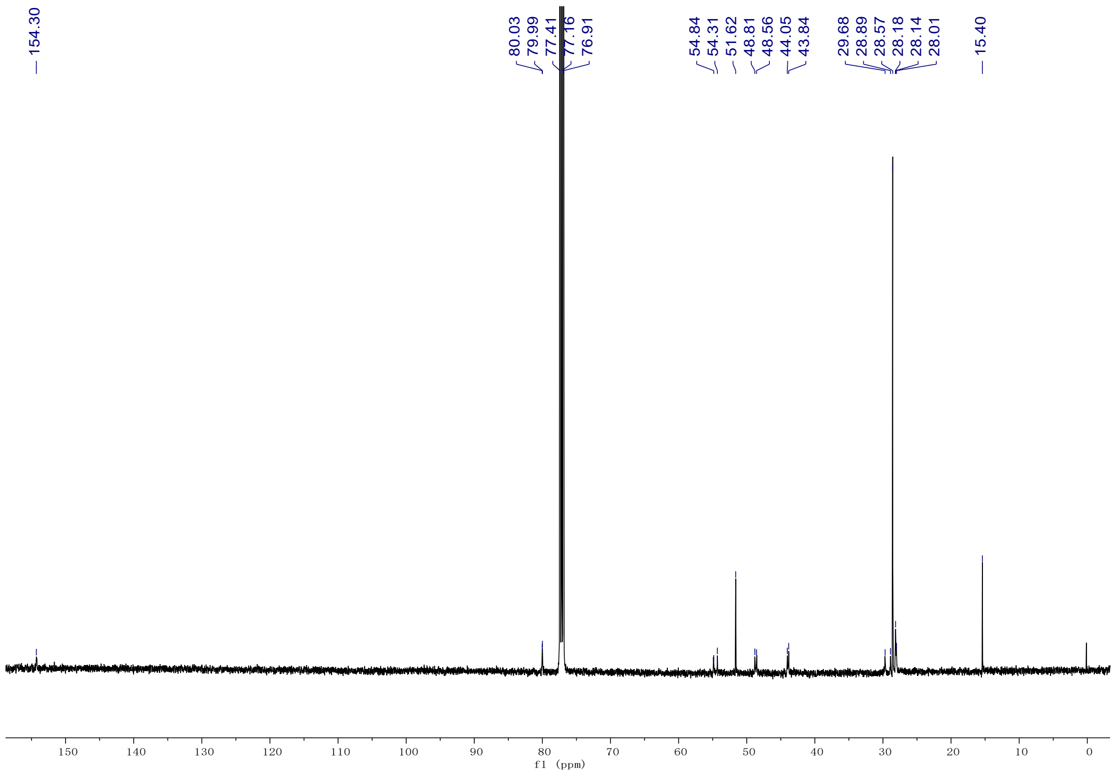

**Figure 29.** The $^{13}$C NMR spectrum of **1g** in chloroform-*d* (125 MHz)

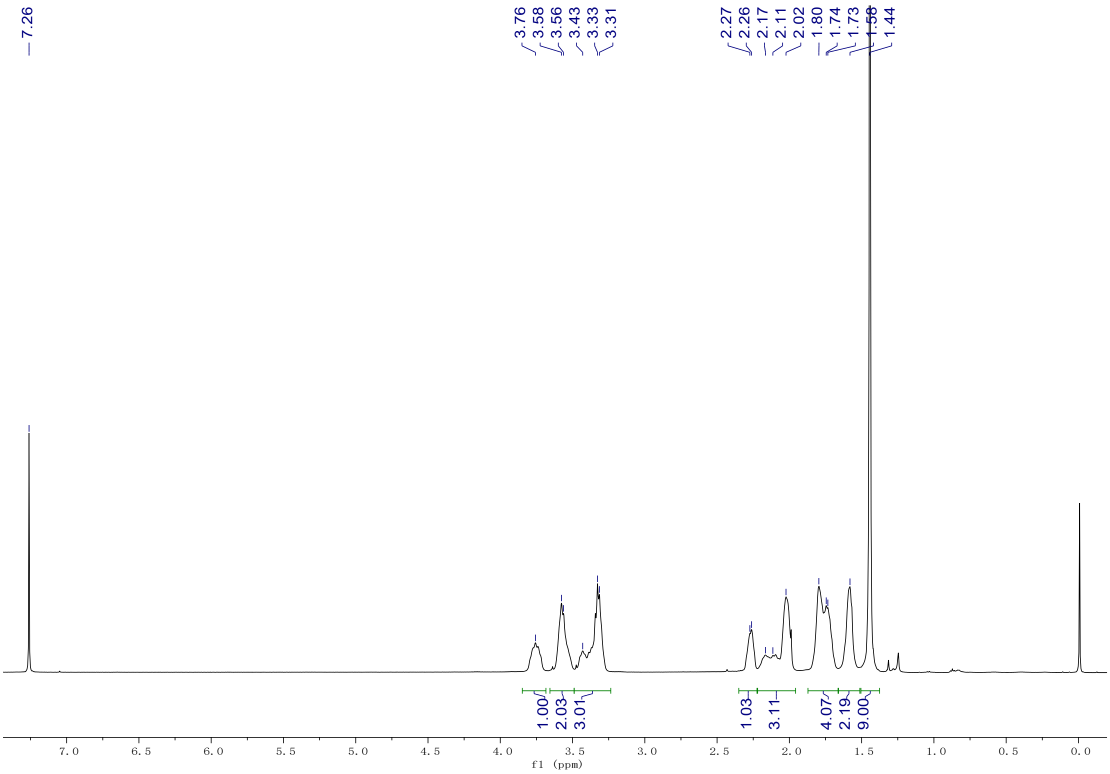


**Figure 30.** The $^{1}H$ NMR spectrum of **1h** in chloroform-*d* (500 MHz)

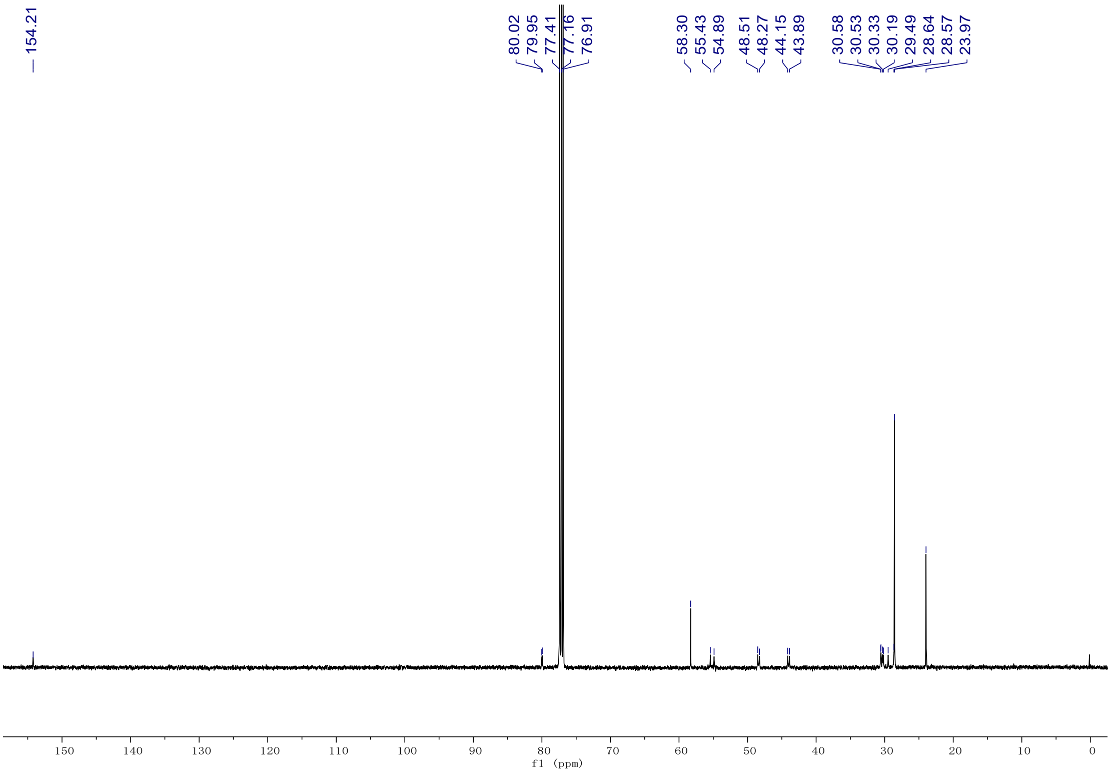


**Figure 31.** The $^{13}C$ NMR spectrum of **1h** in chloroform-*d* (125 MHz)

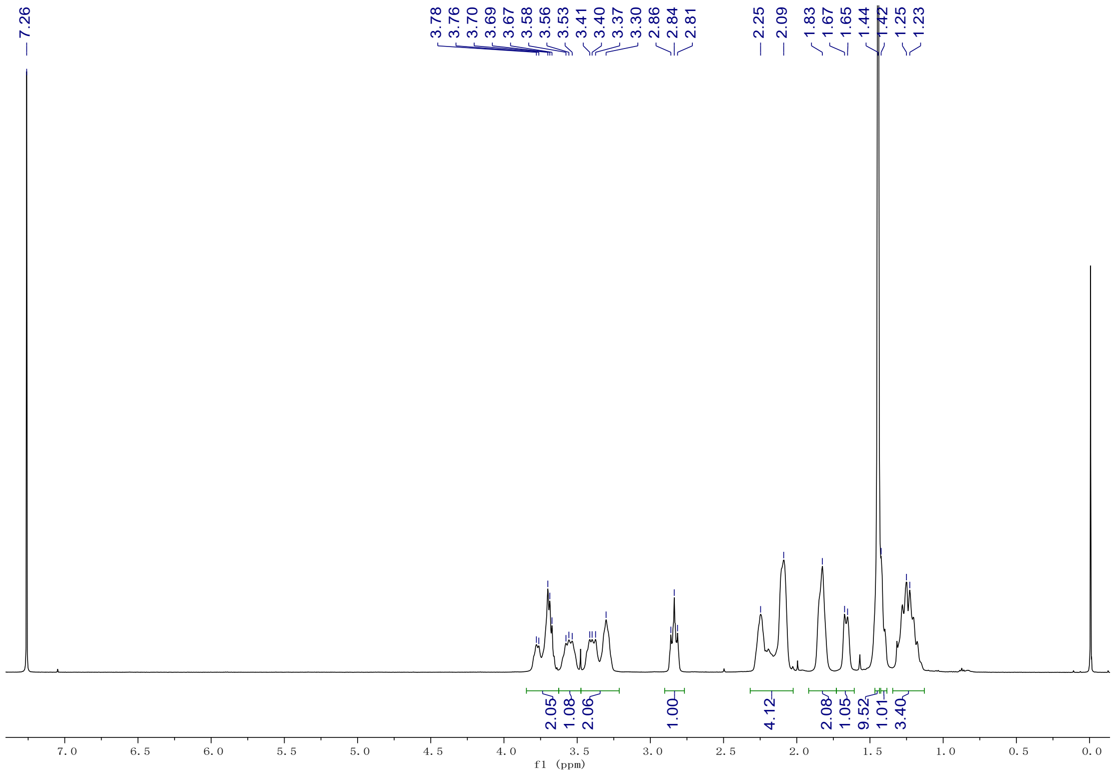


**Figure 32.** The $^{1}$H NMR spectrum of **1i** in chloroform-*d* (500 MHz)

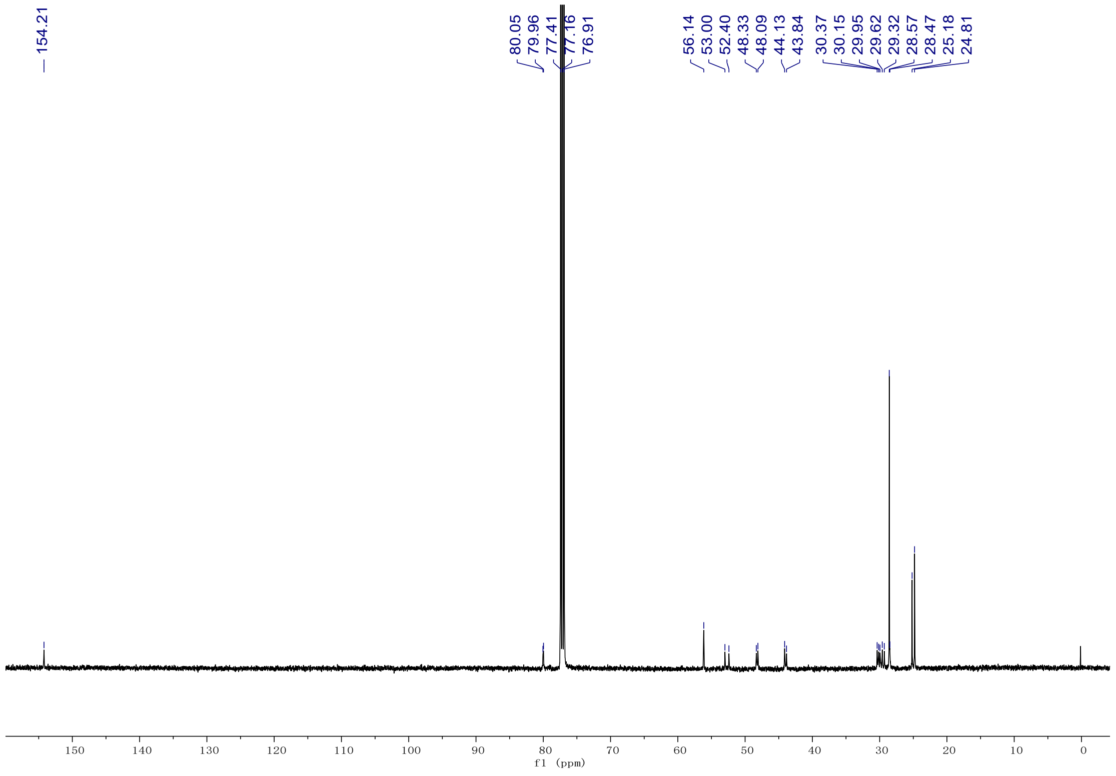


**Figure 33.** The $^{13}$C NMR spectrum of **1i** in chloroform-*d* (125 MHz)

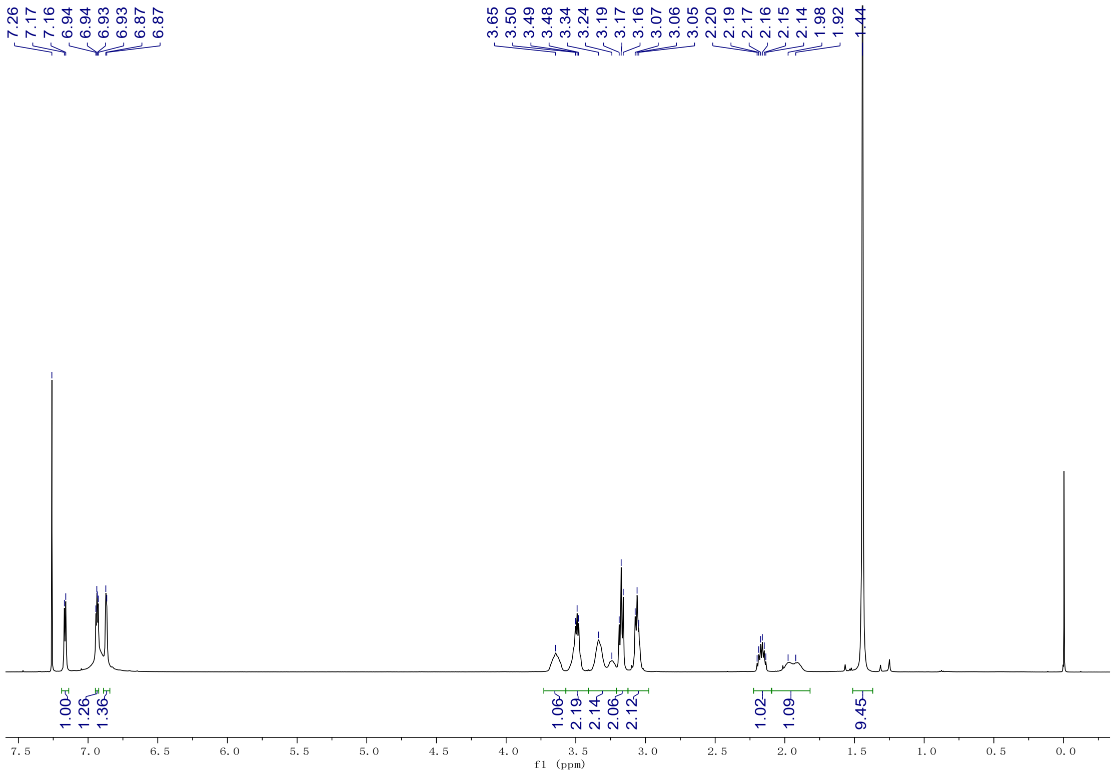


**Figure 34.** The $^{1}$H NMR spectrum of **1j** in chloroform-*d* (500 MHz)

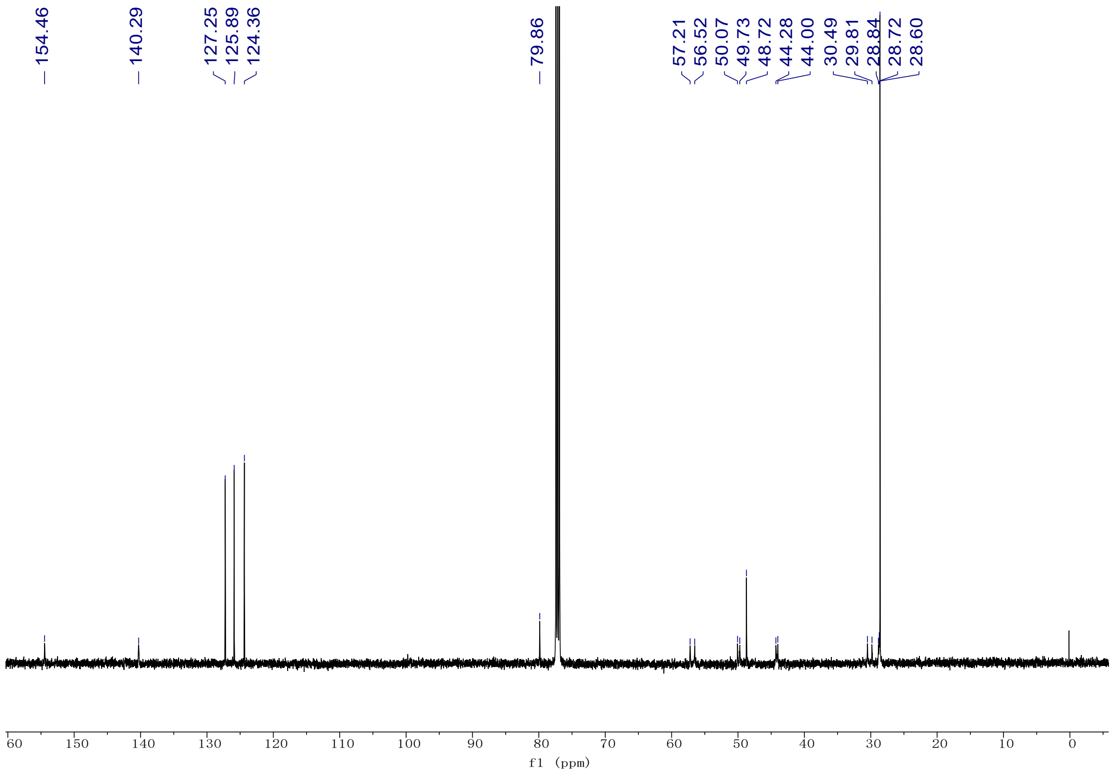


**Figure 35.** The $^{13}$C NMR spectrum of **1j** in chloroform-*d* (125 MHz)

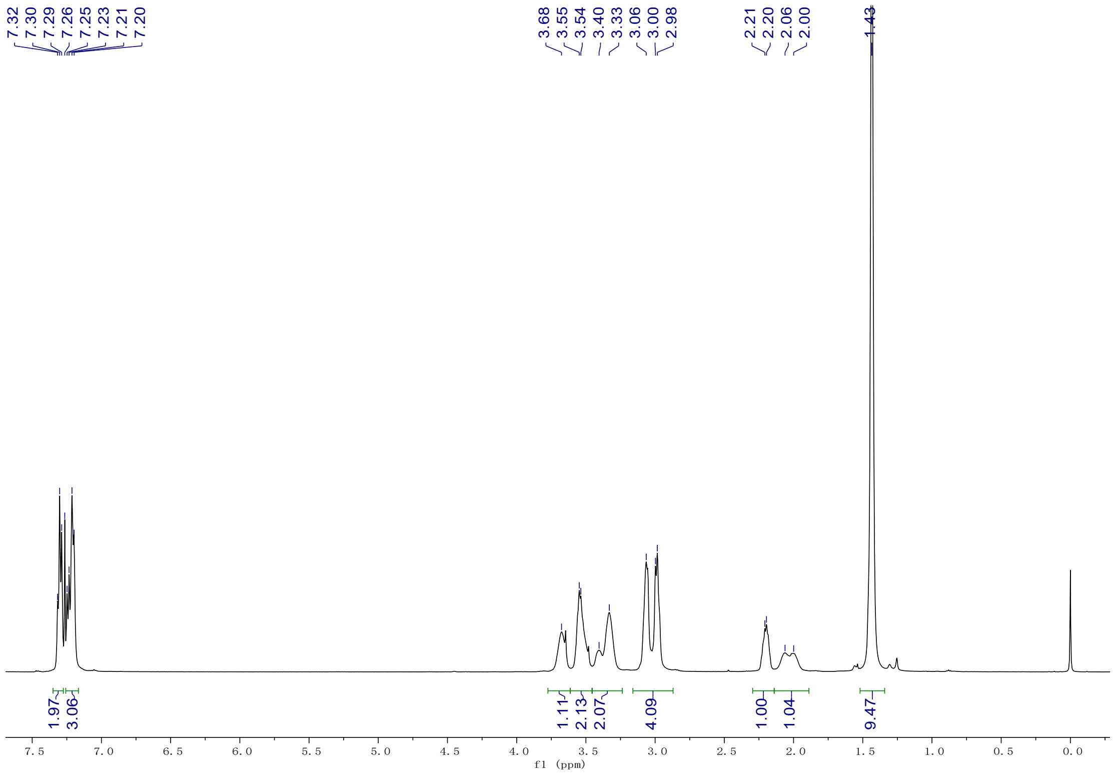


**Figure 36.** The $^{1}$H NMR spectrum of **1k** in chloroform-*d* (500 MHz)

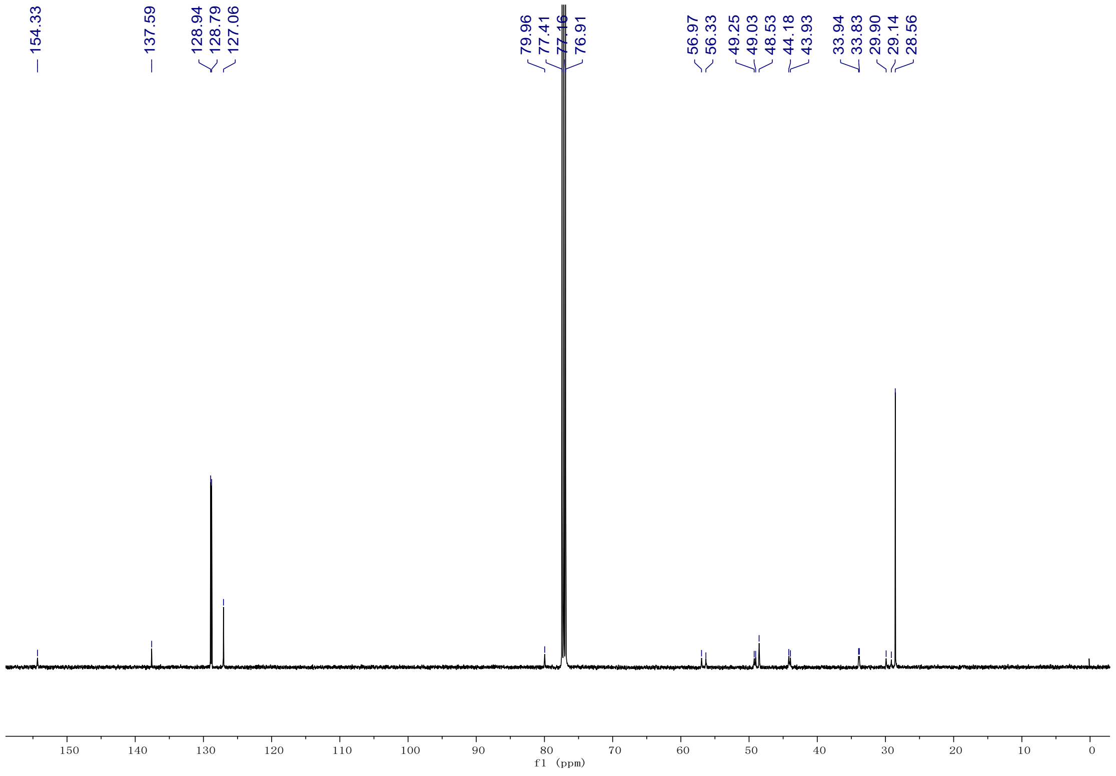


**Figure 37.** The $^{13}$C NMR spectrum of **1k** in chloroform-*d* (125 MHz)

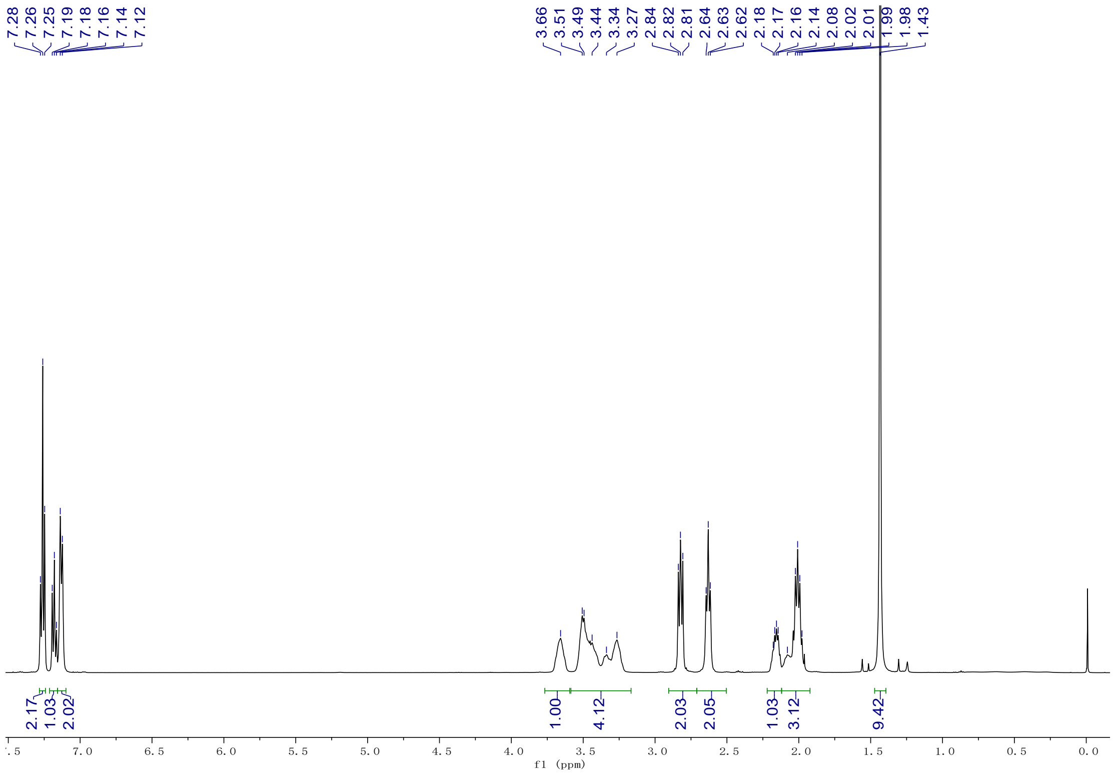


**Figure 38.** The $^{1}$H NMR spectrum of **1l** in chloroform-*d* (500 MHz)

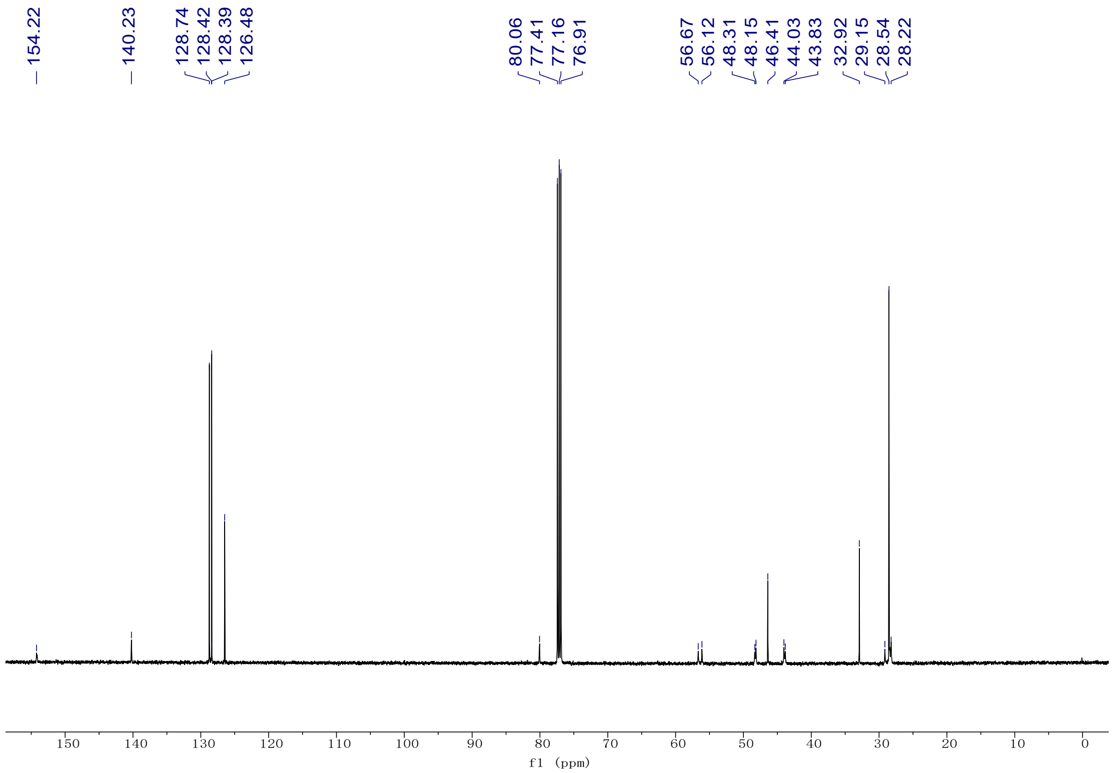


**Figure 39.** The $^{13}$C NMR spectrum of **1l** in chloroform-*d* (125 MHz)

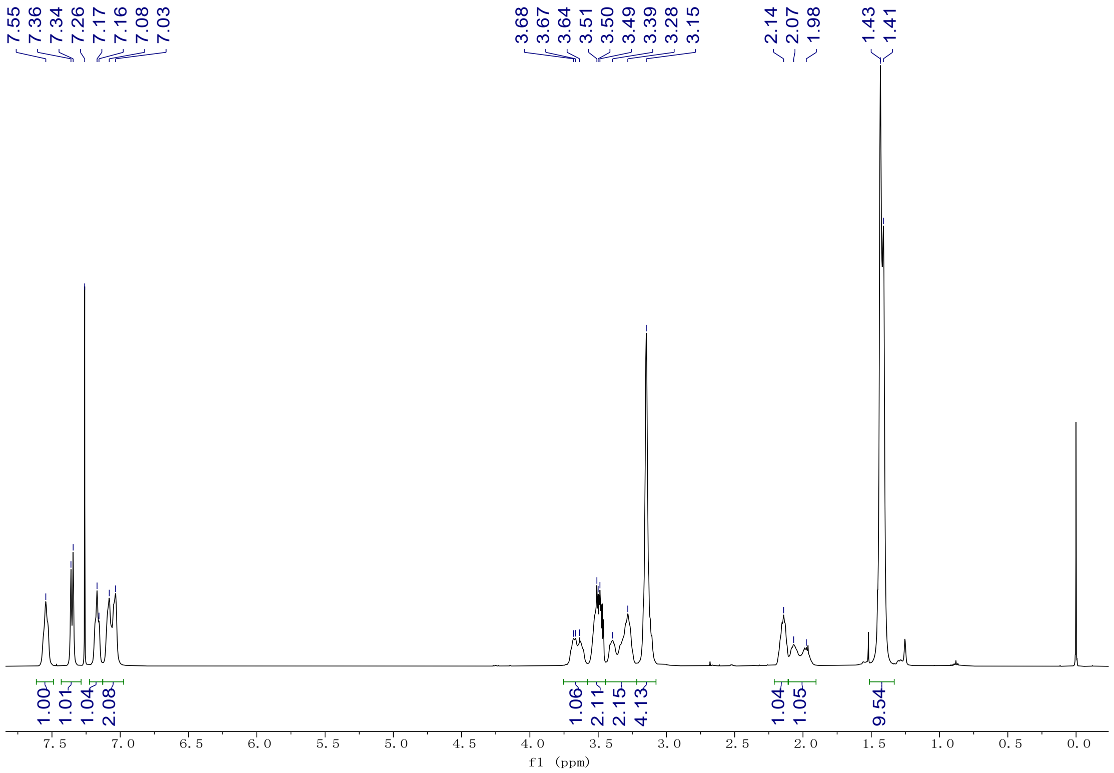


**Figure 40.** The $^{1}$H NMR spectrum of **1m** in chloroform-*d* (500 MHz)

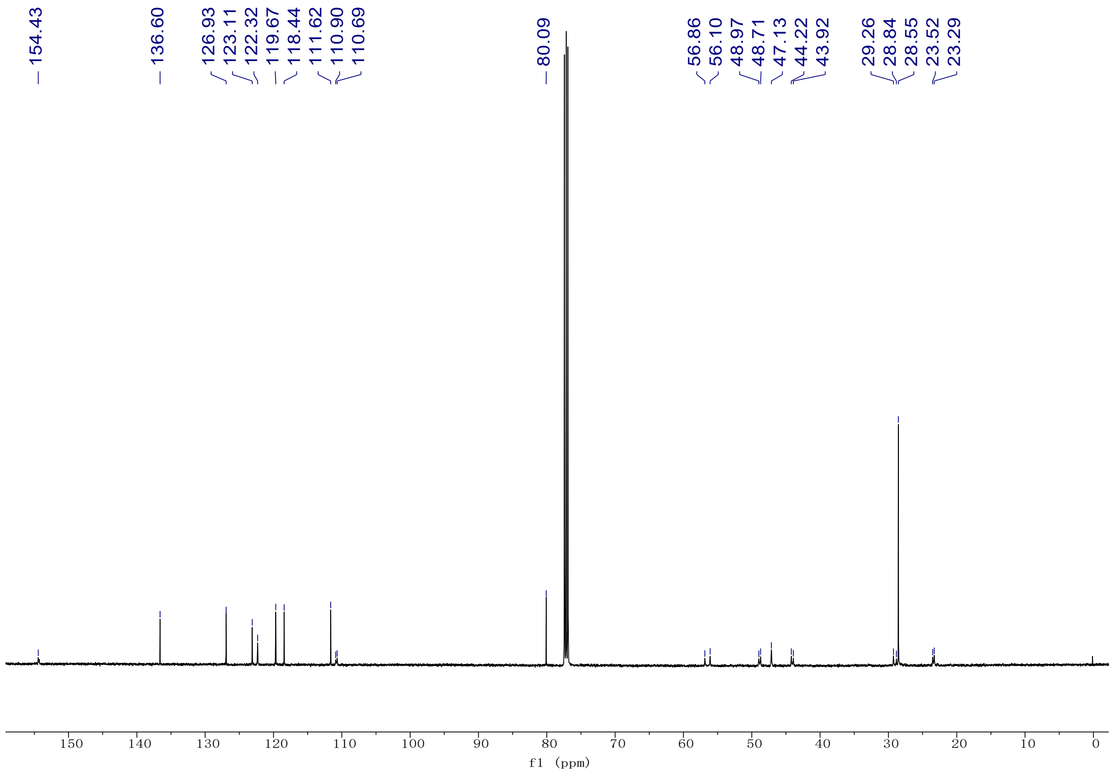


**Figure 41.** The $^{13}$C NMR spectrum of **1m** in chloroform-*d* (125 MHz)

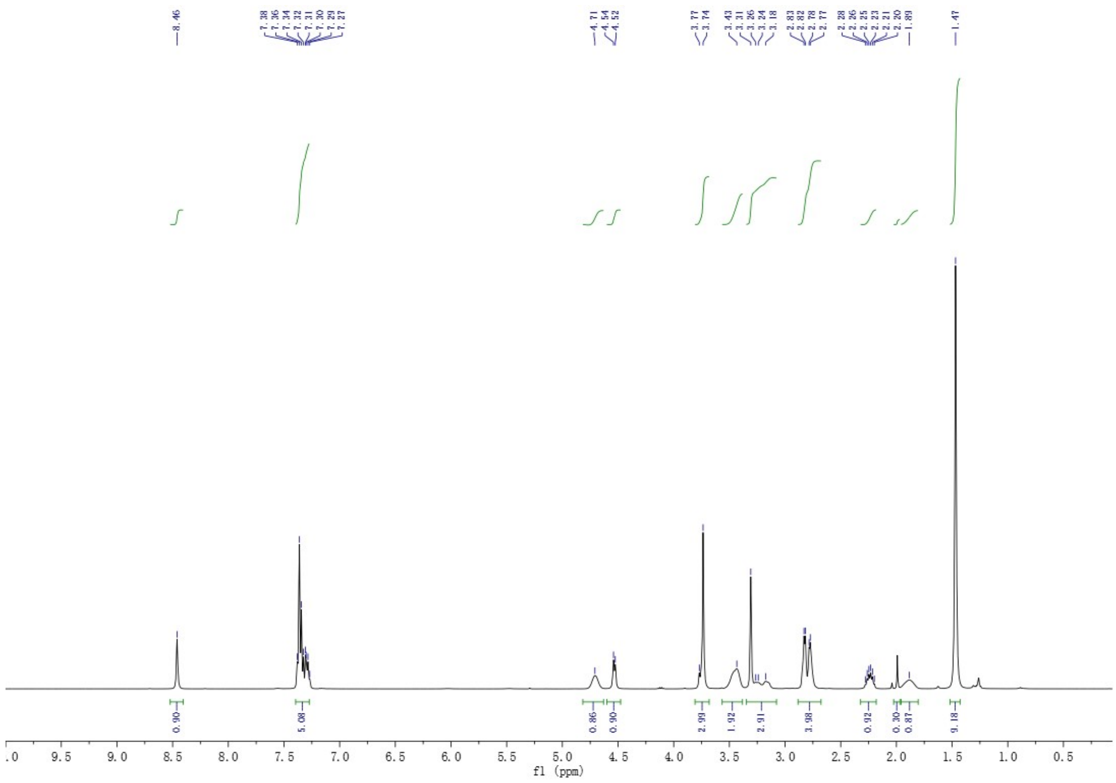

**Figure 42.** The $^{1}H$ NMR spectrum of **2** in chloroform-*d* (400 MHz)

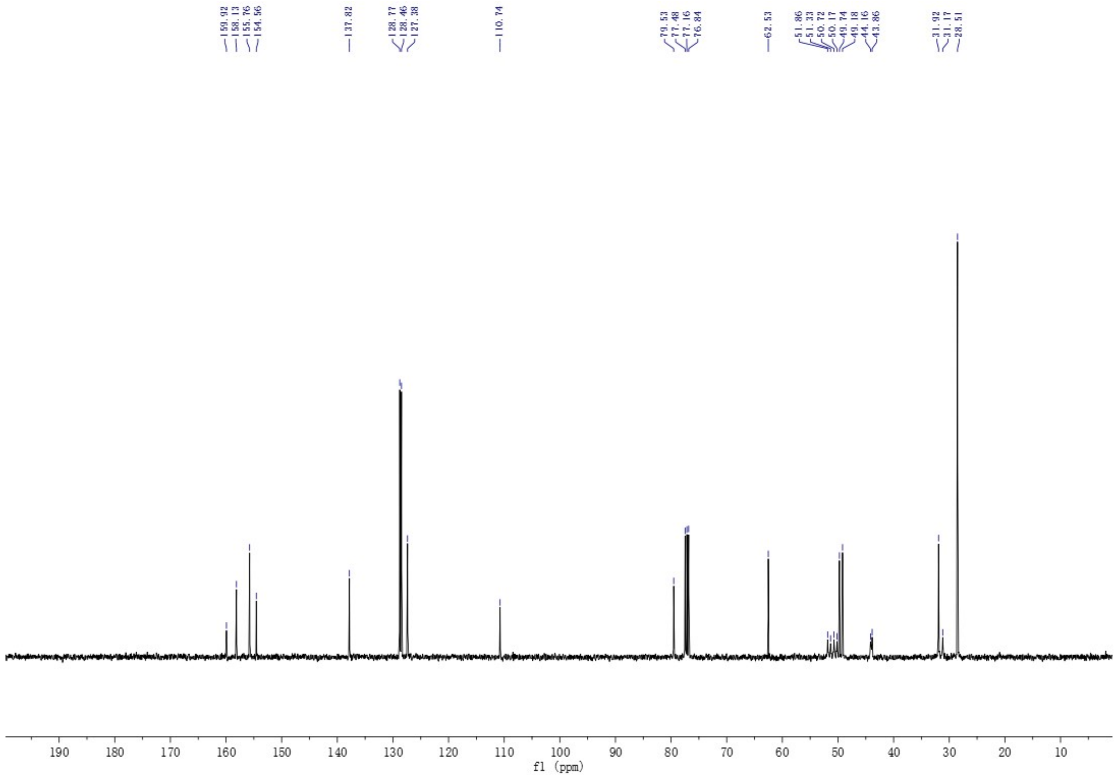

**Figure 43.** The $^{13}C$ NMR spectrum of **2** in chloroform-*d* (100 MHz)

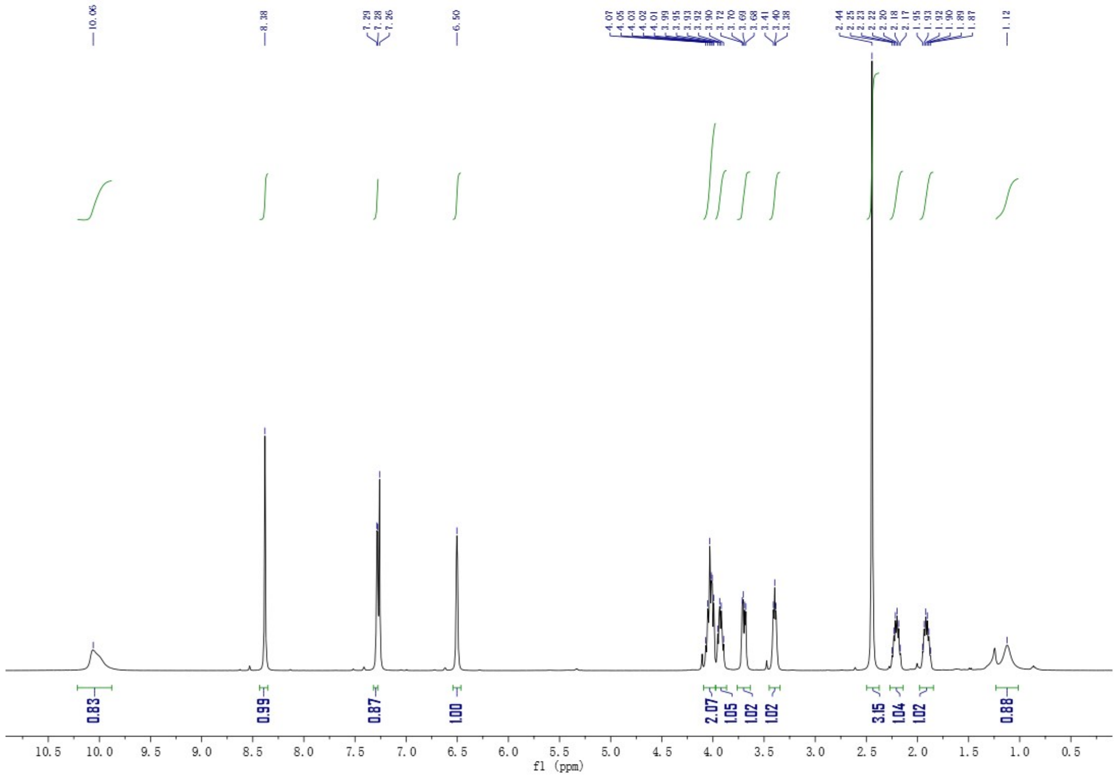

**Figure 44.** The $^{1}H$ NMR spectrum of **3** in chloroform-*d* (400 MHz)

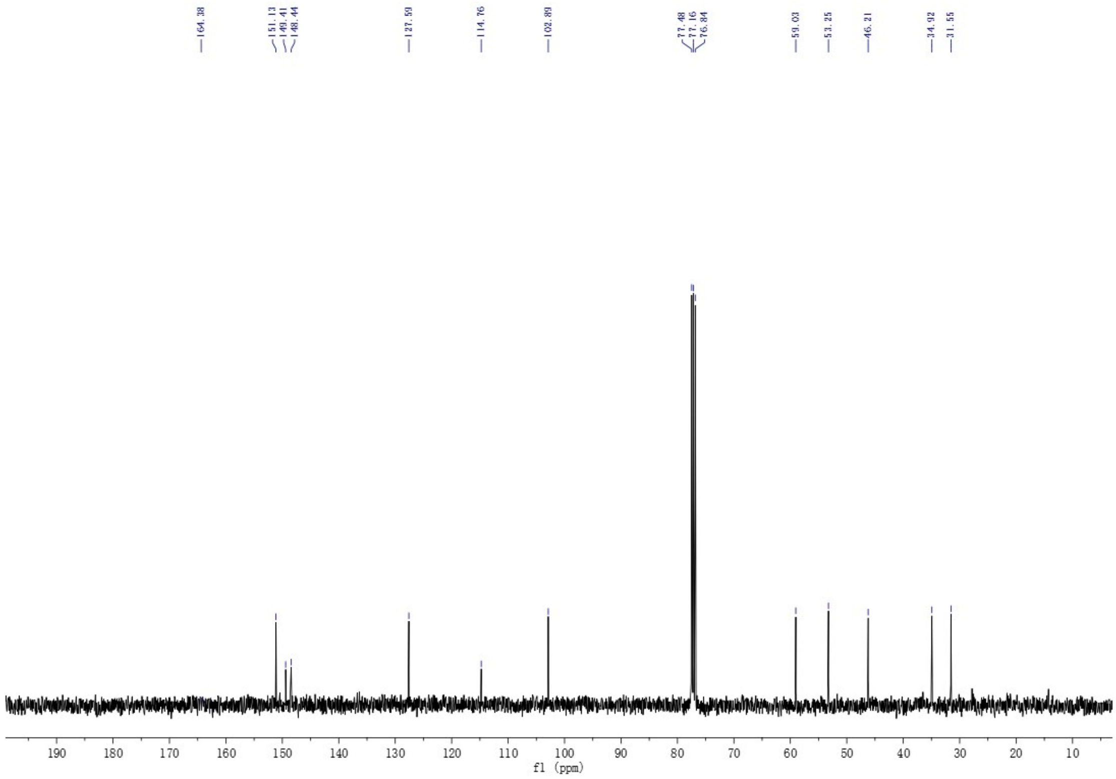

**Figure 45.** The $^{13}C$ NMR spectrum of **3** in chloroform-*d* (100 MHz)

## HR-ESI-MS spectra of amine products

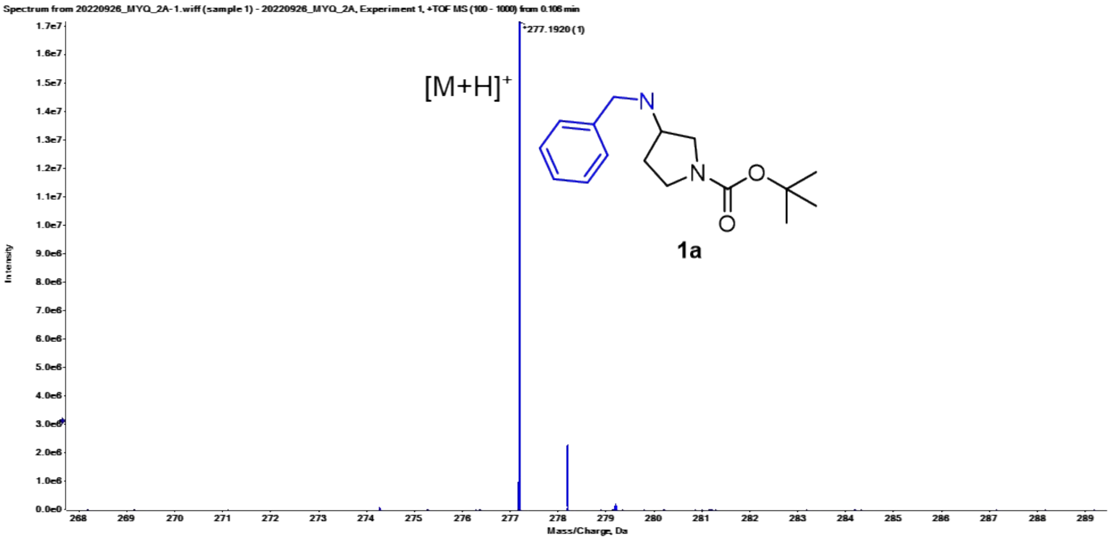


**Figure 46.** The HR-ESI-MS spectrum of **1a**.

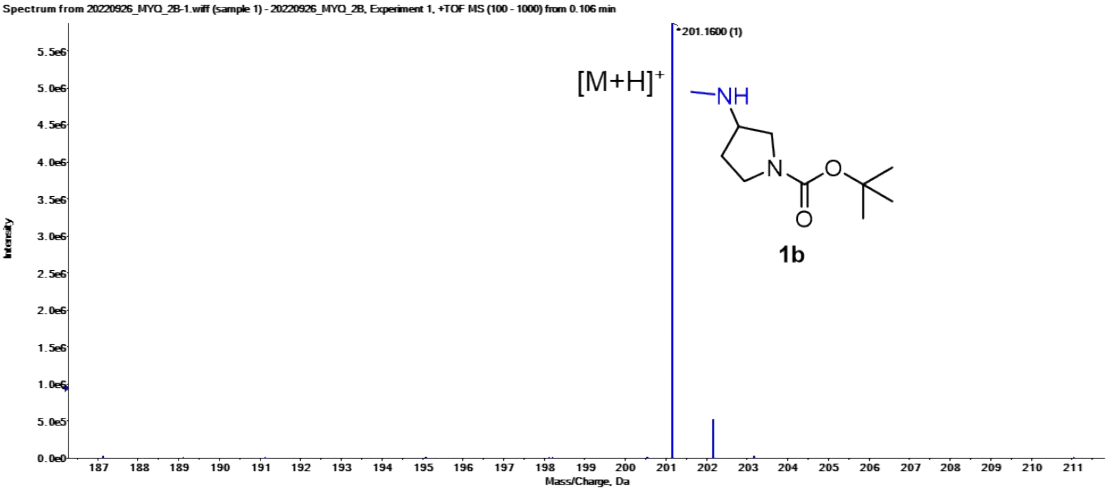


**Figure 47.** The HR-ESI-MS spectrum of **1b**.

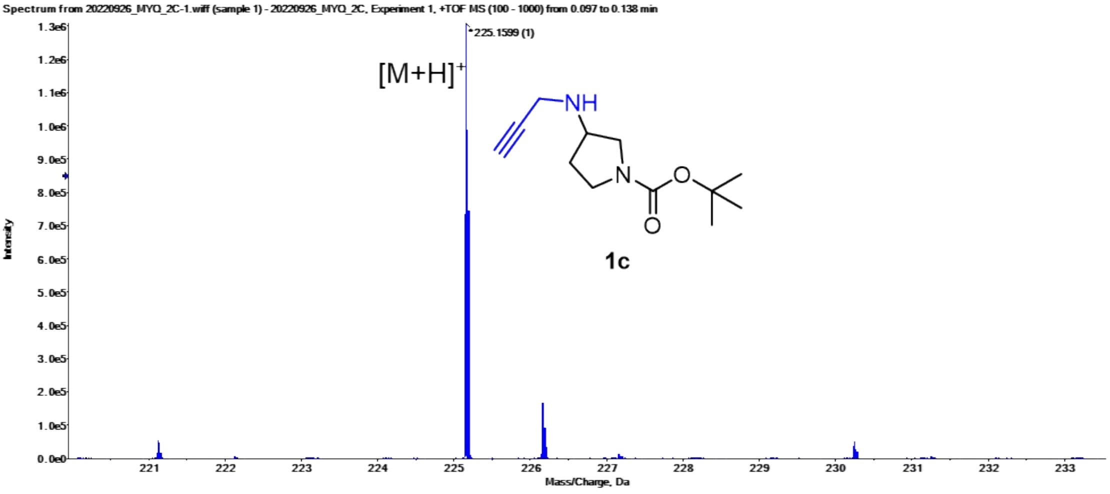


**Figure 48.** The HR-ESI-MS spectrum of **1c**.

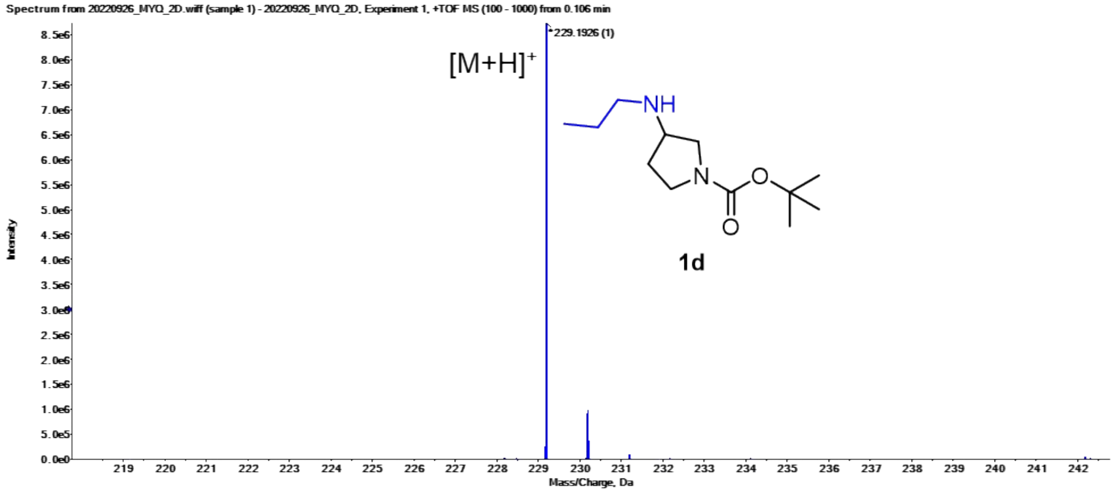


**Figure 49.** The HR-ESI-MS spectrum of **1d**.

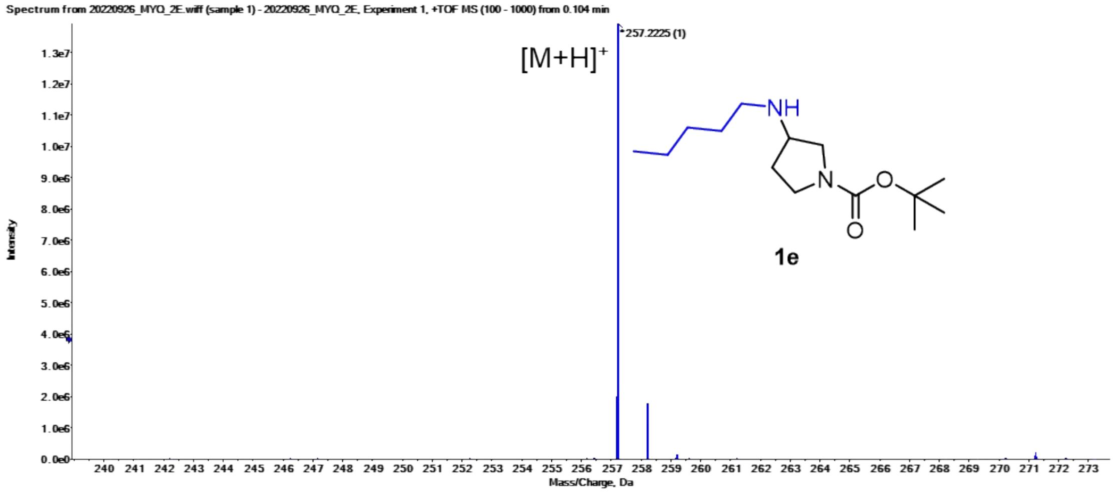


**Figure 50.** The HR-ESI-MS spectrum of **1e**.

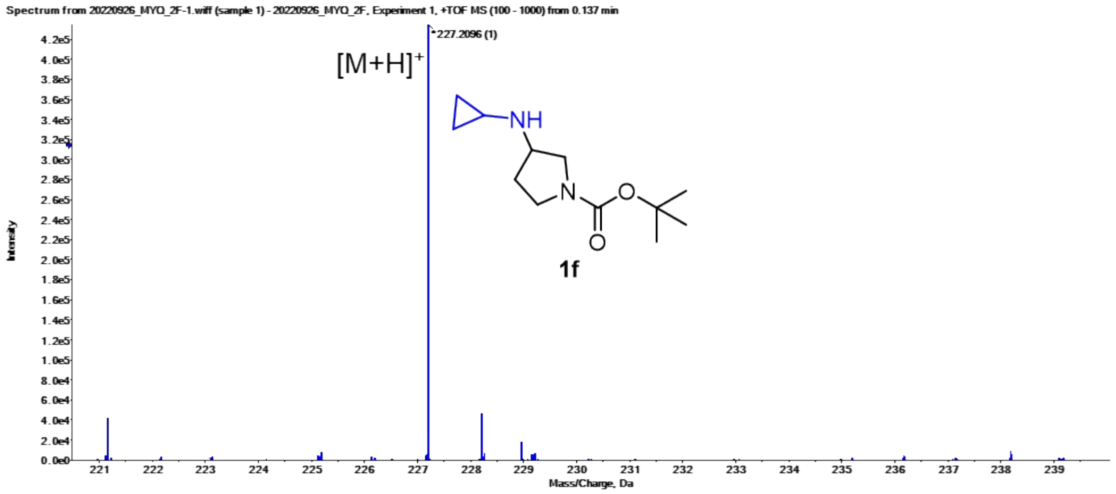


**Figure 51.** The HR-ESI-MS spectrum of **1f**.

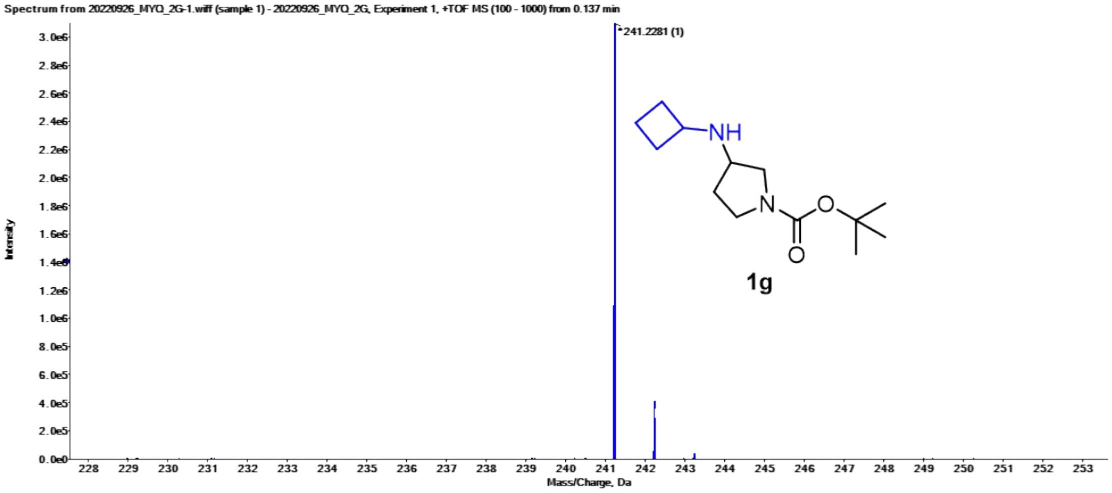


**Figure 52.** The HR-ESI-MS spectrum of **1g**.

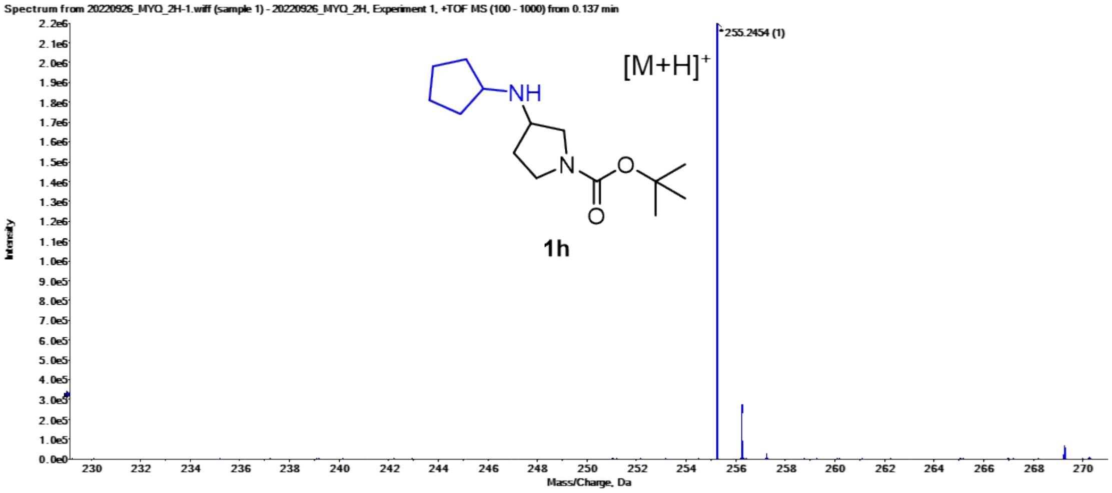


**Figure 53.** The HR-ESI-MS spectrum of **1h**.

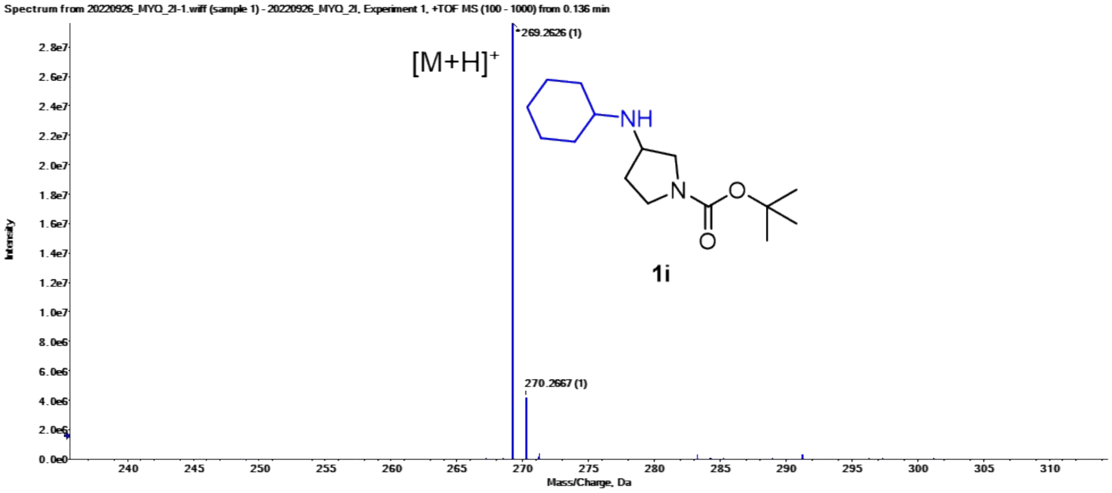


**Figure 54.** The HR-ESI-MS spectrum of **1i**.

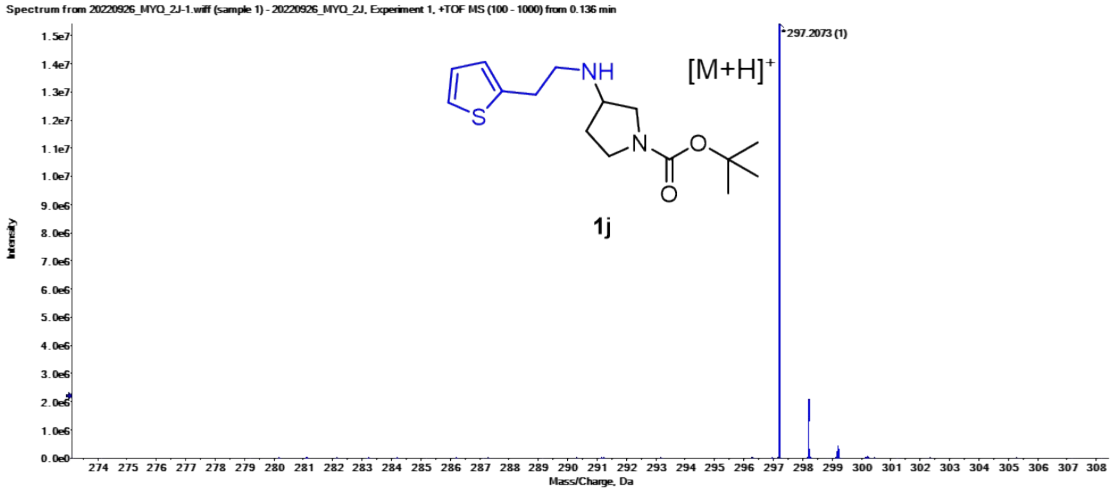


**Figure 55.** The HR-ESI-MS spectrum of **1j**.

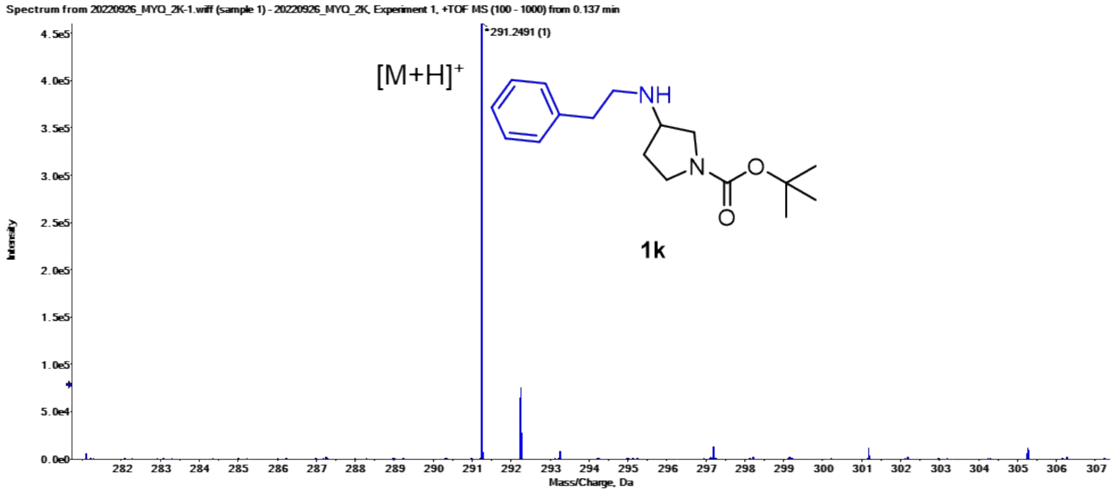


**Figure 56.** The HR-ESI-MS spectrum of **1k**.

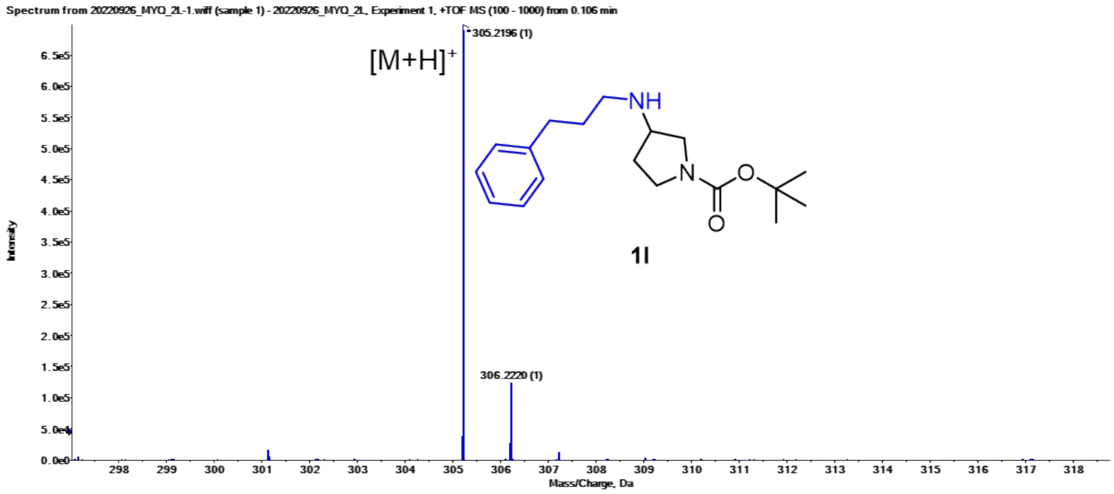


**Figure 57.** The HR-ESI-MS spectrum of **1l**.

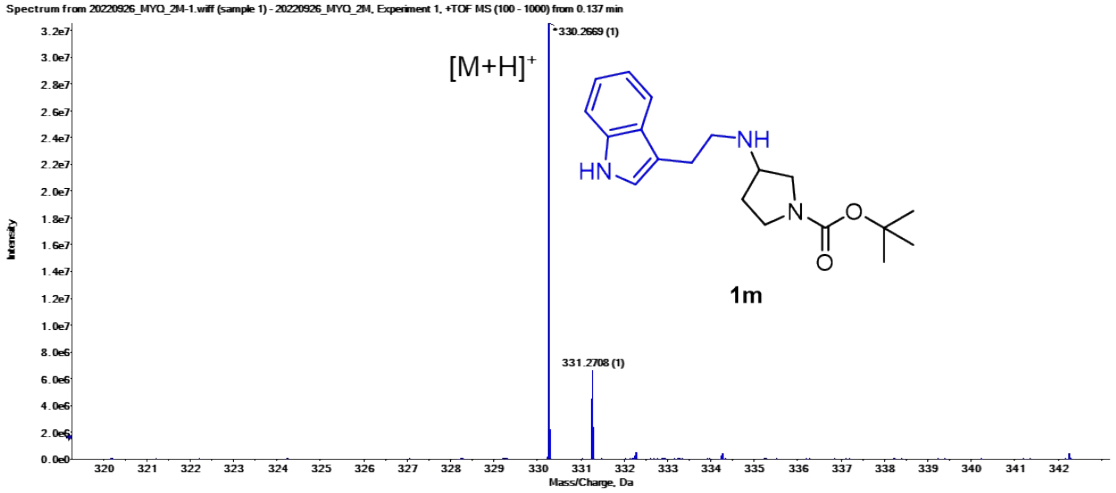


**Figure 58.** The HR-ESI-MS spectrum of **1m**.

## References


1. Hoegenauer, K.; Soldermann, N.; Zecri, F.; Strang, R. S.; Graveleau, N.; Wolf, R. M.; Cooke, N. G.; Smith, A. B.; Hollingworth, G. J.; Blanz, J.; Gutmann, S.; Rummel, G.; Littlewood-Evans, A.; Burkhart, C., *ACS Med. Chem. Lett.,* **2017,** *8*, 975-980.

2. Chough, C.; Joung, M.; Lee, S.; Lee, J.; Kim, J. H.; Kim, B. M., *Bioorg. Med. Chem.,* **2018,** *26*, 1495-1510.
